# An Atlas of Human Regulatory T Helper-like Cells Reveals Features of Th2-like Tregs that Support a Tumorigenic Environment

**DOI:** 10.1016/j.celrep.2017.06.079

**Published:** 2017-07-18

**Authors:** Leena Halim, Marco Romano, Reuben McGregor, Isabel Correa, Polychronis Pavlidis, Nathali Grageda, Sec-Julie Hoong, Muhammed Yuksel, Wayel Jassem, Rosalind F. Hannen, Mark Ong, Olivia Mckinney, Bu’Hussain Hayee, Sophia N. Karagiannis, Nicholas Powell, Robert I. Lechler, Estefania Nova-Lamperti, Giovanna Lombardi

**Affiliations:** 1 MRC Centre for Transplantation, King’s College London, Guy’s Hospital, SE1 9RT London, UK; 2 St. John’s Institute of Dermatology, King’s College London, Guy’s Hospital, SE1 9RT London, UK; 3 Institute of Liver Studies and Transplantation, King’s College London, King’s College Hospital, SE5 9RS London, UK; 4 Centre for Cell Biology and Cutaneous Research, The Blizard Institute, Barts and the London School of Medicine and Dentistry, E1 2AT London, UK; 5 Histology/Histopathology Laboratory, King’s College Hospital, SE5 9RS London, UK; 6 Department of Gastroenterology, King’s College Hospital, SE5 9RS London, UK; 7 King’s Health Partners, SE1 9RT London, UK

**Keywords:** T helper-like regulatory cells, chemokine receptor, tumor immunity, immunoregulation, tumor immunology

## Abstract

Regulatory T cells (Tregs) play a pivotal role in maintaining immunological tolerance, but they can also play a detrimental role by preventing antitumor responses. Here, we characterized T helper (Th)-like Treg subsets to further delineate their biological function and tissue distribution, focusing on their possible contribution to disease states. RNA sequencing and functional assays revealed that Th2-like Tregs displayed higher viability and autocrine interleukin-2 (IL-2)-mediated activation than other subsets. Th2-like Tregs were preferentially found in tissues rather than circulation and exhibited the highest migratory capacity toward chemokines enriched at tumor sites. These cellular responses led us to hypothesize that this subset could play a role in maintaining a tumorigenic environment. Concurrently, Th2-like Tregs were enriched specifically in malignant tissues from patients with melanoma and colorectal cancer compared to healthy tissue. Overall, our results suggest that Th2-like Tregs may contribute to a tumorigenic environment due to their increased cell survival, higher migratory capacity, and selective T-effector suppressive ability.

## Highlights

• Memory Tregs can be classified as T helper-like Tregs (Th2, Th17, Th1, and Th1/17)
• Human Th2-like Tregs exhibit the highest viability and IL-2-mediated activation
• Th2-like Tregs are the subset with the highest chemotaxis toward CCL17/22
• Th2-like Tregs are enriched at tumor sites in melanoma and colorectal cancer

## Graphical Abstract

**Figure:**
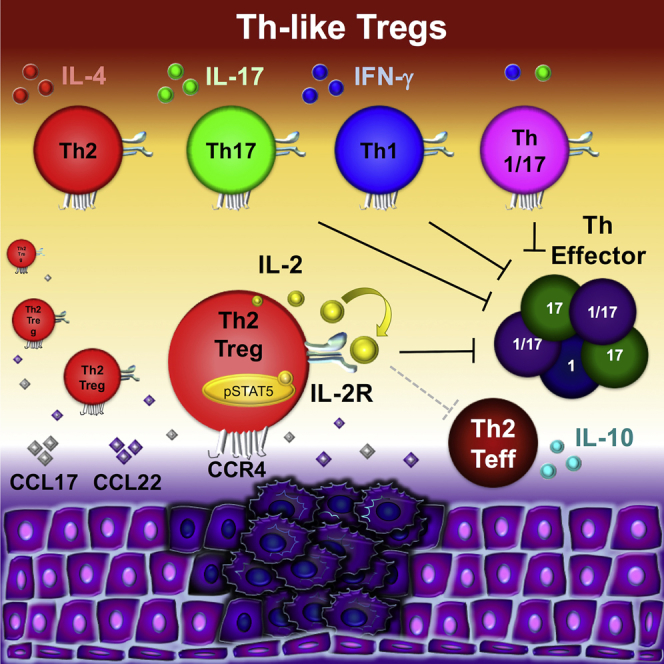

## Teaser

Halim et al. provide a comprehensive transcriptomic and functional analysis of circulating Th-like Tregs, revealing unique features in Th2-like Tregs and a significant enrichment of this subset in patients with melanoma and colorectal cancer. This suggests that Th2-like Tregs play a major role in maintaining a tumorigenic environment.

## Introduction

Regulatory T cells (Tregs) are a subpopulation of T cells that elicit regulatory function by establishing and maintaining immunological tolerance and regulating immune homeostasis (Rosenblum et al., 2016, Sakaguchi et al., 2008). In humans, Tregs contribute to 5%–10% of peripheral CD4^+^ T cells and are highly heterogeneous. In the peripheral circulation, the Treg population is composed of thymic-derived Tregs and Tregs that are induced in the periphery following T cell receptor (TCR) stimulation in a specific cytokine microenvironment (Povoleri et al., 2013). Human Tregs are characterized by the constitutive expression of the interleukin-2 (IL-2) receptor α chain (CD25) and the transcription factor FoxP3, although the same markers are also expressed on activated and antigen experienced non-regulatory effector T cells (Teffs) (Ziegler, 2007). Furthermore, due to its intracellular expression, FoxP3 cannot be used for the isolation of Tregs. Thus far, the identification and isolation of Tregs in peripheral blood has been based on the low expression of the IL-7 receptor α chain (CD127) (Hartigan-O’Connor et al., 2007), as there is an inverse correlation between CD127 and FoxP3, with the most suppressive Tregs expressing low levels of CD127 (Liu et al., 2006). Thus, using a combination of CD4, CD127, and CD25, it is possible to identify and isolate highly pure Tregs. In 2009, Miyara et al. (2009) further categorized Tregs based on the expression of CD4, CD25, FoxP3, and CD45RA. Later, Duhen et al. (2012) described new subpopulations of memory Tregs mirroring the classical CD4^+^ T helper (Th) cells. These new subpopulations, coined Th-like Tregs, express chemokine receptors CXCR3, CCR6, and CCR4, typically expressed by T-bet^+^-Th1, RORγt^+^-Th17, and GATA3^+^-Th2, respectively. The shared homing receptor distribution causes the appropriate co-localization of cell populations in peripheral tissue (Duhen et al., 2012, Erhardt et al., 2011). CCR4 mediates the migration of Tregs to its ligands, CCL17 and CCL22, which are produced by dendritic cells upon maturation, thereby playing a key role in recruiting Tregs into lymphoid tissue (Gobert et al., 2009, Perros et al., 2009). CXCR3 mediates migration to its ligand CXCL10 and may facilitate the recruitment of Tregs into chronically inflamed liver, as liver-infiltrating Tregs expressed higher levels of the receptor than peripheral blood Tregs (Oo et al., 2010). The expression of CCL20, the ligand for CCR6, is induced by IL-17 and secreted by Th17 cells during inflammation and coordinates the migration of Th17 and Tregs to inflammatory sites (Yamazaki et al., 2008). Understanding how chemokines and their cognate receptor orchestrate T cell trafficking and activity is essential in gaining a better interpretation of their role and distribution in health or disease.

A plethora of studies have focused on the role of Tregs in cancer. These regulatory cells can protect and maintain the malignant environment by inhibiting the antitumor immune response (Sugiyama et al., 2013, Zhu et al., 2016). In this pathology, Th1 responses allow secretion of cytokines that promote the antitumor response (Pagès et al., 2005), whereas Th2 responses favor tumor growth (Hou et al., 2013, Pernot et al., 2014). Th2 responses have been correlated with cancer progression in patients with pancreatic cancer (De Monte et al., 2011, Ochi et al., 2012), leukemic cutaneous T cell lymphoma (Guenova et al., 2013), esophageal and gastric cancer (Gabitass et al., 2011), and ovarian cancer (Lutgendorf et al., 2008). The role of Th17 cells in cancer remains controversial (Bailey et al., 2014). Th17 cells are classically pro-inflammatory, but studies have shown that Foxp3^+^IL17^+^ T cells detected in colorectal cancer have the ability to suppress tumor-specific CD8^+^ T cells (Ma and Dong, 2011) and promote the development of cancer-initiating cells (Yang et al., 2011).

In this study, we investigated the immune transcriptome, phenotype, functional responses, and distribution of Th-like Tregs. Our results revealed that Th2-like Tregs were the subset with the highest viability, blasting capacity, and chemotaxis and the widest tissue distribution. Furthermore, they were also the main Treg subset found in tissues and peripheral blood from patients with colorectal cancer and melanoma compared to healthy volunteers. Overall, our data indicate that Th2-like Tregs represent the main Treg population involved in cancer immunology.

## Results

### Identification of Th-like Treg Subsets Based on the Expression of CXCR3, CCR4, and CCR6

**Figure 1:**
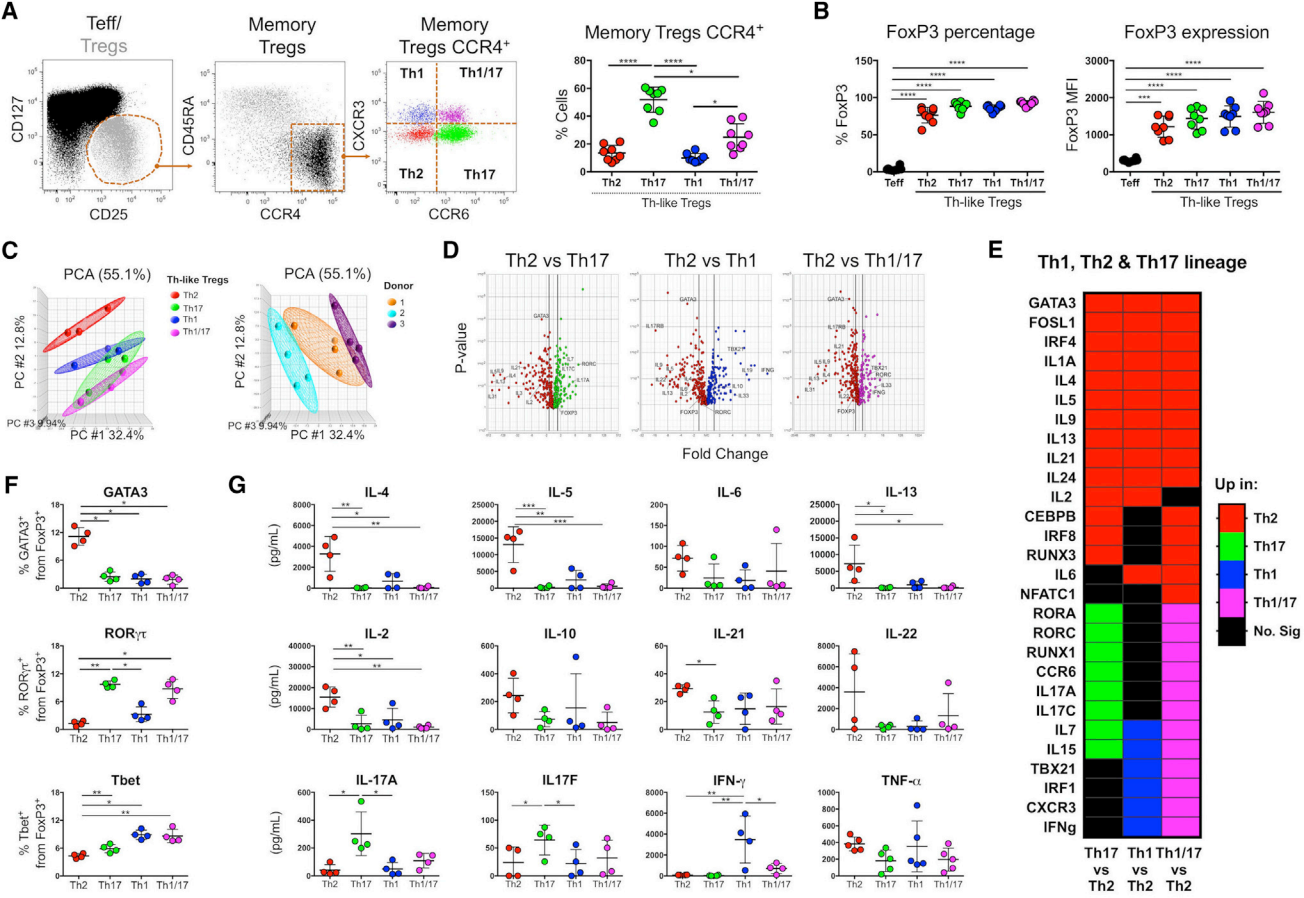
Identification of Four Th-like Tregs Based on CXCR3, CCR4, and CCR6 Expression (A) CCR4, CXCR3, and CCR6 expression was analyzed in memory CD4^+^CD25^hi^CD127^low^CD45RA^−^ Tregs. Four Th-like lineages were identified in the circulation: Th2, Th17, Th1, and Th1/17-like Tregs. (B) FoxP3 expression between Teff and Th-like Treg subsets. For (A) and (B), data are presented as mean ± SEM (n = 8) using independent values (RM one-way ANOVA with Tukey’s test). (C and D) Principal-component analysis (C) and volcano plots (D) showing ANOVA of RNA-seq data obtained from activated Th-like Treg subsets. Thick vertical lines indicate 1.5-fold change threshold (n = 3, using independent values clustered with ellipsoids). (E) Heatmap showing upregulation of Th-lineage genes between Th-like Treg subsets using Partek software. (F and G) Protein expression of GATA3, RORγτ, and T-bet in FoxP3^+^ Treg subsets (F) and absolute values of cytokine production by activated Th-like Treg subsets (G) (n = 4, mean ± SEM using independent values, RM one-way ANOVA with Tukey’s test). For all statistical tests, ^∗∗∗∗^p < 0.0001, ^∗∗∗^p < 0.001, ^∗∗^p < 0.01, and ^∗^p < 0.05 were considered significant.

Circulating peripheral blood mononuclear cells (PBMCs) were used to identify Th-like Treg lineages, as they contain functional representations of Th-like cells from all tissues (Wong et al., 2016). Total Tregs were classified as CD4^+^CD25^hi^CD127^low^ cells, and the proportion of naive and memory Tregs was based on the expression of CD45RA (Figure 1A). Using a novel gating strategy based on CXCR3 and CCR6 expression on CCR4^+^ cells, we evaluated the presence of these markers in naive and memory Tregs. The majority of naive cells were CCR4^−^CXCR3^−^CCR6^−^ (Figure S1A). In contrast, the clear majority of memory Tregs were CCR4^+^, with substantial but differential expression of CXCR3 and CCR6. The expression of these three chemokine receptors allowed the identification of four Th-like lineage subsets in circulation (Figure 1A). We then analyzed FoxP3 expression among these Th-like Treg subsets, and as expected, each subset had a higher frequency and median fluorescence intensity (MFI) than Teffs (Figures 1B and S1B). Furthermore, Th2-like Tregs exhibited the lowest FoxP3 MFI, whereas Th1/17-like Tregs expressed the highest (Figure S1C). Of note, the expression of CD25 and CCR4 did not follow the same pattern of expression as FoxP3 (Figures S1D and S1E). Following targeted RNA-sequencing (RNA-seq) on activated Th-like Treg subsets (in the absence of exogenous IL-2), principal-component analysis indicated Th2-like Tregs cluster separate from the other three Treg subpopulations, independent of donor variability (Figure 1C). Thus, for subsequent analysis, Th2-like Tregs were used as the comparator group. Differential gene expression analysis (Figure 1D) revealed an enrichment of corresponding Th-like genes in each subset and a combination of Th1 and Th17-related genes in Th1/17 Tregs (Figure 1E; Table S1). Gata-3, RORγτ, and T-bet expression was then confirmed by protein expression (Figures 1F and S2A). Lastly, cytokine production was measured in supernatants of activated Th-like Tregs (Figure 1G). In line with the gene expression analysis, Th2-like Tregs produced significantly higher levels of IL-4, IL-5, and IL-13 than other Th-like subsets (Figure 1G). In addition to classical Th2-cytokines, Th2-like Tregs also produced more IL-2 than other subsets (Figure 1G). Higher production of IL-17A and IL-17F was observed in Th17-like Tregs, and higher production of interferon-γ (IFN-γ) was observed in Th1-like Tregs. Intermediate production of IL-17A and IFN-γ was observed in Th1/17 Tregs (Figure 1G). The production of cytokines was consistently and significantly lower in Th-like Tregs than in their Teff counterparts (Figure S2B). Thus, expression of CXCR3, CCR4, and CCR6 allowed us to define four Th-like Tregs in peripheral blood, which matched defining cytokines and transcription factors of their respective lineages.

### Th2-like Tregs Exhibit the Highest Viability and Cytokine-Mediated Activation

**Figure 2:**
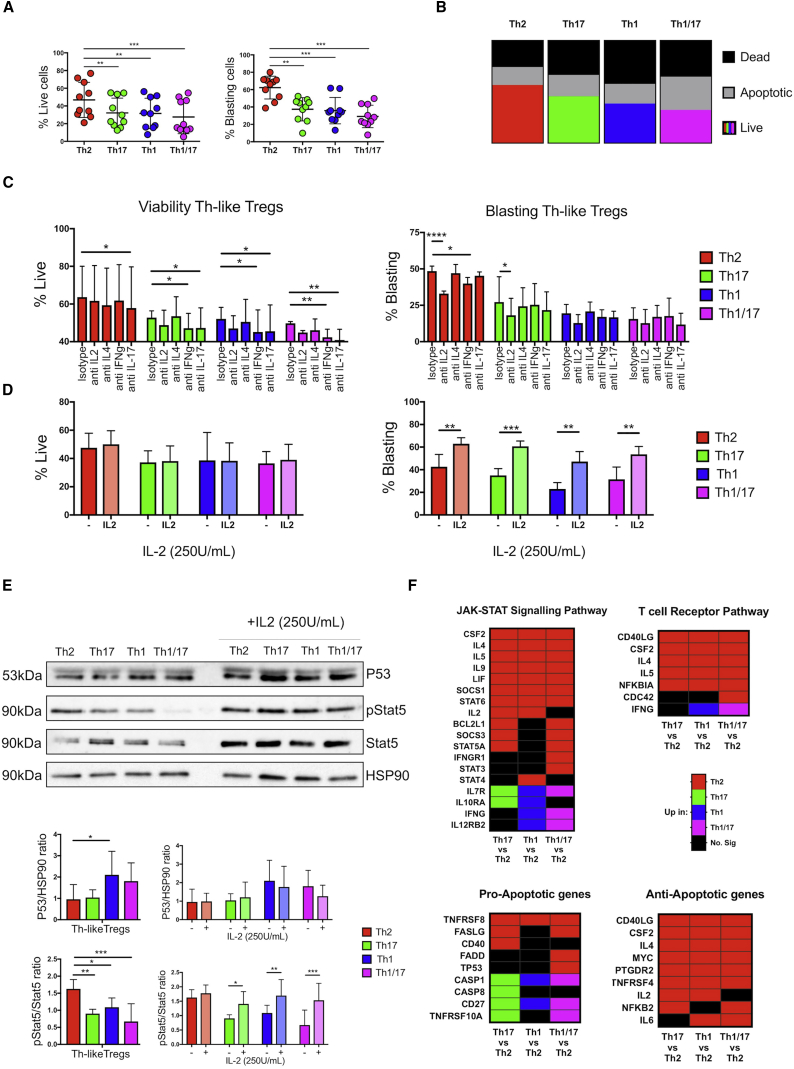
Th2-like Tregs Exhibit Higher Viability, Activation, and JAK-STAT Signaling Pathway Than Other Treg Subsets (A) Total percentages of live and blasting cells between Th-like Tregs 72 hr post-TCR activation in the absence of IL-2 (n = 10, mean ± SEM using independent values, RM one-way ANOVA with Tukey’s test). (B) Distribution of dead, apoptotic, and live cells between Th-like Tregs after TCR activation (n = 5). (C and D) The percentage of live and blasting cells was analyzed in Th-like Treg subsets activated in the presence of neutralizing antibodies for IL-2, IL-4, IFN-γ, and IL-17 (all at 10 μg/mL) (C) or 250 U/ml exogenous IL-2 (D) (n = 4, mean ± SEM using bar charts, RM two-way ANOVA with Tukey’s test). (E) STAT5 signaling and p53 expression was measured in Th-like Tregs 16 hr post-TCR activation in the presence or absence of IL-2 (250 U/mL) (n = 4, mean ± SEM using bar charts, RM two-way ANOVA with Sidak’s test). (F) Heatmap showing upregulation of JAK-STAT, TCR signaling pathway and pro and anti-apoptotic genes between Th-like Treg subsets using Partek software and the KEGG database. For all statistical tests, ^∗∗∗∗^p < 0.0001, ^∗∗∗^p < 0.001, ^∗∗^p < 0.01, and ^∗^p < 0.05 were considered significant.

FoxP3 has been shown to be a pro-apoptotic factor in developing Tregs in the absence of common gamma chain (γc)-dependent cytokine signals (Tai et al., 2013). Since Th2-like Tregs secreted higher levels of γc-dependent cytokine and exhibited the lowest FoxP3 MFI, we evaluated viability and cell activation after TCR engagement. After 3 days, viable, apoptotic, dead, and blasting cells were identified (Figure S3A). Th2-like Tregs showed the highest survival and blasting capacity (Figure 2A) as well as the lowest percentages of combined apoptotic and dead cells (Figure 2B). We next evaluated the effect of cytokines on the viability and blasting of Th-like Treg subsets, observing that IL-2 neutralization significantly reduced the blasting of Th2-like Tregs (Figure 2C), without affecting viability, suggesting that autocrine IL-2 production contributes to higher activation of Th2-like Tregs. Addition of exogenous IL-2 rescued the blasting capacity of the other Treg subsets but did not increase cell survival (Figures 2D and S3B), which was mirrored in total memory Tregs (Figure S3C). IL-2 neutralization also reduced the blasting capacity of total memory Tregs, with no effects on viability (Figure S3D). To confirm this observation, we evaluated p53 expression and STAT5 signaling 16 hr post-activation in the presence or absence of exogenous IL-2. p53 expression was highest in Th1-like Tregs, and its expression was not affected by the addition of exogenous IL-2 in any subset (Figure 2E). Conversely, STAT5 phosphorylation was significantly increased in Th2-like Tregs compared to other subsets in the absence of IL-2, and addition of exogenous IL-2 rescued STAT5 phosphorylation in all Th-like Treg subsets (Figure 2E). These data were backed up by pathway analysis, as genes within JAK-STAT signaling pathways were significantly higher in Th2-like Tregs than in all other Th-like subsets (Figure 2F; Table S1). The TCR-signaling pathway was also evaluated, but no significant difference between Th-like Treg subsets was observed (Figure S4A), suggesting that higher viability was not due to differential TCR activation. Finally, we observed that Th2-like Tregs expressed an anti-apoptotic gene profile, whereas Th17, Th1, and Th1/17 Tregs expressed a more pro-apoptotic gene profile (Figure 2F; Table S1); thus, it is possible that other genes are regulating the higher viability in Th2-like Tregs. Overall, our data suggest that Th2-like Tregs have a survival advantage over other Treg subsets and a higher blasting capacity due to the autocrine IL-2/STAT5 signaling pathway.

### Th-like Treg Subsets Suppress Th-like Teffs, without Preferential Targeting of Their Teff Counterparts

**Figure 3:**
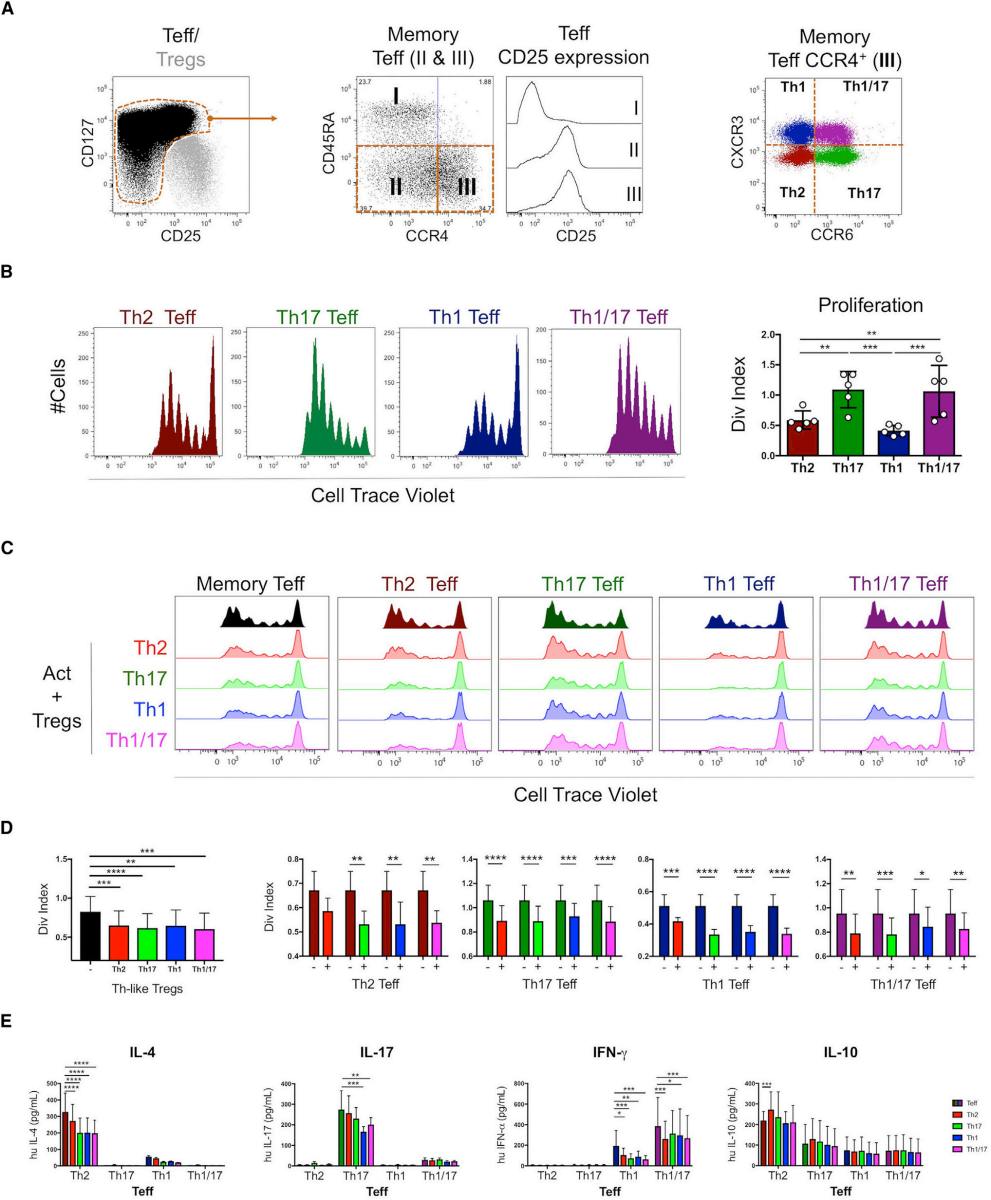
Th-like Tregs Suppress Cell Division of Th-like Teffs without Preference for Lineage Counterparts (A) Representative dot plots of Th-like Teffs. Th2, Th17, Th1, and Th1/17 were identified from memory Teff CCR4^+^ cells. (B) Representative histograms and total percentages of divided Cell Trace Violet^+^ Th-like Teff subsets (1 × 10^5^) stimulated with anti-CD3/CD28 beads at a 40:1 (cell/bead) ratio for 4 days (n = 5, mean ± SEM using bar chart and independent values, RM one-way ANOVA with Tukey’s multiple comparison test). (C and D) Representative histograms (C) and division (Div.) index (D) were obtained from suppression assays between memory Teff or Th-like Teff and Th-like Treg subsets. Teffs (1 × 10^5^) alone or with autologous Tregs (0.5 × 10^5^) were activated with anti-CD3/CD28 beads at a 40:1 (cell/bead) ratio for 4 days. The data are presented as division index obtained from FlowJo analysis (n = 6, mean ± SEM using bar charts, RM two-way ANOVA with Tukey’s test). (E) Absolute values of IL-4, IFN-γ, IL-17, and IL-10 obtained from supernatants after 4 days of suppression assays (n = 6, mean ± SD using bars, RM Two-way ANOVA with Tukey’s test). For all statistical tests, ^∗∗∗∗^p < 0.0001, ^∗∗∗^p < 0.001, ^∗∗^p < 0.01, and ^∗^p < 0.05 were considered significant.

Next, we investigated the capacity of Th-like Treg subsets to suppress their corresponding effector counterparts. Characterization of effector cells revealed that memory Teffs were mainly CD25^int^, and unlike Tregs, they exhibited a substantial percentage of CCR4^−^ cells (Figure 3A). Proliferative ability and cytokine profile of Th-like Teff populations equivalent to the Th-like Treg subsets were analyzed (Figure 3A). After TCR activation, we observed higher proliferation of Th1/17 and Th17 compared to Th2 and Th1-like Teffs (Figure 3B). Cytokine production by Th-like Teff was, as expected, related to Th lineage and similar to the cytokine profiles obtained from Treg subsets (Figure S2B). Next, the ability of Th-like Tregs subsets to inhibit the proliferation of total effector or subpopulations was measured (Figure 3C). Results showed that Th-like Treg subsets reduced cell division of memory Teffs and Th-like Teffs, without preference for inhibition of their Teff counterparts (Figure 3D). Similarly, no inhibition by Treg subsets of lineage-specific cytokines produced by Teffs was observed (Figure 3E). Th-like Treg subsets suppressed pro-inflammatory cytokines, but they did not suppress IL-10, which was produced mainly by Th2-like Teffs (Figure 3E). Interestingly, Th2-like Tregs did not suppress proliferation of Th2-like Teffs as much as other Th-like Treg subsets, possibly due to higher expression of TIGIT, the only protein related to Treg function that is differentially expressed in Th2-like Tregs compared to other subsets after activation (Figure S4B). TIGIT is a co-inhibitory molecule that selectively inhibits pro-inflammatory responses of Th1 and Th17 cells, but not Th2 cells (Joller et al., 2014). Differences in the susceptibility to be suppressed between Teff subsets suggest that their distribution in the site of inflammation is also pertinent in understanding the regulation of the inflammatory response. Since our data showed that all Th-like Tregs suppress memory Teff, we then study the chemotaxis of Th-like Tregs to evaluate whether differences in their regulatory function in vivo may be mediated by differences in their migratory capacity.

### Th2-like Tregs Exhibit Higher Chemotaxis to CCL17/22 Than Other Tregs and Their Counterpart Teffs

**Figure 4:**
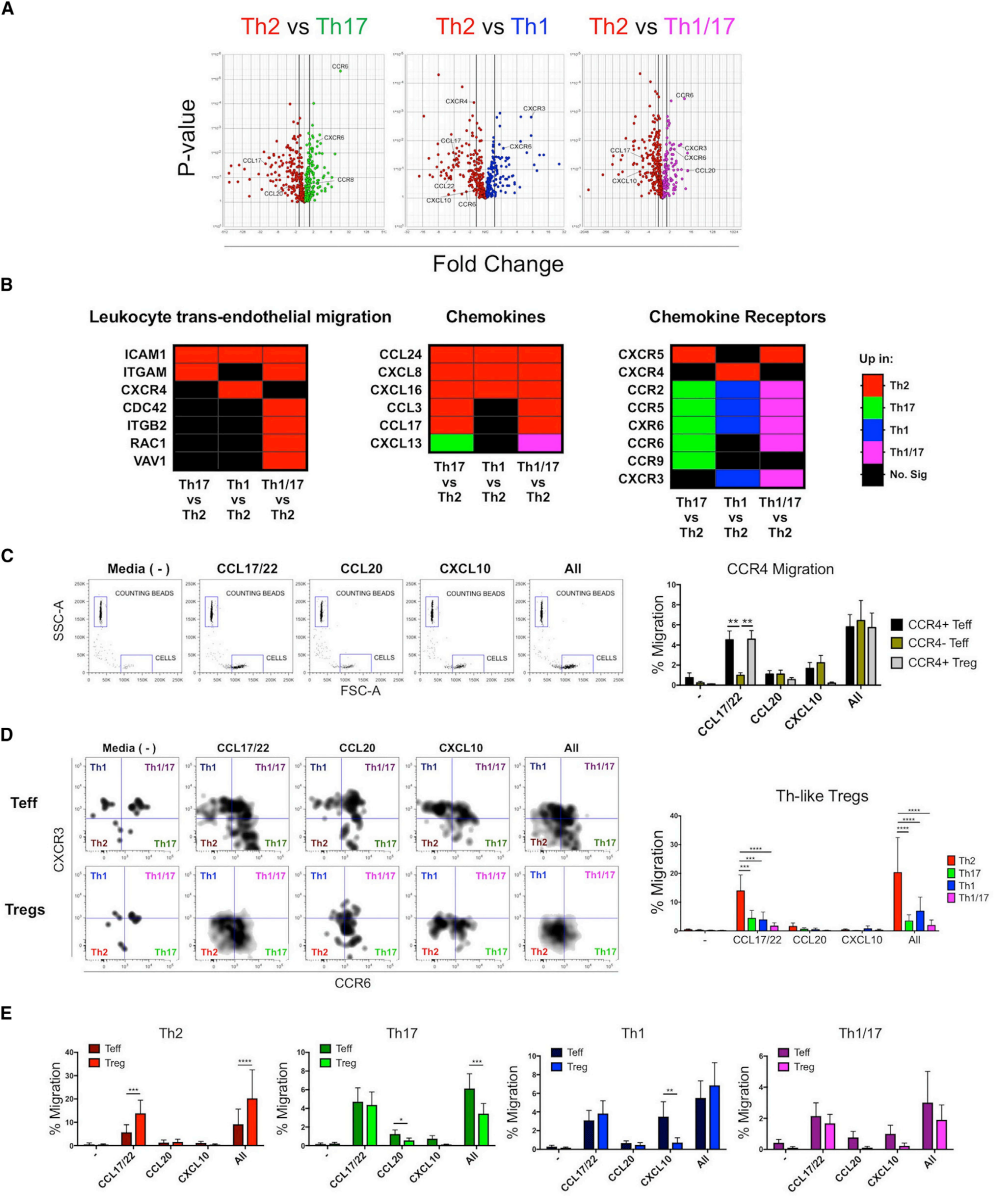
Th2-like Tregs Exhibit Higher Chemotaxis toward CCL17/22 Than Other Th-like Tregs and Th2-like Teffs (A and B) Volcano plots showing RNA-seq data obtained from activated Th-like Treg subsets (A), and heatmaps showing upregulation of leukocyte transendothelial migration, chemokines, and chemokine receptors genes between Th-like Treg subsets using Partek software and the KEGG database (B). (C) Representative dot plots and percentage of migrated memory Teffs and Tregs. Memory Teffs (5 × 10^4^) and memory Tregs (5 × 10^4^) were placed in the top chamber of a 5-μm-pore Transwell filter system with ICAM (1 μg/mL). Bottom chambers were filled with media only; CCL17/22 (0.5 μg/mL), CCL20 (0.5 μg/mL), or CXCL10 (0.5 μg/mL); or a combination of all of them. The percentage of migration for each subset was calculated as (number of cells in the bottom chamber after 1 hr × 100)/initial number of cells in the top chamber. (D and E) Representative dot plots and percentage of migration between Th-like Treg subsets (D) and between CCR4^+^ Th-like Teff and Th-like Tregs (E) (n = 6, mean ± SEM using bar charts, RM two-way ANOVA with Sidak’s test). For all statistical tests, ^∗∗∗∗^p < 0.0001, ^∗∗∗^p < 0.001, ^∗∗^p < 0.01, and ^∗^p < 0.05 were considered significant.

To characterize the migratory ability of the Th-like Tregs, the expression of chemokine receptors by each subtype and the genes associated to migration were evaluated (Figure 4A). Pathway analysis between Th-like Treg subsets revealed higher expression of genes associated with leukocyte trans-endothelial migration in Th2-like Tregs compared to other subsets (Figure 4B; Table S2), suggesting a higher migratory potential in this subset. In addition, we observed a differential expression of chemokines and chemokine receptors between Th-like Tregs (Figure 4B; Table S2). Having characterized the expression of chemokines and their receptors in the different Th-like Treg subsets, cell migration was then assessed using a trans-well system. We observed low migration of cells in the absence of chemokines and a preferential migration of CCR4^+^ cells to chemokines CCL17/22 (Figure 4C). We then evaluated the Th-like phenotype of migrated cells and observed that Th2-like Tregs migrated more than any of the other subsets to CCL17/22 and to a mixture of all the chemokines (Figure 4D). Th2-like Tregs also migrated even more than their Th2-like Teff counterpart in response to the same chemokines (Figure 4E). On the contrary, Th17 and Th1-like Tregs migrate less than their Teff counterparts in response to CCL20 and CXCL10, respectively (Figure 4E). Furthermore, Th2-like Tregs expressed more CCL17 than other Th-like subsets (Figure 4B; Table S2), suggesting that this subset not only migrates more but also has enhanced ability to recruit CCR4^+^ Tregs.

### Th2-like Tregs Are More Prevalent in Tissues and Are the Main Infiltrating Subset Present in Melanoma and Colorectal Cancer

**Figure 5:**
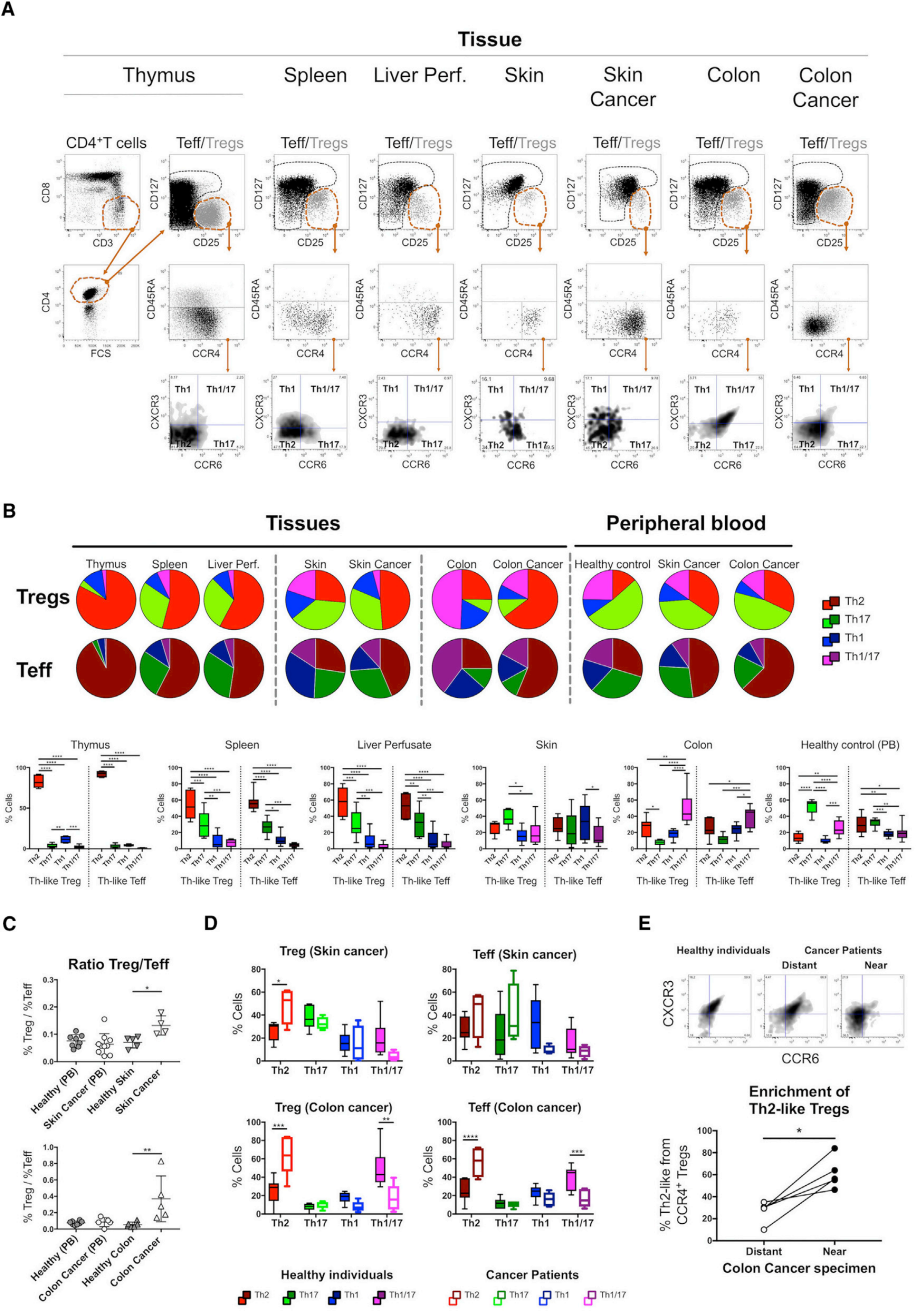
Distribution of Th-like Teff and Treg Subsets in Health and Malignancy (A) Representative plots of chemokine receptor expression in Th-like Tregs obtained from tissues. (B) Pie charts and total percentages of Th-like Tregs and Th-like Tregs Teff in tissues and peripheral blood (mean ± SEM using boxplots, RM two-way ANOVA with Sidak’s test). Thymus = 6, spleen = 8, liver perfusates = 6, healthy skin = 5, skin with cancer = 4, healthy colon = 6, colon with cancer = 5, peripheral blood from healthy donors = 8, peripheral blood from patients with skin cancer = 10, and peripheral blood from patients with colon cancer = 5. (C and D) Treg/Teff ratio (C) and tissue distribution of Th-like cells between healthy individuals and patients with skin or colon cancer (D). In (C), data are presented as mean ± SEM using individual values (one-way ANOVA with Dunnett’s test). In (D), data are presented as mean ± SEM using boxplots (RM two-way ANOVA with Sidak’s multiple comparison test). (E) Representative plots and total percentages of Th2-like Tregs obtained from patients with colorectal cancer. Specimens obtained from tumor sites were compared with samples obtained from distant areas to the tumor (n = 4, independent values, two-tailed t test). ^∗^p < 0.05 was considered significant. For all statistical tests, ^∗∗∗∗^p < 0.0001, ^∗∗∗^p < 0.001, ^∗∗^p < 0.01, and ^∗^p < 0.05 were considered significant.

**Figure 6:**
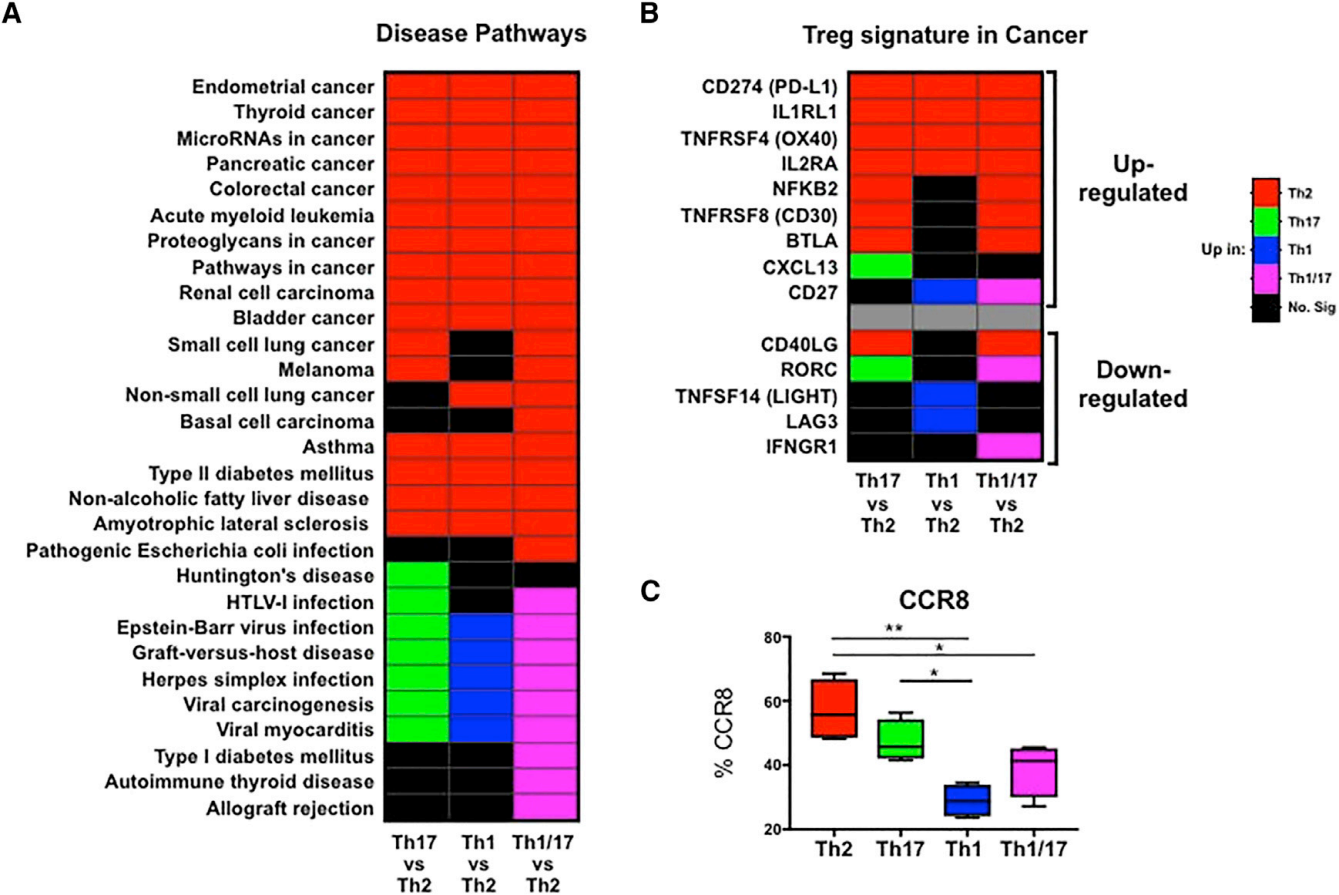
Disease Pathway Analysis of RNA-Seq Data Obtained from Th-like Treg Subsets (A and B) Heatmap showing upregulation of Th-like Treg genes in disease pathways (A) and previously reported upregulated and downregulated genes by tumor infiltrating Tregs (De Simone et al., 2016, Plitas et al., 2016) using Partek software and KEGG database (B). (C) CCR8 expression in Th-like Treg subsets (n = 5, boxplot using minimum to maximum, ordinary one-way ANOVA, with Holm-Sidak’s test). ^∗∗^p < 0.01 and ^∗^p < 0.05 were considered significant.

Th-like Treg subsets expressed distinctive chemokine signatures and exhibited different functional responses. Thus, we evaluated their distribution in primary and secondary lymphoid organs as well as peripheral tissues from healthy volunteers and patients with melanoma or colorectal cancer. We compared the expression of CCR4 between Th-like Tregs and Teff and their distribution in different tissues (Figure 5A) and peripheral blood (Figure 1A). We observed higher percentages of CCR4^+^ cells in Tregs than Teffs in all tissues; conversely, low expression of CCR4 was observed in the thymus (Figure S5A). Next, we dissected the distribution of Th-like Teffs/Tregs in different tissues (Figure 5B). High percentages of Th2-like cells were observed in the spleen, liver perfusate, and thymus, but thymic memory CD4^+^ T cells expressed very low levels of CCR4; therefore, the overall presence of Th-like cells in the thymus was low compared to other tissues. Th17-like Tregs were the main population in the skin, whereas the colon was enriched for Th1/17-like Tregs. In general, Th2-like Tregs were found preferentially in tissues compared to the circulation, even in the skin and colon, supporting the transmigration pathway previously observed (Figure 4B). When samples from patients with cancer (Tables 1 and S3) were analyzed, a higher Treg/Teff ratio was observed in malignant tissue than in healthy tissue (Figure 5C). Furthermore, we observed an increase of Th2-like Tregs and Teffs, concomitant with a reduction in Th1/17 lineages in tissues (Figure 5D) and peripheral blood (Figure S5B) from cancer patients. The production of Th2 cytokines was confirmed by intracellular staining in total CD4^+^ T cells from malignant colon (Figure S5C). Interestingly, the increment of Th2-like cells was more prominent in Teffs than Tregs in patients with colorectal cancer, suggesting that an imbalance in favor of Th2 effector cells may contribute to cancer maintenance. The Th2 phenotype of colon samples from patients with colorectal cancer distant from the cancer area was similar to that obtained from healthy volunteers and significantly different from samples obtained from the cancer area (Figure 5E). Furthermore, our data were supported by the analysis of disease pathways (Figure 6A; Table S4) and previously published signatures from tumors infiltrating Tregs (Figure 6B) (De Simone et al., 2016, Plitas et al., 2016), revealing that Th2-like Treg genes were predominant in pathways associated with cancer. Furthermore, we observed high expression of CCR8 on the surface of resting Th2-like Tregs (Figure 6C), the main chemokine receptor found in Tregs isolated from tumor sites (De Simone et al., 2016, Plitas et al., 2016). Altogether, our phenotypic, genetic, and functional characterization of Th2-like Tregs suggests that this is the main Treg subset involved in cancer immunology.

**Table 1:** Description of Cancer Patients

Patient Data	Melanoma	Colorectal Cancer
Number of patients	12	6
Male	6	4
Female	6	2
Age (y), mean (range)	61.8 (28–89)	62.3 (18–72)
Cancer stage		
I	0	2
IIIA	0	1
IIIC	1	2
IV	11	0
Not applicable	0	1

## Discussion

Here, we provide a comprehensive transcriptomic analysis of circulating Th-like Tregs based on the expression of chemokine receptors, which allows cells to migrate into particular tissues in health and disease (Annunziato et al., 2007, Groom and Luster, 2011, Sugiyama et al., 2013, Yamazaki et al., 2008). Chemokine receptor CCR4 was expressed in all Th-like Treg subsets; however, Th2-like Tregs exhibited higher chemotaxis to CCL17/22 than to other Treg populations. Interestingly, the superior migratory capacity of Th2-like Tregs did not correlate with their CCR4 expression (MFI). However, transcriptome analysis revealed that Th2-like Tregs have higher expression of other genes involved in migration that may imbue them with a better migratory capacity. In addition, Th2-like Tregs express CCR8, which mediates migration to its ligand, CCL17, enhancing their migratory capacity (D’Ambrosio et al., 1998). Whole-genome microarray analysis revealed a selective upregulation of Th2 signature genes, including GATA3, IL4, IL5, and IL13, but a downregulation of IL2RA (CD25) and CCR4 upon downregulation of FoxP3 (Hansmann et al., 2012). This provides a possible explanation as to why Th2-like Tregs exhibited lower CCR4 MFI than other Tregs.

Cytokine production by Th1 and Th17-like Tregs was in line with previous reports (Duhen et al., 2012). Conversely, Th2-like Tregs produced the highest levels of IL-4, IL-5, and IL-13. Duhen et al. (2012), in their Th-like Treg characterization study, could not identify IL-4 or IL-22 by intracellular staining. We circumvented this technical problem by measuring secreted cytokines. Ectopic expression of Foxp3 in conventional T cells has been shown to repress cytokine production (Hori et al., 2003). However, instability of FoxP3 expression in Tregs allows for inflammatory Th cell phenotypes with the ability to secrete IFN-γ and IL-17 (Zhou et al., 2009), IL-4, IL-5, and IL-13 (Hansmann et al., 2012). This is in accordance with our results showing that the Treg population with the highest FoxP3 MFI was also the population with the lowest overall cytokine production. A key question regarding Treg biology is their stability and whether Tregs that express pro-inflammatory cytokine still maintain suppressive capacity. The co-expression of pro-inflammatory and anti-inflammatory cytokines by Th-like Tregs does not appear to have an impact on their suppressive capacity in our system and as previously reported (Duhen et al., 2012, Groom and Luster, 2011, Sugiyama et al., 2013, Yamazaki et al., 2008). It has been shown that Tregs expressing CCR6 are highly suppressive while still producing IL-17 (Duhen et al., 2012, Voo et al., 2009). Moreover, IL-17^+^ Tregs from patients with inflamed intestinal mucosa were also shown to be functionally suppressive (Hovhannisyan et al., 2011, Valmori et al., 2010). All Th-like Tregs suppressed total memory Teffs; however, differences in their suppressive ability were revealed when Th-like Teffs were evaluated. For example, Th2-like Tregs did not reduce the proliferation of Th2 Teffs as significantly as they did with other Teffs. In addition, Th2-like Tregs exhibited higher expression of TIGIT compared to the other Treg subsets. This co-inhibitory molecule that has been shown to selectively inhibit pro-inflammatory Th1 and Th17 cell responses (Joller et al., 2014), supporting the presence of Th2 Teffs in cancer samples. We also observed lower proliferation rates in effector Th2 and Th1-like cells compared to Th17 and Th1/17 Teffs, suggesting that a cellular response meditated by Th17 and Th1/17 lineages could be more potent than a response meditate by Th1-like or Th2 Teffs. In fact, low susceptibility of Th17 and Th1/17 clones to the suppressive ability of total Tregs compare to Th1 and Th2 clones has been reported (Annunziato et al., 2007). This suggests that the presence of Th17 and Th1/17 lineages are favorable in tissues that require higher immune surveillance, whereas a Th2 lineage is favorable in malignant tissues, as they produce IL-10 and IL-4. The secretion of IL-4 is known to inhibit IFN-γ production, Th1 cell differentiation, and Th17 and Th1 responses (Wurtz et al., 2004). Besides Th2-type cytokines, Th2-like Tregs also produced higher levels of IL-2 than other Th-like Treg subsets, but at much lower levels than Teffs. FoxP3 expression has been shown to induce cellular apoptosis and promote cell death in thymic Tregs in the absence of common γc-dependent cytokine signals, especially IL-2 (Tai et al., 2013). IL-4 can also improve proliferation due to a degree of redundancy in the ability of γc-cytokines to maintain functional Tregs (Maerten et al., 2005, Thornton et al., 2010, Yates et al., 2007). Our results showed that despite the fact Th2-like Tregs secreted higher levels of IL-4 than IL-2, the latter was more important for cell activation in vitro, but not for survival, as addition of exogenous IL-2 or neutralization of this cytokine did not affect the percentage of live cells in Th2-like Tregs. Further studies are required to identify the mechanism driving the higher survival; however, one of the highest upregulated genes in Th2-like Tregs when compared with all other Th-like Treg subsets was PTGDR2 (CRTh2), which has been shown to prevent apoptosis under cytokine deprivation (Xue et al., 2009). Altogether, our results suggest that Th2-like Tregs could be more resistant in environments with low levels of IL-2, such as malignant tissues (Giuntoli et al., 2009, Mocellin et al., 2001). In addition to higher viability, Th2-like Tregs also exhibited a higher chemotactic ability than other Treg subsets in response to CCL17/22. A positive correlation between the levels of CCL17 or CCL22 produced by tumor-associated monocytes and the frequency of FoxP3 Tregs in gastric cancer has previously been reported (Mizukami et al., 2008). The migration induced by CCL17 or CCL22 was significantly higher in CD4^+^CD25^+^ cells than in CD4^+^CD25^−^ cells (Mizukami et al., 2008), similar to our migration results. In addition, CCL22 has been shown to divert Tregs and control the growth of melanoma (Klarquist et al., 2016). More recently, poor prognosis in patients with metastatic melanoma due to Th2 polarization has been reported (Enninga et al., 2016). Together, these findings suggest that CCL22 contributes to tumor immunity by recruiting Tregs and Th2 cells. In colorectal cancer, intestinal epithelial cells have the capacity to regulate mucosal T cell trafficking through the release of CCL22 under inflammatory conditions. This allows them to modify the local mucosal cytokine milieu through recruitment of CCR4^+^ T cells that counterbalance the inflammation with the specific production of Th2 cytokines (Berin et al., 2001). In addition, GATA3 is not essential for Treg survival under homeostatic conditions in mice, but GATA3-deficient Tregs do not accumulate at inflamed sites, especially in the gastrointestinal tract compared to other compartments (Wohlfert et al., 2011). Moreover, GATA3-deficient Tregs were not able to prevent colitis in a model of T cell transfer colitis (Wohlfert et al., 2011). Thus, migration of Th2 cells, both Teffs and Tregs, seems to be a mechanism by which the colon maintains gut homeostasis and controls inflammation. This anti-inflammatory response seems to be exacerbated by the tumor to maintain an anti-inflammatory environment, preventing anti-tumor responses and supporting tumor growth. In support of this, two independent studies recently demonstrated higher expression of CCR8 and OX40 in tumor-infiltrating Tregs from breast and lung cancer, colorectal adenocarcinoma, and melanoma (De Simone et al., 2016, Plitas et al., 2016). Previously, CCR8-expressing CD4^+^ T cells have been shown to produce more Th2-type cytokines, such as IL-4, IL-5, IL-9, and IL-13, and less IFN-γ and IL-17 than CCR8^−^CD4^+^T cells (Soler et al., 2006). In addition, the OX40-OX40L pathway is required for Th2 responses (van Wanrooij et al., 2007). Furthermore, most genes upregulated in the Treg signature in cancer were also upregulated in Th2-like Tregs.

Overall, our data suggest that in malignant tissues with increased CCL17/22 secretion, Th2-like Tregs are preferentially attracted to tumor sites, where they display a survival advantage and the ability to inhibit Th1-Th17-Th1/17 effector lineages. Effector Th2 cells also migrate and play a suppressive role, as they secrete IL-10 and IL-4. The data presented here provide further support for studying tumor microenvironments to identify key cellular players maintaining the tumorigenic milieu and possible novel drug targets for tumor immunotherapy.

## Experimental Procedures

### Phenotypic Analysis of Cell Subsets from Peripheral Blood and Tissues

Peripheral blood was obtained from healthy volunteers (age range, 22–36 years; male to female ratio, 3:5) after informed consent was approved. Patients with colorectal cancer (London-Dulwich Research Ethics Committee, reference number 15/LO/1998) and melanoma (King’s College London and St Thomas’ NHS Trust Ethics Committee, reference numbers 08/H0804/139 and 16/LO/0366) were consented in accordance with the Declaration of Helsinki. PBMCs were isolated by density-gradient centrifugation. Isolation protocols of mononuclear cells and research ethics for each tissue are described in Supplemental Experimental Procedures. Patient data are described briefly in Table 1, and more in details can be found in Table S3. The list of all reagents used in this study can be found in Supplemental Experimental Procedures.

### Teff and Treg Isolation from Leukapheresis Blood Cones

RosetteSep Human CD4^+^T Cell Enrichment Cocktail was used to isolate CD4^+^ T cells from leukapheresis cone blood (NHS Blood and Transplant, Colindale, London, UK) obtained from anonymous healthy donors. After negative isolation of CD4^+^ T cells, CD25 MicroBeads II were used to separate Tregs from Teffs. Tregs and Teffs were then sorted on a BD FACSAria I.

### Flow Cytometry

PBMCs and mononuclear cells obtained from tissues were stained with anti-CD4, CD25, CD127, CXCR3, CCR4, CCR6, CD45RA, CD3, CD8, and CCR8 for 30 min at 4°C in the dark. Transcription factor staining was then performed with the Foxp3/Transcription Factor Staining Buffer Set using anti-FoxP3, GATA3, T-bet, and RORγτ for 30 min at 4°C in the dark. Samples were acquired on LSR Fortessa and files analyzed using FlowJo (Tree Star). Gates were set based on biological controls and fluorescence minus one controls.

### RNA-Seq Targeted Panel

Fluorescence-activated cell sorting (FACS)-sorted Th-like Tregs (2 × 10^5^) were activated with CD3/CD28 beads (ratio 1:4) for 72 hr. Cells were lysed in TRIzol, and RNA was isolated with Direct-Zol RNA MicroPrep w/Zymo-Spin columns. RNA-seq was performed using the QIAGEN Human Inflammation and Immunity Transcriptome RNA targeted panel. Samples were quantified with the Agilent High Sensitivity DNA Kit and sequenced with the Illumina MiSeq using MiSeq Reagent Kit v3 (150-cycle) (Illumina). Principal-component analysis, volcano plots, and pathway analysis were performed using QIAseq targeted RNA data analysis tools (QIAGEN) and Partek Genomics Suite software, version 6.6.

### Viability, Blasting, and Cytokine Analysis

FACS-sorted Th-like Tregs (0.5–1 × 10^5^), total memory Tregs (0.5 × 10^5^) and Teffs (1 × 10^5^) were stimulated with anti-CD3/CD28 beads at a 4:1 (cell/bead) ratio in the absence or presence of the neutralizing antibodies anti-IL-2 (10 μg/mL, BioSource International), anti-IL-4, anti-IFN-γ, or anti-IL-17 (all 10 μg/mL, R&D Systems) or in the presence of different concentrations of exogenous IL-2 (Novartis). After 16 hr, STAT5 and p53 were evaluated using western blot. After 72 hr, viability and apoptosis were evaluated using the LIVE/DEAD Fixable Near-IR Dead Cell Stain Kit and Annexin V, and supernatants were used to detect human T cell cytokine production.

### Suppression Assay

FACS-sorted Th-like Teff subpopulations were labeled with 5 μM Cell Trace Violet for 37°C for 15 min. 1 × 10^5^ Teff subpopulations were plated alone and in co-culture with autologous Tregs at 1:2 (Treg/Teff) ratio. Cells were activated with anti-CD3/CD28 beads at a 40:1 (cell/bead) ratio. Cellular proliferation was assessed after 4 days by flow cytometry, and files were analyzed using FlowJo. The data are presented as division index (the total number of divisions divided by the number of cells that went into division) obtained from FlowJo analysis. Supernatant was used to detect human T cell cytokine production using BD Cytometric Bead Array.

### Chemotaxis Assays

T cell migration was assessed using a 5-μm-pore Transwell filter system. The top chambers were incubated with ICAM (1 μg/mL) overnight at 37°C. Cell Trace Violet^+^ memory Teffs and unstained memory Tregs were sorted and rested prior experiment. After resting, 5 × 10^4^ Teffs + 5 × 10^4^ Tregs in 50 μl X-VIVO15 serum-free medium were placed in the top chamber. The bottom chambers were filled with 100 μl X-VIVO15 serum-free only; with CCL17 + CCL22, CCL20, or CXCL10; or with a combination of all of them (all at 0.5 μg/mL, BioLegend). After 1 hr at 37°C, cells were harvested from bottom compartments, stained with anti-CXCR3, anti-CCR4 and anti-CCR6, counted using CountBright Absolute Counting Beads and analyzed by flow cytometry. The percentage of migration for each subset was calculated as (number of Th cells in the bottom chamber after 60 min × 100)/initial number of Th cells in the top chamber.

### Statistical Analysis

Statistical tests were performed using Prism 7 software (GraphPad). Data are expressed as mean ± SD or SEM where applicable using individual values, bar charts, or boxplots. A repeated-measures (RM) two-way ANOVA was used to compare two related variables between Th-like subsets. An RM one-way ANOVA was used to compare one related variable between Th-like Tregs. An ordinary one-way ANOVA was used to compare CCR8 expression between Th-like Tregs. A two-tailed t test was used to compare tumor specimens. Post hoc tests were used as indicated in the figure legends. p values are reported as follows: ^∗^p < 0.05, ^∗∗^p < 0.01, ^∗∗∗^p < 0.001, and ^∗∗∗∗^p < 0.0001.

## Author Contributions

Conceptualization, E.N.-L., L.H., and G.L.; Methodology, E.N.-L. and M.R.; Formal Analysis, E.N.-L. and R.M.; Investigation, E.N.-L., L.H., M.R., R.M., I.C., P.P., N.G., S.-J.H., M.Y., M.H., and O.H.; Resources, G.L., R.F.H., W.J., B.H., G.E.D., S.N.K., and N.P.; Writing – Original Draft, E.N.-L. and L.H.; Writing – Review & Editing, E.N.-L., L.H., M.R., R.M., I.C., P.P., N.P., R.I.L., and G.L.; Visualization, Supervision and Project Administration, E.N.-L.; Funding Acquisition, G.L.

## Accession Numbers

The accession numbers for the Th-like Treg RNA-seq and flow cytometry data reported in this paper are GEO: GSE99733 and Flow Repository: FR-FCM-ZY75, respectively.

## Supplemental Information

Document S1. Supplemental Experimental Procedures, Figures S1–S5 and Tables S1–S4

Document S2. Article plus Supplemental Information
