# Supplementary material for: An Atlas of Human Regulatory T Helper-like Cells Reveals Features of Th2-like Tregs that Support a Tumorigenic Environment
Source: Cell Rep. 2017 Jul 18;20(3):757–70. doi: 10.1016/j.celrep.2017.06.079 (PMC5529316; doi:10.1016/j.celrep.2017.06.079)
Supplement: Document S1. Supplemental Experimental Procedures, Figures S1–S5 and Tables S1–S4 [file mmc1.pdf]

Cell Reports, Volume *20*

Supplemental Information

An Atlas of Human Regulatory T Helper-like Cells Reveals Features of Th2-like Tregs that Support a Tumorigenic Environment

Leena Halim, Marco Romano, Reuben McGregor, Isabel Correa, Polychronis Pavlidis, Nathali Grageda, Sec-Julie Hoong, Muhammed Yuksel, Wayel Jassem, Rosalind F. Hannen, Mark Ong, Olivia Mckinney, Bu'Hussain Hayee, Sophia N. Karagiannis, Nicholas Powell, Robert I. Lechler, Estefania Nova-Lamperti, and Giovanna Lombardi

Supplemental Material

Supplemental Figures

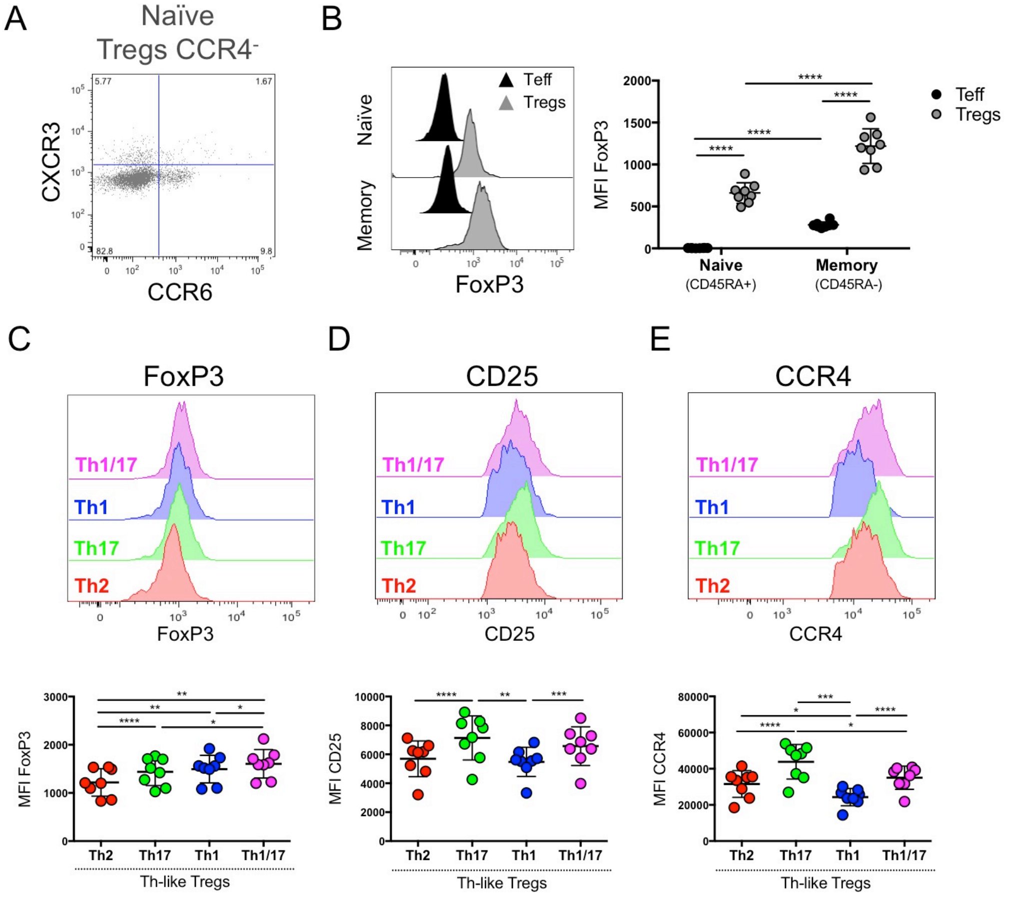

Figure S1: Comparison of FoxP3, CD25 and CCR4 expression between Th-like Teff and Treg subsets from healthy volunteers, related to Figure 1.

(A) Representative histograms of CCR4, CXCR3 and CCR6 expression analysed in naïve CD45RA$^+$ CD4$^+$CD25^{hi}CD127^{low} Tregs. **(B)** Representative histograms and median fluorescence intensity (MFI) of FoxP3 expression in naïve and memory Teff and Tregs [n=8, mean ± SEM using independent values, RM Two-way ANOVA with Sidak's multiple comparison test]. **(C)** Representative histograms and MFI of FoxP3, **(C)** CD25 and **(D)** CCR4 between Th-like Tregs [n=8, mean ± SEM using independent values, RM One-way ANOVA with Tukey's multiple comparison test]. For all statistical tests **** *P*<0.0001, *** *P*<0.001, ** *P*<0.01 and * *P*<0.05 were considered significant.

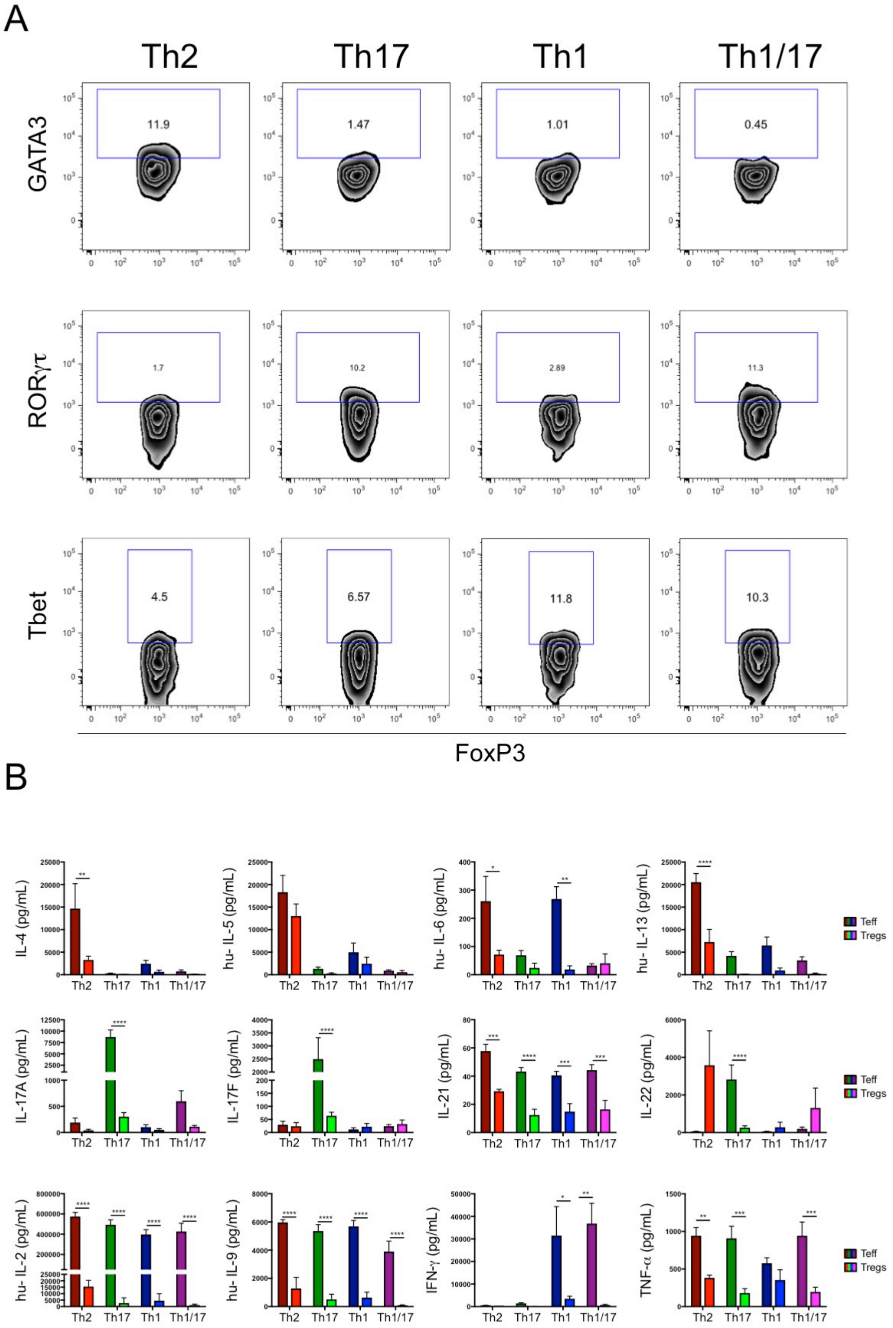

Figure S2: Transcription Factors expression by Th-like Treg subsets, related to Figure 1. (**A**) Representative contour plots of GATA-3, Tbet and RORγτ expression in resting FoxP3$^+$ Th-like Tregs. (**B**) Absolute values of cytokine production by activated Th-like Teff [■Th2, ■Th17, ■Th1 & ■ Th1/17] and Th-like Treg subsets [■Th2, ■Th17, ■Th1 & ■Th1/17] [n=5, mean ± SEM using bar charts, RM Two-way ANOVA with Sidak's test]. For all statistical tests **** P<0.0001, *** P<0.001, ** P<0.01 and * P<0.05 were considered significant.

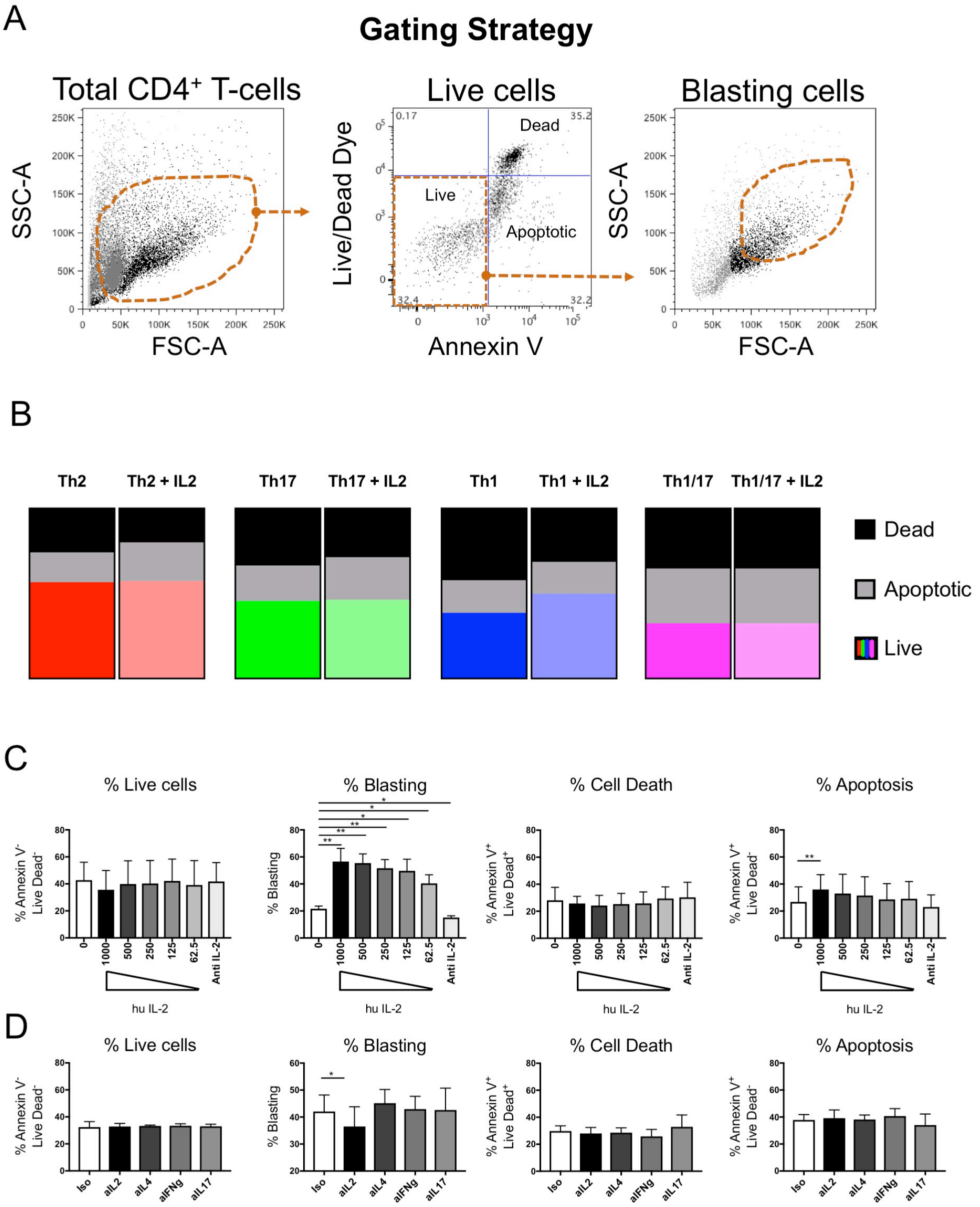

Figure S3: Cytokine effect in Th-like Treg viability and blasting, related to Figure 2.
(**A**) Gating strategy to identify live cells as Live/Dead dye$^-$ Annexin V$^-$, apoptotic cells as Live/Dead dye$^-$ Annexin V$^+$ and dead cells as Live/Dead dye$^+$ Annexin V$^+$ from total CD4$^+$ cells, and then blasting cells among live cells. (**B**) Distribution of dead, apoptotic and live cells between Th-like Tregs in the presence of exogenous IL-2 (250U/mL) [n=5]. (**C**) Percentages of dead, apoptotic, live and blasting cells in total memory Tregs in the presence of exogenous IL-2 (concentration curve) or (**D**) neutralizing antibodies for IL-2, IL-4, IFN-γ and IL-17 (all at 10μg/mL) [n=4, mean ± SD using bars, RM One-way ANOVA with Dunnet's test]. For all statistical tests **** *P*<0.0001, *** *P*<0.001, ** *P*<0.01 and * *P*<0.05 were considered significant.

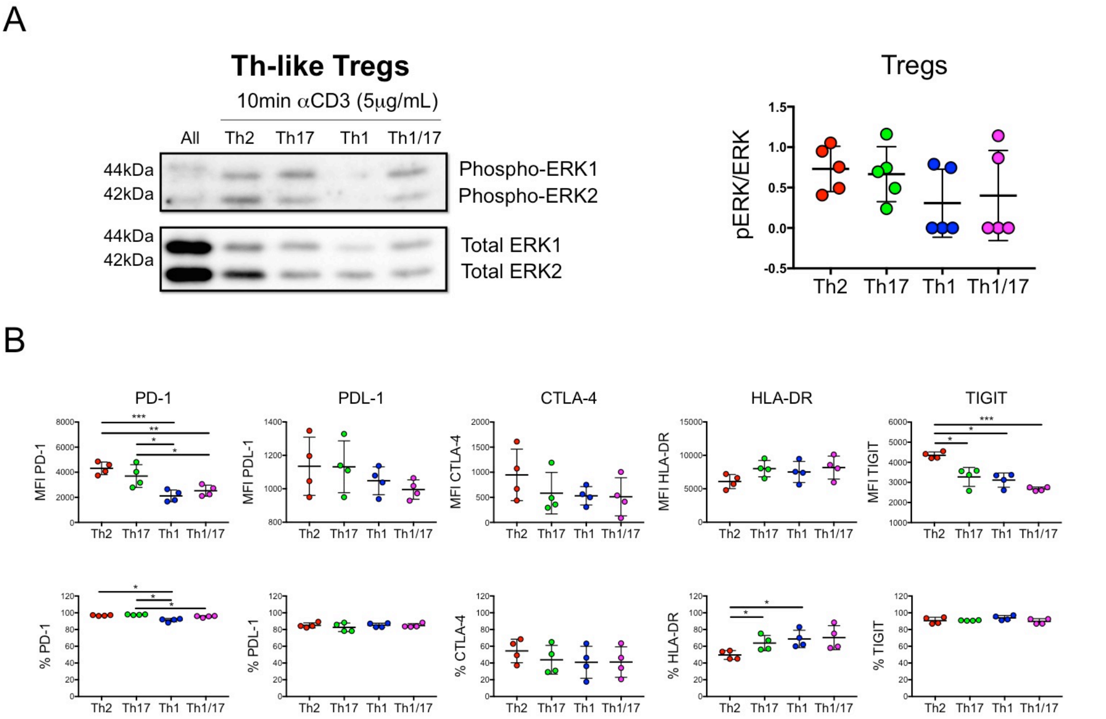

Figure S4: ERK phosphorylation in Th-like Treg and Teff subsets after 10min-post TCR activation, related to Figure 2 and 3.

(A) Representative western blot and absolute number of pERK/ERK ratio between Th-like Tregs. Sorted Th-like Treg subsets were activated with plate bound CD3/CD28 (2μg/mL) on a 96 U-bottom plate for 10min/37°(C). pERK1/2 and ERK1/2 was detected by western blot [n=5, mean ± SEM using independent values, RM One-way ANOVA with Tukey's multiple comparison test]. **(B)** MFI and percentages of PD-1, PDL-1, CTLA-4, HLA-DR and TIGIT in sorted TCR-activated Th-like Treg subsets [n=4, individual values, RM One-way ANOVA with Tukey's multiple comparison test]. For all statistical tests **** $P<0.0001$, *** $P<0.001$, ** $P<0.01$ and * $P<0.05$ were considered significant.

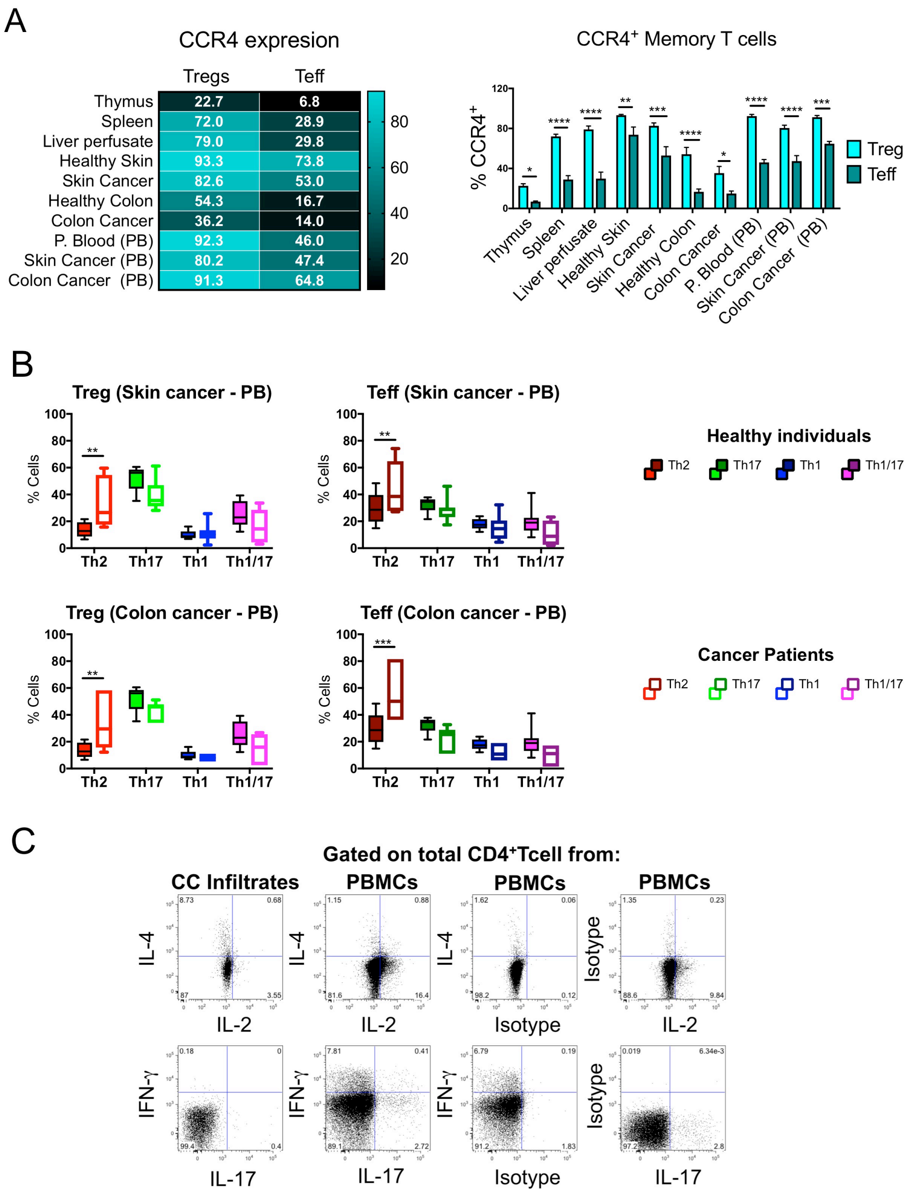

Figure S5: CCR4 expression in Teff and Treg subsets, related to Figure 5.
(**A**) Heat map with average values and total percentages of CCR4^{+} cells within memory Treg or Teff from tissues and peripheral blood (PB) [mean ± SEM using bar charts, Two-way ANOVA with Sidak's test]. (**B**) Th-like distribution between samples obtained from peripheral blood (PB) from healthy individuals and patients with skin or colon cancer [mean ± SEM using box plots, RM Two-way ANOVA with Sidak's multiple comparison test]. **(C)** Intracellular staining of IL-2, IL-4, IFN-γ and IL-17 in total CD4^{+}T-cells from colon cancer (CC) area using CD4^{+}T-cells from PBMCs and isotypes as positive and negative controls.

Supplemental Tables

Th1, Th2 and Th17 lineage

	P-Value	Fold Reg.	Th17 vs Th2	P-Value	Fold Reg.	Th1 vs Th2	P-Value	Fold Reg.	Th1/17 vs Th2
GATA3	1.02E-04	-3.06	Th17 down	1.33E-04	-2.79	Th1 down	8.75E-05	-3.24	Th1/17 down
RORA	9.59E-05	2.07	Th17 up				2.08E-03	1.60	Th1/17 up
RORC	1.08E-02	10.95	Th17 up				1.01E-01	6.29	Th1/17 up
TBX21				1.05E-02	4.71	Th1 up	6.77E-02	3.25	Th1/17 up
CCR6	4.65E-06	16.74	Th17 up				3.45E-04	8.41	Th1/17 up
CXCR3				1.51E-03	7.51	Th1 up	1.48E-02	5.00	Th1/17 up
IL4	4.52E-02	-107.28	Th17 down	1.62E-01	-2.67	Th1 down	4.65E-02	-57.32	Th1/17 down
IL5	3.31E-02	-305.20	Th17 down	5.58E-02	-6.97	Th1 down	3.38E-02	-119.08	Th1/17 down
IL13	6.61E-02	-359.98	Th17 down	1.07E-01	-6.29	Th1 down	6.71E-02	-129.86	Th1/17 down
IL17C	1.00E-02	3.24	Th17 up				5.95E-02	2.40	Th1/17 up
IL17A	6.46E-02	6.43	Th17 up				2.88E-01	3.80	Th1/17 up
IFNG				6.49E-02	25.85	Th1 up	4.71E-01	9.48	Th1/17 up

JAK-STAT and T-cell Receptor Signalling Pathways

	P-Value	Fold Reg.	Th17 vs Th2	P-Value	Fold Reg.	Th1 vs Th2	P-Value	Fold Reg.	Th1/17 vs Th2
BCL2L1	7.44E-02	-1.71	Th17 down				8.62E-02	-1.65	Th1/17 down
CSF2	8.01E-02	-17.48	Th17 down	3.51E-01	-1.83	Th1 down	1.30E-01	-4.69	Th1/17 down
IFNG				6.49E-02	25.85	Th1 up	4.71E-01	9.48	Th1/17 up
IFNGR1							1.43E-03	-1.50	Th1/17 down
IL10RA	1.43E-01	1.50	Th17 up	8.83E-02	1.60	Th1 up			
IL12RB2				3.92E-02	2.51	Th1 up	4.35E-02	2.47	Th1/17 up
IL2	9.32E-02	-3.57	Th17 down	2.31E-01	-1.93	Th1 down			
IL4	4.52E-02	-107.28	Th17 down	1.62E-01	-2.67	Th1 down	4.65E-02	-57.32	Th1/17 down
IL5	3.31E-02	-305.20	Th17 down	5.58E-02	-6.97	Th1 down	3.38E-02	-119.08	Th1/17 down
IL7R	1.28E-01	3.03	Th17 up	6.53E-01	1.54	Th1 up	5.08E-02	3.80	Th1/17 up
IL9	3.09E-02	-162.69	Th17 down	4.12E-02	-12.13	Th1 down	3.26E-02	-49.39	Th1/17 down
LIF	2.65E-02	-5.34	Th17 down	4.11E-02	-3.57	Th1 down	3.42E-02	-4.14	Th1/17 down
SOCS1	6.25E-03	-3.30	Th17 down	3.05E-02	-1.91	Th1 down	7.10E-03	-3.10	Th1/17 down
SOCS3	2.11E-02	-6.09	Th17 down				3.14E-02	-4.05	Th1/17 down
STAT3							5.61E-03	-1.81	Th1/17 down
STAT4				1.35E-03	-1.76	Th1 down			
STAT5A	3.31E-02	-1.52	Th17 down				1.64E-02	-1.69	Th1/17 down
STAT6	1.07E-01	-1.53	Th17 down	9.96E-02	-1.56	Th1 down	4.96E-02	-1.82	Th1/17 down
CDC42							1.13E-02	-1.64	Th1/17 down
NFKBIA	6.41E-02	-2.78	Th17 down	2.67E-01	-1.53	Th1 down	2.04E-01	-1.68	Th1/17 down

Cytokines

	P-Value	Fold Reg.	Th17 vs Th2	P-Value	Fold Reg.	Th1 vs Th2	P-Value	Fold Reg.	Th1/17 vs Th2
IL1A	5.14E-02	-3.05	Th17 down	1.13E-01	-2.06	Th1 down	1.87E-01	-1.70	Th1/17 down
IL2	9.32E-02	-3.57	Th17 down	2.31E-01	-1.93	Th1 down			
IL6				1.79E-01	-2.62	Th1 down	6.09E-02	-15.80	Th1/17 down
IL7	5.40E-03	2.72	Th17 up	1.40E-01	1.69	Th1 up	9.61E-02	1.80	Th1/17 up
IL9	3.09E-02	-162.69	Th17 down	4.12E-02	-12.13	Th1 down	3.26E-02	-49.39	Th1/17 down

**IL15**	3.86E-01	1.64	Th17 up	3.93E-02	2.80	Th1 up	1.98E-02	3.16	Th1/17 up
**IL21**	1.56E-02	-15.16	Th17 down	1.90E-01	-1.71	Th1 down	1.82E-02	-10.01	Th1/17 down
**IL24**	2.48E-02	-9.36	Th17 down	4.66E-02	-4.00	Th1 down	2.04E-02	-16.29	Th1/17 down
**IFNG**				6.49E-02	25.85	Th1 up	4.71E-01	9.48	Th1/17 up
**Pro-apoptotic genes**									
	**P-Value**	**Fold Reg.**	**Th17 vs Th2**	**P-Value**	**Fold Reg.**	**Th1 vs Th2**	**P-Value**	**Fold Reg.**	**Th1/17 vs Th2**
**CASP1**	2.22E-02	1.88	Th17 up	7.50E-02	1.62	Th1 up	1.19E-02	2.02	Th1/17 up
**CASP8**	1.85E-02	1.54	Th17 up						
**CD27**	1.04E-01	2.12	Th17 up	5.05E-02	2.43	Th1 up	4.34E-02	2.49	Th1/17 up
**CD40**	9.52E-02	-1.55	Th17 down						
**FADD**							1.48E-02	-1.98	Th1/17 down
**FASLG**	2.35E-02	-2.62	Th17 down				4.35E-02	-2.09	Th1/17 down
**TNFRSF10A**	1.42E-02	1.69	Th17 up				2.89E-02	1.57	Th1/17 up
**TNFRSF8**	6.10E-02	-2.29	Th17 down	2.16E-01	-1.51	Th1 down	4.79E-02	-2.54	Th1/17 down
**TP53**							1.23E-02	-2.11	Th1/17 down
**Anti-apoptotic genes**									
	**P-Value**	**Fold Reg.**	**Th17 vs Th2**	**P-Value**	**Fold Reg.**	**Th1 vs Th2**	**P-Value**	**Fold Reg.**	**Th1/17 vs Th2**
**CD40LG**	8.44E-02	-1.94	Th17 down	1.13E-01	-1.77	Th1 down	6.13E-02	-2.17	Th1/17 down
**CSF2**	8.01E-02	-17.48	Th17 down	3.51E-01	-1.83	Th1 down	1.30E-01	-4.69	Th1/17 down
**IL2**	9.32E-02	-3.57	Th17 down	2.31E-01	-1.93	Th1 down			
**IL4**	4.52E-02	-107.28	Th17 down	1.62E-01	-2.67	Th1 down	4.65E-02	-57.32	Th1/17 down
**IL6**				1.79E-01	-2.62	Th1 down	6.09E-02	-15.80	Th1/17 down
**MYC**	7.61E-03	-2.44	Th17 down	1.39E-02	-2.06	Th1 down	5.27E-03	-2.77	Th1/17 down
**PTGDR2**	1.11E-02	-10.94	Th17 down	1.29E-02	-8.15	Th1 down	8.28E-03	-35.36	Th1/17 down
**NFKB2**	5.19E-02	-2.17	Th17 down	1.31E-01	-1.64	Th1 down	6.48E-02	-2.01	Th1/17 down
**TNFRSF4**	1.67E-02	-2.87	Th17 down	3.39E-02	-2.18	Th1 down	2.51E-02	-2.43	Th1/17 down
**Transcription Factors**									
	**P-Value**	**Fold Reg.**	**Th17 vs Th2**	**P-Value**	**Fold Reg.**	**Th1 vs Th2**	**P-Value**	**Fold Reg.**	**Th1/17 vs Th2**
**CEBPB**	3.50E-02	-2.86	Th17 down				4.06E-02	-2.65	Th1/17 down
**FOSL1**	5.95E-02	-2.35	Th17 down	1.41E-01	-1.72	Th1 down	9.21E-02	-1.98	Th1/17 down
**GATA3**	1.02E-04	-3.06	Th17 down	1.33E-04	-2.79	Th1 down	8.75E-05	-3.24	Th1/17 down
**IRF1**				1.10E-03	1.89	Th1 up	1.99E-03	1.80	Th1/17 up
**IRF4**	2.60E-03	-2.42	Th17 down	2.62E-02	-1.53	Th1 down	2.16E-03	-2.55	Th1/17 down
**IRF8**	8.15E-02	-7.11	Th17 down				8.39E-02	-6.71	Th1/17 down
**NFATC1**							2.31E-04	-1.68	Th1/17 down
**RORA**	9.59E-05	2.07	Th17 up				2.08E-03	1.60	Th1/17 up
**RORC**	1.08E-02	10.95	Th17 up				1.01E-01	6.29	Th1/17 up
**RUNX1**	6.53E-02	2.05	Th17 up				2.26E-01	1.63	Th1/17 up
**RUNX3**	1.57E-02	-1.64	Th17 down				4.06E-02	-2.65	Th1/17 down
**TBX21**				1.05E-02	4.71	Th1 up	6.77E-02	3.25	Th1/17 up
**Cytokines Receptors**									
	**P-Value**	**Fold Reg.**	**Th17 vs Th2**	**P-Value**	**Fold Reg.**	**Th1 vs Th2**	**P-Value**	**Fold Reg.**	**Th1/17 vs Th2**
**IL1R1**	3.11E-02	2.02	Th17 up						
**IL1RAP**	1.77E-02	-2.27	Th17 down	7.07E-02	-1.61	Th1 down	1.38E-02	-2.46	Th1/17 down
**IL1RL1**	7.20E-02	-12.93	Th17 down	8.20E-02	-8.53	Th1 down	6.76E-02	-17.31	Th1/17 down

**IL1RN**	7.93E-02	-2.50	Th17 down	1.15E-01	-2.09	Th1 down	2.29E-01	-1.61	Th1/17 down
**IL2RA**	3.71E-02	-1.74	Th17 down	5.08E-02	-1.64	Th1 down	1.93E-02	-2.03	Th1/17 down
**IL3RA**	7.96E-02	-7.15	Th17 down	1.06E-01	-4.46	Th1 down	6.55E-02	-12.14	Th1/17 down
**IL4R**	3.81E-03	-1.78	Th17 down	1.93E-03	-2.01	Th1 down	1.70E-03	-2.06	Th1/17 down
**IL6R**	2.63E-02	1.72	Th17 up	1.35E-02	1.84	Th1 up			
**IL7R**	1.28E-01	3.03	Th17 up	6.53E-01	1.54	Th1 up	5.08E-02	3.80	Th1/17 up
**IL9R**	1.25E-02	-10.82	Th17 down	2.60E-02	-4.13	Th1 down	1.07E-02	-16.97	Th1/17 down
**IL10RA**	1.43E-01	1.50	Th17 up	8.83E-02	1.60	Th1 up			
**IL12RB1**	4.79E-02	1.60	Th17 up						
**IL17RA**	1.18E-01	1.63	Th17 up	1.61E-01	1.55	Th1 up	7.44E-02	1.75	Th1/17 up
**IL17RB**	4.94E-03	-3.61	Th17 down	1.39E-03	-15.17	Th1 down	1.44E-03	-13.99	Th1/17 down
**IL17RE**	3.39E-02	2.78	Th17 up	5.58E-01	-1.68	Th1 down			
**IFNGR2**	5.41E-04	-2.33	Th17 down	5.02E-05	-7.90	Th1 down	4.58E-05	-8.89	Th1/17 down
**LEPR**	7.44E-04	1.89	Th17 up				7.91E-03	1.55	Th1/17 up
**LTA**	5.63E-02	-3.91	Th17 down	1.85E-01	-1.90	Th1 down	1.35E-01	-2.19	Th1/17 down
**TNFSF4**	6.54E-03	1.91	Th17 up	5.91E-02	1.52	Th1 up			
**TNFSF11**	1.22E-02	-5.20	Th17 down	2.36E-02	-3.19	Th1 down	9.27E-03	-7.11	Th1/17 down
**TNFSF13**							6.79E-02	-1.73	Th1/17 down
**TNFSF13B**	3.92E-02	4.80	Th17 up	1.71E-01	3.25	Th1 up	1.34E-02	6.02	Th1/17 up
**TNFSF14**	2.33E-02	-3.05	Th17 down	1.80E-02	-3.53	Th1 down	4.04E-02	-2.38	Th1/17 down
**TNFRSF14**				1.53E-03	1.65	Th1 up			

Table S1: Inflammatory & immunity transcriptome data set of activated Th-like Treg subsets: Lineage and activation pathways, related to Figure 1, 2 and 6.
Heat map table showing p values and fold-regulation of RNA-Seq data set obtained from Th-like Treg subsets 3 days post-TCR activation using Partek® Software. Th17, Th1 and Th1/17 Th-like Treg subsets were Test Groups, whereas Th2-like Tregs was the Control Group. *Exclusion criteria:* Genes with fold change <1.5 or with average molecular tag count <10, in both the Control and Test Groups or with p value higher than 0.1 in the three Test Groups vs Control are not shown in this table. *Colours:* **RED** represents higher expression in Th2-like Tregs, **GREEN** higher expression in Th17-like Tregs, **BLUE** higher expression in Th1-like Tregs and **PINK** higher expression in Th1/17-like Tregs. Genes were clustered according to the Human Inflammation & Immunity Transcriptome gene list and Partek® Pathway.

Leukocyte trans-endothelial migration									
	P-Value	Fold Reg.	Th17 vs Th2	P-Value	Fold Reg.	Th1 vs Th2	P-Value	Fold Reg.	Th1/17 vs Th2
CDC42							1.13E-02	-1.64	Th1/17 down
CXCR4				4.66E-04	-1.66	Th1 down			
ICAM1	3.99E-02	-3.65	Th17 down	2.35E-01	-1.58	Th1 down	7.78E-02	-2.44	Th1/17 down
ITGAM	5.51E-02	-3.34	Th17 down				1.00E-01	-2.34	Th1/17 down
ITGB2							3.01E-02	-1.69	Th1/17 down
RAC1							7.71E-03	-1.54	Th1/17 down
VAV1							1.66E-02	-1.90	Th1/17 down

Chemokines Receptors									
	P-Value	Fold Reg.	Th17 vs Th2	P-Value	Fold Reg.	Th1 vs Th2	P-Value	Fold Reg.	Th1/17 vs Th2
CCR2	1.81E-01	6.36	Th17 up	3.15E-02	10.88	Th1 up	2.71E-02	11.29	Th1/17 up
CCR5	8.39E-02	4.67	Th17 up	2.61E-02	6.20	Th1 up	1.02E-01	4.41	Th1/17 up
CCR6	4.65E-06	16.74	Th17 up				3.45E-04	8.41	Th1/17 up
CCR9	6.25E-02	1.82	Th17 up						
CXCR3				1.51E-03	7.51	Th1 up	1.48E-02	5.00	Th1/17 up
CXCR4				4.66E-04	-1.66	Th1 down			
CXCR5	7.21E-02	-2.46	Th17 down				2.73E-02	-4.76	Th1/17 down
CXCR6	3.38E-03	2.76	Th17 up	1.84E-02	2.20	Th1 up	2.00E-02	2.18	Th1/17 up

Chemokines									
	P-Value	Fold Reg.	Th17 vs Th2	P-Value	Fold Reg.	Th1 vs Th2	P-Value	Fold Reg.	Th1/17 vs Th2
CCL3	6.14E-02	-9.73	Th17 down				1.36E-01	-3.05	Th1/17 down
CCL17	2.73E-02	-20.63	Th17 down	6.94E-02	-3.62	Th1 down	4.16E-02	-6.53	Th1/17 down
CCL24	2.42E-02	-3.19	Th17 down	1.31E-01	-1.67	Th1 down	1.10E-02	-5.92	Th1/17 down
CXCL8	1.25E-01	-2.24	Th17 down	3.04E-02	-8.05	Th1 down	6.42E-02	-3.37	Th1/17 down
CXCL13	9.79E-02	8.59	Th17 up				8.18E-01	1.93	Th1/17 up
CXCL16	2.82E-02	-2.65	Th17 down	6.08E-02	-1.99	Th1 down	3.51E-02	-2.42	Th1/17 down

Innate & Adaptive Immune Responses									
	P-Value	Fold Reg.	Th17 vs Th2	P-Value	Fold Reg.	Th1 vs Th2	P-Value	Fold Reg.	Th1/17 vs Th2
C3	3.31E-03	-8.63	Th17 down	6.06E-03	-4.52	Th1 down	2.48E-03	-15.98	Th1/17 down
C3AR1	1.05E-02	4.03	Th17 up	6.84E-02	2.83	Th1 up	7.85E-02	2.75	Th1/17 up
CD14	3.16E-02	-5.67	Th17 down	5.73E-02	-3.26	Th1 down	3.93E-02	-4.45	Th1/17 down
CD8A	3.70E-02	-13.79	Th17 down	7.11E-02	-4.17	Th1 down	4.94E-02	-6.79	Th1/17 down
DDX58	8.82E-03	1.85	Th17 up	3.65E-02	1.60	Th1 up	2.67E-02	1.65	Th1/17 up
ELK1	6.23E-04	-1.68	Th17 down				1.92E-04	-2.01	Th1/17 down
GZMA				1.47E-03	4.76	Th1 up			
HLA-C							2.38E-02	1.52	Th1/17 up
HLA-E	6.34E-03	1.79	Th17 up				2.47E-03	1.96	Th1/17 up
MX1	1.08E-02	2.48	Th17 up	3.82E-02	2.07	Th1 up	1.88E-02	2.29	Th1/17 up
NOD2	2.11E-02	-2.57	Th17 down						
TLR9							5.02E-02	-1.63	Th1/17 down
CHUK							1.33E-02	-1.51	Th1/17 down
HMGB1							8.68E-02	-1.76	Th1/17 down
HSPD1	2.76E-03	-2.02	Th17 down	5.33E-03	-1.78	Th1 down	1.32E-03	-2.40	Th1/17 down

MAP3K1	3.79E-02	2.71	Th17 up				3.02E-02	2.82	Th1/17 up
MAP2K3							9.10E-02	-1.66	Th1/17 down
RELB	6.29E-02	-1.80	Th17 down				8.59E-02	-1.67	Th1/17 down
UBE2N							4.69E-02	-1.58	Th1/17 down
PRKCZ	7.54E-03	1.87	Th17 up						
PRKRA							4.95E-03	-1.75	Th1/17 down

Other Genes

	P-Value	Fold Reg.	Th17 vs Th2	P-Value	Fold Reg.	Th1 vs Th2	P-Value	Fold Reg.	Th1/17 vs Th2
ACKR3				5.29E-02	-1.65	Th1 down			
BST2							3.96E-02	-1.61	Th1/17 down
BTLA	8.80E-03	-1.76	Th17 down				6.55E-03	-1.85	Th1/17 down
CBLB							4.52E-02	1.55	Th1/17 up
CD2	1.38E-02	1.53	Th17 up						
CD274	8.80E-03	-1.95	Th17 down	4.46E-03	-2.29	Th1 down	7.43E-03	-2.02	Th1/17 down
CD276	2.74E-02	-2.22	Th17 down						
CD44	2.00E-03	1.88	Th17 up				2.93E-03	1.82	Th1/17 up
CD81							5.45E-02	-1.61	Th1/17 down
CDK2	2.36E-03	-2.22	Th17 down	6.74E-03	-1.79	Th1 down	6.22E-04	-3.44	Th1/17 down
CDKN1B	6.91E-03	1.54	Th17 up						
CIITA							6.79E-02	-1.74	Th1/17 down
CSF1R	2.36E-01	-1.64	Th17 down				7.86E-02	-2.68	Th1/17 down
EOMES				3.12E-02	13.07	Th1 up			
FOXP1	4.56E-03	1.54	Th17 up				3.65E-03	1.57	Th1/17 up
GBP1				3.07E-03	1.88	Th1 up	4.08E-04	2.31	Th1/17 up
GPI	9.90E-03	-1.82	Th17 down	2.21E-02	-1.59	Th1 down	3.97E-03	-2.22	Th1/17 down
HDAC9							2.50E-02	-1.56	Th1/17 down
HIF1A							2.33E-02	-1.51	Th1/17 down
IFI30	7.54E-03	-2.94	Th17 down				1.34E-02	-2.38	Th1/17 down
IFI44	4.09E-03	3.56	Th17 up	3.37E-01	1.59	Th1 up	1.33E-02	2.97	Th1/17 up
IFI6	3.31E-02	1.81	Th17 up	3.77E-02	1.78	Th1 up	9.87E-02	1.57	Th1/17 up
IFITM2	2.08E-03	-2.19	Th17 down	6.76E-03	-1.74	Th1 down	2.26E-03	-2.14	Th1/17 down
IFITM3	2.44E-02	-4.02	Th17 down	1.09E-01	-1.90	Th1 down	1.80E-02	-5.29	Th1/17 down
IGF1	1.11E-01	-2.56	Th17 down				6.37E-02	-3.88	Th1/17 down
IRGM	7.74E-02	2.01	Th17 up	7.71E-02	2.01	Th1 up	2.82E-01	1.56	Th1/17 up
LAG3	1.50E-01	2.06	Th17 up	4.33E-02	2.64	Th1 up	4.04E-01	1.58	Th1/17 up
LGALS3	1.82E-03	3.33	Th17 up	2.23E-01	1.60	Th1 up	1.41E-02	2.50	Th1/17 up
LRP1	1.46E-02	-7.23	Th17 down	2.84E-02	-3.69	Th1 down			
LYN	3.58E-02	-6.90	Th17 down				3.42E-02	-7.48	Th1/17 down
MET	9.03E-03	-23.37	Th17 down	3.40E-02	-3.23	Th1 down	1.01E-02	-14.91	Th1/17 down
MICA							1.43E-03	1.61	Th1/17 up
MIF	3.59E-02	-1.64	Th17 down	3.02E-02	-1.69	Th1 down	9.03E-03	-2.22	Th1/17 down
MX1	1.08E-02	2.48	Th17 up						
OAS1	2.36E-03	1.98	Th17 up						
OSM	1.22E-02	-3.12	Th17 down	1.53E-02	-2.81	Th1 down	9.71E-03	-3.53	Th1/17 down
PSME2	1.20E-02	1.62	Th17 up						
PTPRC	1.70E-01	2.88	Th17 up	1.84E-01	2.81	Th1 up	5.91E-02	3.80	Th1/17 up
S1PR1	4.97E-02	-2.22	Th17 down	1.47E-01	-1.59	Th1 down	2.20E-02	-3.20	Th1/17 down
SELL	5.73E-02	1.79	Th17 up	1.07E-01	1.63	Th1 up			

**SH2D1A**	5.84E-03	-3.99	Th17 down	4.08E-02	-1.87	Th1 down	7.02E-03	-3.57	Th1/17 down
**TAP2**							2.34E-02	-1.53	Th1/17 down
**TIMP1**	6.36E-03	-2.99	Th17 down	1.26E-02	-2.33	Th1 down	1.31E-02	-2.30	Th1/17 down
**TP53INP1**	3.12E-02	2.01	Th17 up	4.23E-02	1.93	Th1 up	2.08E-02	2.12	Th1/17 up
**TXLNA**							1.41E-02	-1.87	Th1/17 down
**TYK2**							9.31E-02	-1.52	Th1/17 down
**UTS2**	4.24E-02	2.33	Th17 up	3.45E-01	1.53	Th1 up	2.99E-02	2.47	Th1/17 down
**VEGFA**	8.98E-02	-1.86	Th17 down	5.92E-02	-2.13	Th1 down	3.73E-02	-2.56	Th1/17 down
**XCR1**				8.06E-02	2.50	Th1 up			
**ZBTB7B**							5.85E-02	-1.65	Th1/17 down

Table S2: Inflammatory & immunity transcriptome data set of activated Th-like Treg subsets: Migration pathways, related to Figure 4 and 6.

Heat map table showing p values and fold-regulation of RNA-Seq data set obtained from Th-like Treg subsets 3 days post-TCR activation using Partek® Software. Th17, Th1 and Th1/17 Th-like Treg subsets were Test Groups, whereas Th2-like Tregs was the Control Group. *Exclusion criteria:* Genes with fold change <1.5 or with average molecular tag count <10, in both the Control and Test Groups or with p value higher than 0.1 in the three Test Groups vs Control are not shown in this table. *Colours:* **RED** represents higher expression in Th2-like Tregs, **GREEN** higher expression in Th17-like Tregs, **BLUE** higher expression in Th1-like Tregs and **PINK** higher expression in Th1/17-like Tregs. Genes were clustered according to the Human Inflammation & Immunity Transcriptome gene list and Partek® Pathway.

Patient	Tissue of Cance	Type of Cancer	Cancer location	Stage AJCC	Age	Gender	Tissue	PBMC	Therapy
1	Skin	Melanoma	Lower leg	IV	84	Male	Yes	No	None
2	Skin	Melanoma	Upper arm	IV	59	Male	Yes	Yes	None
3	Skin	Melanoma	Elbow	IV	77	Female	Yes	Yes	None
4	Skin	Melanoma	Chest Wall	IV	65	Male	No	Yes	None
5	Skin	Melanoma	Left Temple	IV	89	Male	No	Yes	None
6	Skin	Melanoma	Upper arm	IV	28	Female	No	Yes	Dabrafenib
7	Skin	Melanoma	Post Auricular	IV	28	Female	No	Yes	Dabrafenib Trametinib
8	Skin	Melanoma	Leg	IIIC	61	Female	Yes	No	None
9	Skin	Melanoma	Abdomen	IV	70	Female	No	Yes	None
10	Skin	Melanoma	Chest wall	IV	56	Male	No	Yes	None
11	Skin	Melanoma	Unknown	IV	60	Male	No	Yes	None
12	Skin	Melanoma	Leg	IV	70	Female	No	Yes	None
13	Colon	Adenocarcinoma	Sigmoid	I (T1N0M0)	78	Male	Yes	Yes	None
14	Colon	PTLD (B-cell lymphoma)	Sigmoid	N/A	58	Male	Yes	Yes	Tacrolimus, Vedolizumab
15	Colon	Signet ring adenocarcinoma	Rectum	IIIC (T3N2M0)	33	Male	No	Yes	Vedolizumab
16	Colon	Adenocarcinoma	Ascending	IIIC (T4N2M0)	78	Female	Yes	No	None
17	Colon	Adenocarcinoma	Transverse	I (T2N0M0)	69	Male	Yes	Yes	None
18	Colon	Adenocarcinoma	Descending	IIIA (T4N1M0)	55	Female	Yes	Yes	None

Table S3: Description of patient samples, related to Figure 5 and Table 1.
Blood and tissue samples (from and distant from cancer area) were collected from consecutive patients requiring surgery for melanoma colorectal cancer. Pharmacological treatment was stopped at least two weeks before surgery. **AJCC:** American Joint Committee on Cancer. **PTLD:** Post-transplant lymphoproliferative disease. **N/A:** non applicable. Classification of Malignant Tumours (TNM) in colorectal cancer: **T** describes the size of tumor as T0: no signs of tumor, T1: Tumor in lamina propria or submusoca or $\leq$ 2cm, T2: Muscularis propria or > 2cm, T3: Tumor in subserosa, or pericolorectal tissues and T4: Tumor perforates serosa; adjacent structures. **N** describes nearby (regional) lymph

nodes involved: N0: no lymph nodes, N1: ≤ 3 regional lymph nodes and N2: > 3 regional lymph nodes. **M** describes distant metastasis: M0: no metastasis.

	#	P-Value	P-Value	Fold Change	Descrip-tion	P-Value	Fold Change	Descrip-tion	P-Value	Fold Change	Descrip-tion
Pathway Name	Probe Sets	Th Type	Th17 vs. Th2	Th17 vs. Th2	Th17 vs. Th2	Th1 vs. Th2	Th1 vs. Th2	Th1 vs. Th2	Th1/17 vs. TH2	Th1/17 vs. TH2	Th1/17 vs. TH2
Endometrial cancer	6	2.2E-03	2.4E-03	-1.55	TH17 down vs TH2	3.2E-03	-1.5	TH1 down vs TH2	4.2E-04	-1.99	TH1-TH17 down vs TH2
Huntington's disease	3	1.1E-02	1.0E-02	1.18	TH17 up vs TH2	6.6E-01	1.0	TH1 up vs TH2	2.0E-01	-1.08	TH1-TH17 down vs TH2
Thyroid cancer	4	2.0E-02	3.1E-02	-1.43	TH17 down vs TH2	1.3E-02	-1.6	TH1 down vs TH2	4.4E-03	-1.91	TH1-TH17 down vs TH2
MicroRNAs in cancer	24	2.3E-02	1.7E-02	-1.31	TH17 down vs TH2	2.2E-02	-1.3	TH1 down vs TH2	5.1E-03	-1.45	TH1-TH17 down vs TH2
Pancreatic cancer	19	2.4E-02	1.1E-02	-1.26	TH17 down vs TH2	4.4E-02	-1.2	TH1 down vs TH2	6.4E-03	-1.30	TH1-TH17 down vs TH2
Colorectal cancer	10	3.6E-02	4.1E-02	-1.28	TH17 down vs TH2	2.3E-02	-1.3	TH1 down vs TH2	8.2E-03	-1.48	TH1-TH17 down vs TH2
Viral carcinogenesis	39	4.0E-02	3.5E-02	1.25	TH17 up vs TH2	9.2E-02	1.2	TH1 up vs TH2	8.1E-03	1.36	TH1-TH17 up vs TH2
Acute myeloid leukemia	12	4.2E-02	4.3E-02	-1.30	TH17 down vs TH2	3.6E-02	-1.3	TH1 down vs TH2	9.0E-03	-1.53	TH1-TH17 down vs TH2
Type II diabetes mellitus	7	4.6E-02	1.3E-02	-2.46	TH17 down vs TH2	6.6E-02	-1.6	TH1 down vs TH2	1.9E-02	-2.20	TH1-TH17 down vs TH2
Epstein-Barr virus infection	45	4.6E-02	5.4E-02	1.24	TH17 up vs TH2	7.9E-02	1.2	TH1 up vs TH2	8.8E-03	1.39	TH1-TH17 up vs TH2
Herpes simplex infection	74	4.6E-02	7.9E-02	1.18	TH17 up vs TH2	7.3E-02	1.2	TH1 up vs TH2	8.6E-03	1.34	TH1-TH17 up vs TH2
Non-alcoholic fatty liver disease	24	4.8E-02	2.9E-02	-1.32	TH17 down vs TH2	5.4E-02	-1.3	TH1 down vs TH2	1.1E-02	-1.44	TH1-TH17 down vs TH2
Pathogenic Escherichia coli infection	7	5.4E-02	1.6E-01	-1.17	TH17 down vs TH2	6.0E-01	-1.1	TH1 down vs TH2	1.4E-02	-1.47	TH1-TH17 down vs TH2
Renal cell carcinoma	10	5.5E-02	6.2E-02	-1.28	TH17 down vs TH2	4.0E-02	-1.3	TH1 down vs TH2	1.2E-02	-1.51	TH1-TH17 down vs TH2
Proteoglycans in cancer	29	5.6E-02	4.4E-02	-1.30	TH17 down vs TH2	4.1E-02	-1.3	TH1 down vs TH2	1.3E-02	-1.46	TH1-TH17 down vs TH2
Viral myocarditis	19	5.8E-02	5.5E-02	1.29	TH17 up vs TH2	9.2E-02	1.2	TH1 up vs TH2	1.2E-02	1.44	TH1-TH17 up vs TH2
HTLV-I infection	56	5.9E-02	8.2E-02	1.13	TH17 up vs TH2	1.1E-01	1.1	TH1 up vs TH2	1.1E-02	1.23	TH1-TH17 up vs TH2
Basal cell carcinoma	2	6.4E-02	1.0E-01	-1.40	TH17 down vs TH2	1.1E-01	-1.4	TH1 down vs TH2	1.2E-02	-2.11	TH1-TH17 down vs TH2
Non-small cell lung cancer	6	6.6E-02	1.4E-01	-1.26	TH17 down vs TH2	7.5E-02	-1.4	TH1 down vs TH2	1.3E-02	-1.73	TH1-TH17 down vs TH2
Graft-versus-host disease	20	7.2E-02	9.3E-02	1.22	TH17 up vs TH2	8.3E-02	1.2	TH1 up vs TH2	1.4E-02	1.37	TH1-TH17 up vs TH2
Asthma	11	8.0E-02	2.9E-02	-5.23	TH17 down vs TH2	4.8E-02	-3.4	TH1 down vs TH2	3.1E-02	-4.93	TH1-TH17 down vs TH2
Pathways in cancer	57	8.0E-02	2.7E-02	-1.36	TH17 down vs TH2	8.4E-02	-1.2	TH1 down vs TH2	2.9E-02	-1.36	TH1-TH17 down vs TH2
Type I diabetes mellitus	23	8.9E-02	1.4E-01	1.16	TH17 up vs TH2	1.1E-01	1.2	TH1 up vs TH2	1.8E-02	1.30	TH1-TH17 up vs TH2
Bladder cancer	11	1.0E-01	6.6E-02	-1.44	TH17 down vs TH2	5.6E-02	-1.5	TH1 down vs TH2	2.8E-02	-1.65	TH1-TH17 down vs TH2
Autoimmune thyroid disease	26	1.1E-01	1.3E-01	1.19	TH17 up vs TH2	1.3E-01	1.2	TH1 up vs TH2	2.4E-02	1.33	TH1-TH17 up vs TH2
Allograft rejection	24	1.1E-01	1.5E-01	1.19	TH17 up vs TH2	1.2E-01	1.2	TH1 up vs TH2	2.4E-02	1.33	TH1-TH17 up vs TH2
Amyotrophic lateral sclerosis	9	1.5E-01	8.1E-02	-1.29	TH17 down vs TH2	8.6E-02	-1.3	TH1 down vs TH2	4.4E-02	-1.38	TH1-TH17 down vs TH2
Melanoma	6	1.5E-01	7.7E-02	-1.41	TH17 down vs TH2	1.2E-01	-1.3	TH1 down vs TH2	4.1E-02	-1.55	TH1-TH17 down vs TH2
Small cell lung cancer	15	1.5E-01	4.4E-02	-1.73	TH17 down vs TH2	1.5E-01	-1.4	TH1 down vs TH2	7.4E-02	-1.56	TH1-TH17 down vs TH2

Table S4: Pathways ANOVA between Th-like Treg subsets, related to Figure 6.
Results of pathway ANOVA performed using Partek® Genomics Suite® software, version 6.6. Pathways shown are KEGG pathways (number of genes considered as part of pathway in ANOVA

indicated in second column) significantly enriched within at least one comparison of pathway ANOVA (Th17 vs Th2, Th1 vs Th2 or Th1/Th17 vs Th2). Pathways are displayed in ascending order based on overall pathway significance (p-value <0.05 are indicated in orange). *Colours:* **RED** represents higher expression in Th2-like Tregs, **GREEN** higher expression in Th17-like Tregs, **BLUE** higher expression in Th1-like Tregs and **PINK** higher expression in Th1/17-like Tregs (p-value <0.1 are coloured).

Supplemental Experimental Procedures

Culture conditions and sorting strategy
For *in vitro* assays, cells were cultured in X-VIVO15 (Lonza) supplemented with L-Glutamine 2mM, penicillin/streptomycin 100U/mL (both Thermo Fisher) and 10% of Human Serum AB Male (BioWest). Pre-enriched Th-like Treg and Teff were analysed before and after sorting. Th-like Treg and Teff subsets were of >98 purity.

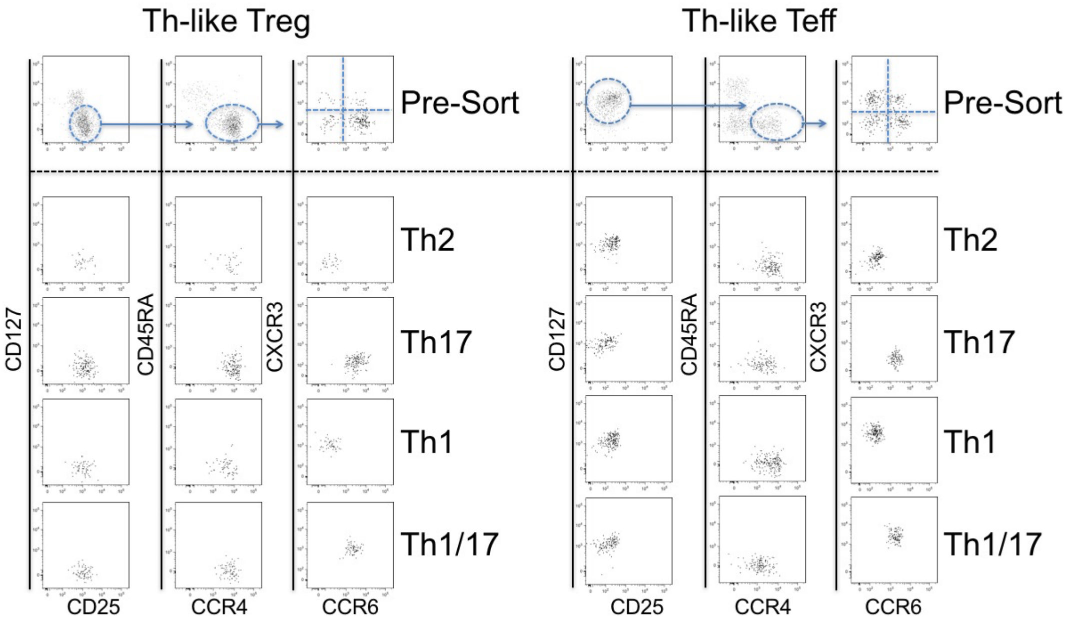

Sorting strategy.

TIGIT, PDL-1, PD-1, HLA-DR, CTLA-4 expression and cytokine analysis
FACS-sorted Th-like Tregs (0.5-1x10^5), total memory Tregs (0.5x10^5) and Teff (1x10^5) were stimulated with anti-CD3/CD28 beads at a 4:1 (cell: bead) ratio. After 72h, TIGIT, PDL-1, PD-1, HLA-DR, CTLA-4 were evaluated using surface staining. Supernatant were used to detect human T-cell cytokine production using LEGENDplex Human Th-Cytokine Assay (BioLegend) and BD Cytometric Bead Array following manufacturer's instructions. Cytokines were acquired on a FACSCanto II (BD Biosciences). Data analysis was carried out on LEGENDplex™ Data Analysis Software or FCAP Array™ Software (BD Biosciences).

ERK Activation and western blot
FACS-sorted subpopulations (1x10^5) were activated with plate bound CD3/CD28 (R&D Systems) (2μg/mL) for 10min/37°C after spin 1800rpm/3min/20°C. Cell lysates were prepared using RIPA buffer (Thermo Fisher) supplemented with protease inhibitors (Calbiochem). Samples were electrophoresed on 10% SDS polyacrylamide gels and transferred to nitrocellulose membranes. After blocking, membranes were incubated with phospho-p44/42 MAPK (Erk1/2) (Thr202/Tyr204) (Cell Signalling) overnight at 4°C. The following day, proteins were detected with chemiluminescence detection reagents (BIORAD) after HRP conjugated secondary antibody incubation using ImageQuant LASS4000 mini (GE Healthcare Life Science), quantified with ImageQuant TL software. Blots were stripped and incubated with p44/42 MAPK (Erk1/2) (Thr202/Tyr204) (Cell Signalling). Same protocol was used for p53 and Stat5 detection.

Intracellular staining
Cells were activated with PMA and Ionomycin for 3h at 37°C. Then, cells were stained with anti-CD4 APC/Cy7, anti-CD3 PerCP/Cy5.5, anti-CD8 Brilliant Violet-711 and anti-CD20 Brilliant Violet-605. Intracellular staining was performed with the Foxp3/Transcription Factor Staining Buffer Set (eBioscience) using anti-FoxP3, anti-IL-2, anti-IL-4, anti-IL-10, anti-IL-17 and anti-IFN-γ 30min/4°C/dark. Samples were then acquired on LSR-Fortessa flow cytometer and files analysed using FlowJo (Tree Star Inc.). Gates were set based on biological, Fluorescent minus one and isotype

controls.

Tissue Collection and Research Ethics Committee

Spleen samples were obtained from deceased human liver or kidney donors and perfusates were obtained from liver grafts at King's College Hospital (both approved by St Thomas' Ethics Committee, reference number: 09/H0802/100). Healthy colon biopsies and colon resections from cancer patients were obtained at King's College Hospital (approved by the London-Dulwich Research Ethics Committee, reference number: 15/LO/1998). Skin was obtained from surgical procedures at Springfield Hospital (approved by the East London and City Health Authority Research Ethics Committee, reference number: 09/HO704/69). Thymuses were collected during infant cardiac surgery at Great Ormond Street Hospital (approved by the Institute of Child Health/Great Ormond Street Hospital Research Ethics Committee, reference number: 07/Q0508/43). Melanoma samples were obtained from surgical procedures at St Thomas (approved by the King's College London and St Thomas' NHS Trust Ethics Committee, reference number: 08/H0804/139 and 16/LO/0366). Informed consent was obtained from all study participants or their representatives in accordance with the Declaration of Helsinki.

-Thymus

Thymuses were washed 3-4 times with PBS, cut into pieces (<0.5cm) and transferred into a gentleMACS tube (Miltenyi Biotec) with X-VIVO15 containing dissociation media (0.2mg/mL of collagenase type XI-S, 0.1mg/mL of DNase1 and 0.5µg/mL Fungizone). Samples were then dissociated using Gentle MACS dissociator (Miltenyi Biotec). Samples were incubated for 15min at 37°C and dissociated again using Gentle MACS dissociator. After dissociation, samples were washed and filtered 3 times using 70µm cell strainers. Cells were counted and $2x10^6$ cells were phenotyped.

-Spleen

Spleens were washed with PBS, cut into pieces <0.5cm and dissociated mechanically with a 2mL syringe in a 50mL Falcon tube containing a 70µm cell strainer. Cells were then washed (2000rpm/10min/4°C), cell pellet was resuspended in PBS and viable splenocytes were isolated using density-gradient centrifugation. Cells were then counted and $2x10^6$ cells were phenotyped.

-Liver Perfusates

Hepatic perfusates were obtained by collecting the second perfusion of the grafts through the portal vein with 1L of saline solution. The collected fluids from the vena cava was stored at 5°C and processed within 6h. Viable hepatic mononuclear cells were isolated using density-gradient centrifugation. Cells were then counted and $2x10^6$ cells were phenotyped.

-Healthy Skin

Skin was washed 3-4 times with PBS, cut into small pieces <0.1cm and transferred into a 20mL pot with X-VIVO15 containing collagenase (1mg/mL) and DNAse (10U/mL). Samples were incubated for 45min at 37°C in agitation. After dissociation, samples were washed, filtered and viable mononuclear cells were isolated using density-gradient centrifugation. Cells were then counted and phenotyped.

-Melanoma (Skin Cancer)

Melanoma specimens were cut in small pieces and mechanically disaggregated using a GentleMACS disociator (Miltenyi Biotec). Single cell suspensions were left overnight at 37°C 5%CO_2 in RPMI media containing 10% Fetal Calf Serum and penicillin/streptomycin 100U/mL. Next day, supernatant samples were collected for cytokine analysis and DNaseI (10U/ml) was added to the cultures for 20min at 37°C. Cells suspensions were then filtered through a 100µm mesh and viable cells were isolated using density-gradient centrifugation. Cells were then counted and phenotyped.

-Colorectal cancer resections (Colon Cancer) and healthy colon biopsies

Colon resections from patients with colorectal cancer and post-transplant lymphoproliferative disease (PLTD in the colon) were obtained after surgery at King's College Hospital. A mucosal sample from the cancer area and a distant sample from the cancer area were obtained and processed similarly to healthy colon biopsies. Briefly, colon mucosa was cut into 3-4mm pieces and incubated with 1mM-EDTA/HBSS 37°C 5%CO_2 for 15min with agitation. Colon samples were then cut into small pieces <1mm and transferred into a 20mL pot with X-VIVO15 containing collagenase (1mg/mL) and DNAse (10U/mL). Samples were incubated for 2h at 37°C in agitation. After dissociation, samples were washed, filtered and viable colonic mononuclear cells were isolated using density-gradient centrifugation. Cells were then counted and $2x10^6$ cells were phenotyped.

Reagents	Catalogue	Company
Agilent High Sensitivity DNA Kit	5067-4626	Agilent Technologies
IL-2 (5344.111) FITC	340448	BD Biosciences
CD4 (SK3) Brilliant Ultra Violet 395	563552	BD Biosciences
Tbet (O4-46) Brilliant Violet 786	564141	BD Biosciences
RORγτ (Q21-559) Alexa Fluor 647	563620	BD Biosciences
CXCR3 (1C6) Alexa Fluor 700	561320	BD Biosciences
CCR8 (433H) [CD198] Brilliant Violet 421	566380	BD Biosciences
CD152 [CTLA-4] (BNI3) APC	555855	BD Biosciences
Stat5 pY694 (47/Stat5(pY694))	611819	BD Biosciences
Stat5 (89/Stat5)	610192	BD Biosciences
BD™ Cytometric Bead Array (CBA) Human Th1/Th2/Th17 cytokine kit	560484	BD Biosciences
Annexin V Alexa Fluor 647	640943	BioLegend
CCR4 (L291H4) PECy7<br>CCR4 (L291H4) PerCP/Cy5.5	359410<br>359406	BioLegend
CCR6 (G034E3) Brilliant Violet 605	353420	BioLegend
CD3 (OKT3) PerCP/Cy5.5	317336	BioLegend
CD4 (OKT4) PerCPCy5.5<br>CD4 (OKT4) Brilliant Violet 421	317428<br>317434	BioLegend
CD8 (SK1) Brilliant Violet 711	344734	BioLegend
CD25 (M-A251) PE	356104	BioLegend
CD45 (HI30) Brilliant Violet 711	304050	BioLegend
CD45RA (H110) APC/Cy7<br>CD45RA (H110) PECy7	304128<br>304126	BioLegend
CD127 (A019D5) APC<br>CD127 (A019D5) Brilliant Violet 711	351318<br>351328	BioLegend
CXCR3 (G025H7) FITC	353704	BioLegend
FoxP3 (259D) Pacific Blue™	320216	BioLegend
GATA3 (16E10A23) Alexa Fluor 647	653810	BioLegend
HLA-DR (L243) Alexa Fluor 647	307621	BioLegend
IL-2 (MQ1-17H12) FITC	500304	BioLegend
LEGENDplex Human Th Cytokine Assay	740001	BioLegend
PD-1 (EH12.2H7) [CD279] Brilliant Violet 421	329919	BioLegend
PDL-1 (29E.2A3) [CD274] [B7-H1] PECy7	329717	BioLegend
Recombinant Human ICAM-1-Fc Chimera	552904	BioLegend
Recombinant Human CCL17 [TARC]	573802	BioLegend
Recombinant Human CCL20 [MIP-3α]	583802	BioLegend
Recombinant Human CXCL10 [IP-10]	573502	BioLegend
Recombinant Human CCL22 [MDC]	584902	BioLegend
TIGIT (A15153G) [VSTM3] APC	372706	BioLegend
Mouse MAb Anti-Human IL-2 (B-G5)	AHC0022	BioSource International
Direct-zol™ RNA MicroPrep Zymo-Spin IC Col.	R2060	Cambridge Bioscience
phospho-p44/42 MAPK [Erk1/2] Thr202/Tyr204	#9101	Cell Signalling
p44/42 MAPK [Erk1/2]	#9102	Cell Signalling
HSP90 (C45G5)	#4877	Cell Signalling
P53 (1C12)	#2524	Cell Signalling
IL-4 (8D4-8) PE	12-7049	eBioscience
IL-17 (eBio64DEC17) PE-Cy7	25-7179	eBioscience
FoxP3/Transcription Factor Staining Buffer	00-5523-00	eBioscience
FoxP3 (PCH10) eFluor 450	48-4776-42	eBioscience
MiSeq Reagent Kit v3	MS-102-3001	Illumina
CD25 MicroBeads II	130-092-983	Milltenyi Biotec
Recombinant IL-2	Proleukin<br>PL 00101/0936	Novartis Pharmaceuticals UK Ltd
The Human Inflammation & Immunity Transcriptome RNA targeted panel 12-Index	RHS-005Z<br>333114	QIAgen
CD3 (UCHT1)	MAB100	R&D

CD28 (37407)	MAB342	R&D
Human IL-4 MAb (34019)	MAB204	R&D
Human IFN-gamma MAb (25718)	MAB285	R&D
Human IL-17 MAb (C41809)	MAB317	R&D
CORNING HTS TRANSWELL-96W	CLS3388-2EA	Sigma-Aldrich
RosetteSep® Human CD4^{+}T Cell Enrich. Cocktail	767 CAD	STEMCELL
CountBright™ Absolute Counting Beads	C36950	Thermo Fisher Scientific
CellTrace™ Violet Cell Proliferation Kit	C34557	Thermo Fisher Scientific
IFN-γ (B27) APC	MHCIFG05	Thermo Fisher Scientific
LIVE/DEAD® Fixable Near-IR Dead Cell Stain Kit	L10119	Thermo Fisher Scientific

Reagents: List of reagents used in this study including clones ( ), alternative names [ ], fluorochromes, catalogue numbers and suppliers.
