# Supplementary material for: An Atlas of Human Regulatory T Helper-like Cells Reveals Features of Th2-like Tregs that Support a Tumorigenic Environment
Source: Cell Rep. 2017 Jul 18;20(3):757–70. doi: 10.1016/j.celrep.2017.06.079 (PMC5529316; doi:10.1016/j.celrep.2017.06.079)
Supplement: Document S2. Article plus Supplemental Information [file mmc2.pdf]

Cell Reports

Resource

An Atlas of Human Regulatory T Helper-like Cells Reveals Features of Th2-like Tregs that Support a Tumorigenic Environment

Graphical Abstract

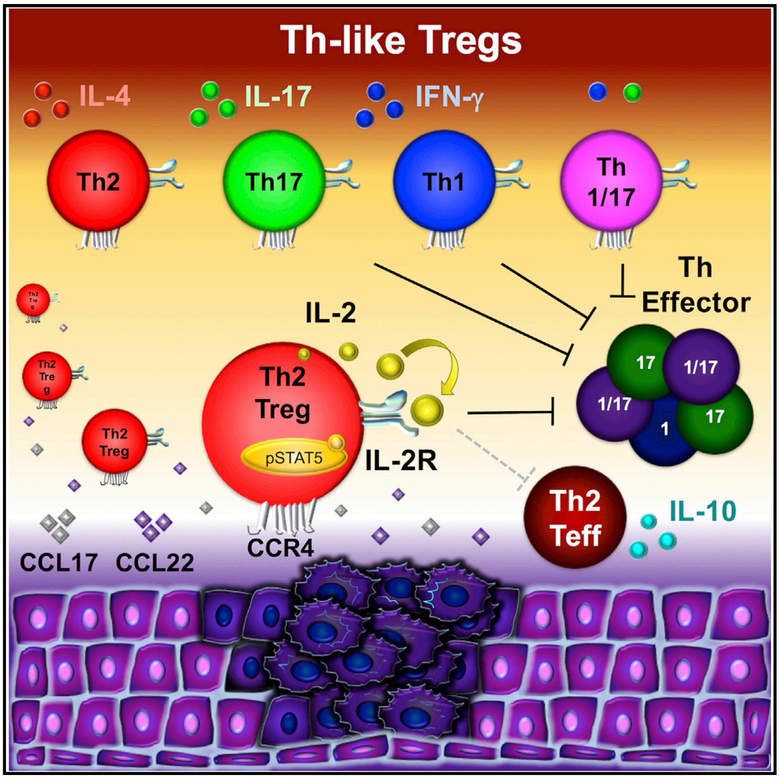

Authors

Leena Halim, Marco Romano, Reuben McGregor, ..., Robert I. Lechler, Estefania Nova-Lamperti, Giovanna Lombardi

Correspondence

enovalamperti@gmail.com (E.N.-L.), giovanna.lombardi@kcl.ac.uk (G.L.)

In Brief

Halim et al. provide a comprehensive transcriptomic and functional analysis of circulating Th-like Tregs, revealing unique features in Th2-like Tregs and a significant enrichment of this subset in patients with melanoma and colorectal cancer. This suggests that Th2-like Tregs play a major role in maintaining a tumorigenic environment.

Highlights

- Memory Tregs can be classified as T helper-like Tregs (Th2, Th17, Th1, and Th1/17)
- Human Th2-like Tregs exhibit the highest viability and IL-2-mediated activation
- Th2-like Tregs are the subset with the highest chemotaxis toward CCL17/22
- Th2-like Tregs are enriched at tumor sites in melanoma and colorectal cancer

Accession Numbers

GSE99733

Halim et al., 2017, Cell Reports *20*, 757–770
July 18, 2017 © 2017 The Authors.
http://dx.doi.org/10.1016/j.celrep.2017.06.079

Cell Reports
Resource

OPEN ACCESS
CellPress

An Atlas of Human Regulatory T Helper-like Cells Reveals Features of Th2-like Tregs that Support a Tumorigenic Environment

Leena Halim,[1,8] Marco Romano,[1,8] Reuben McGregor,[1,8] Isabel Correa,[2] Polychronis Pavlidis,[1] Nathali Grageda,[1] Sec-Julie Hoong,[1] Muhammed Yuksel,[3] Wayel Jassem,[3] Rosalind F. Hannen,[4] Mark Ong,[5] Olivia Mckinney,[5] Bu'Hussain Hayee,[6] Sophia N. Karagiannis,[2] Nicholas Powell,[1] Robert I. Lechler,[1,7] Estefania Nova-Lamperti,[1,9,10,11,*] and Giovanna Lombardi[1,9,*]

[1]MRC Centre for Transplantation, King's College London, Guy's Hospital, SE1 9RT London, UK
[2]St. John's Institute of Dermatology, King's College London, Guy's Hospital, SE1 9RT London, UK
[3]Institute of Liver Studies and Transplantation, King's College London, King's College Hospital, SE5 9RS London, UK
[4]Centre for Cell Biology and Cutaneous Research, The Blizard Institute, Barts and the London School of Medicine and Dentistry, E1 2AT London, UK
[5]Histology/Histopathology Laboratory, King's College Hospital, SE5 9RS London, UK
[6]Department of Gastroenterology, King's College Hospital, SE5 9RS London, UK
[7]King's Health Partners, SE1 9RT London, UK
[8]These authors contributed equally
[9]These authors contributed equally
[10]Present address: Department of Clinical Biochemistry and Immunology, Faculty of Pharmacy, University of Concepción, 4070386 Concepción, Chile
[11]Lead Contact
*Correspondence: enovalamperti@gmail.com (E.N.-L.), giovanna.lombardi@kcl.ac.uk (G.L.)
http://dx.doi.org/10.1016/j.celrep.2017.06.079

SUMMARY

Regulatory T cells (Tregs) play a pivotal role in maintaining immunological tolerance, but they can also play a detrimental role by preventing antitumor responses. Here, we characterized T helper (Th)-like Treg subsets to further delineate their biological function and tissue distribution, focusing on their possible contribution to disease states. RNA sequencing and functional assays revealed that Th2-like Tregs displayed higher viability and autocrine interleukin-2 (IL-2)-mediated activation than other subsets. Th2-like Tregs were preferentially found in tissues rather than circulation and exhibited the highest migratory capacity toward chemokines enriched at tumor sites. These cellular responses led us to hypothesize that this subset could play a role in maintaining a tumorigenic environment. Concurrently, Th2-like Tregs were enriched specifically in malignant tissues from patients with melanoma and colorectal cancer compared to healthy tissue. Overall, our results suggest that Th2-like Tregs may contribute to a tumorigenic environment due to their increased cell survival, higher migratory capacity, and selective T-effector suppressive ability.

INTRODUCTION

Regulatory T cells (Tregs) are a subpopulation of T cells that elicit regulatory function by establishing and maintaining immunological tolerance and regulating immune homeostasis (Rosenblum et al., 2016; Sakaguchi et al., 2008). In humans, Tregs contribute to 5%–10% of peripheral $CD4^+$ T cells and are highly heterogeneous. In the peripheral circulation, the Treg population is composed of thymic-derived Tregs and Tregs that are induced in the periphery following T cell receptor (TCR) stimulation in a specific cytokine microenvironment (Povoleri et al., 2013). Human Tregs are characterized by the constitutive expression of the interleukin-2 (IL-2) receptor α chain (CD25) and the transcription factor FoxP3, although the same markers are also expressed on activated and antigen experienced non-regulatory effector T cells (Teffs) (Ziegler, 2007). Furthermore, due to its intracellular expression, FoxP3 cannot be used for the isolation of Tregs. Thus far, the identification and isolation of Tregs in peripheral blood has been based on the low expression of the IL-7 receptor α chain (CD127) (Hartigan-O'Connor et al., 2007), as there is an inverse correlation between CD127 and FoxP3, with the most suppressive Tregs expressing low levels of CD127 (Liu et al., 2006). Thus, using a combination of CD4, CD127, and CD25, it is possible to identify and isolate highly pure Tregs. In 2009, Miyara et al. (2009) further categorized Tregs based on the expression of CD4, CD25, FoxP3, and CD45RA. Later, Duhen et al. (2012) described new subpopulations of memory Tregs mirroring the classical $CD4^+$ T helper (Th) cells. These new subpopulations, coined Th-like Tregs, express chemokine receptors

This is an open access article under the CC BY license (http://creativecommons.org/licenses/by/4.0/).

CXCR3, CCR6, and CCR4, typically expressed by T-bet$^+$-Th1, RORγt$^+$-Th17, and GATA3$^+$-Th2, respectively. The shared homing receptor distribution causes the appropriate co-localization of cell populations in peripheral tissue (Duhen et al., 2012; Erhardt et al., 2011). CCR4 mediates the migration of Tregs to its ligands, CCL17 and CCL22, which are produced by dendritic cells upon maturation, thereby playing a key role in recruiting Tregs into lymphoid tissue (Gobert et al., 2009; Perros et al., 2009). CXCR3 mediates migration to its ligand CXCL10 and may facilitate the recruitment of Tregs into chronically inflamed liver, as liver-infiltrating Tregs expressed higher levels of the receptor than peripheral blood Tregs (Oo et al., 2010). The expression of CCL20, the ligand for CCR6, is induced by IL-17 and secreted by Th17 cells during inflammation and coordinates the migration of Th17 and Tregs to inflammatory sites (Yamazaki et al., 2008). Understanding how chemokines and their cognate receptor orchestrate T cell trafficking and activity is essential in gaining a better interpretation of their role and distribution in health or disease.

A plethora of studies have focused on the role of Tregs in cancer. These regulatory cells can protect and maintain the malignant environment by inhibiting the antitumor immune response (Sugiyama et al., 2013; Zhu et al., 2016). In this pathology, Th1 responses allow secretion of cytokines that promote the antitumor response (Pagès et al., 2005), whereas Th2 responses favor tumor growth (Hou et al., 2013; Pernot et al., 2014). Th2 responses have been correlated with cancer progression in patients with pancreatic cancer (De Monte et al., 2011; Ochi et al., 2012), leukemic cutaneous T cell lymphoma (Guenova et al., 2013), esophageal and gastric cancer (Gabitass et al., 2011), and ovarian cancer (Lutgendorf et al., 2008). The role of Th17 cells in cancer remains controversial (Bailey et al., 2014). Th17 cells are classically pro-inflammatory, but studies have shown that Foxp3$^+$IL17$^+$ T cells detected in colorectal cancer have the ability to suppress tumor-specific CD8$^+$ T cells (Ma and Dong, 2011) and promote the development of cancer-initiating cells (Yang et al., 2011).

In this study, we investigated the immune transcriptome, phenotype, functional responses, and distribution of Th-like Tregs. Our results revealed that Th2-like Tregs were the subset with the highest viability, blasting capacity, and chemotaxis and the widest tissue distribution. Furthermore, they were also the main Treg subset found in tissues and peripheral blood from patients with colorectal cancer and melanoma compared to healthy volunteers. Overall, our data indicate that Th2-like Tregs represent the main Treg population involved in cancer immunology.

RESULTS

Identification of Th-like Treg Subsets Based on the Expression of CXCR3, CCR4, and CCR6

Circulating peripheral blood mononuclear cells (PBMCs) were used to identify Th-like Treg lineages, as they contain functional representations of Th-like cells from all tissues (Wong et al., 2016). Total Tregs were classified as CD4$^+$CD25^{hi} CD127^{low} cells, and the proportion of naive and memory Tregs was based on the expression of CD45RA (Figure 1A). Using a novel gating strategy based on CXCR3 and CCR6 expression on CCR4$^+$ cells, we evaluated the presence of these markers in naive and memory Tregs. The majority of naive cells were CCR4$^-$CXCR3$^-$CCR6$^-$ (Figure S1A). In contrast, the clear majority of memory Tregs were CCR4$^+$, with substantial but differential expression of CXCR3 and CCR6. The expression of these three chemokine receptors allowed the identification of four Th-like lineage subsets in circulation (Figure 1A). We then analyzed FoxP3 expression among these Th-like Treg subsets, and as expected, each subset had a higher frequency and median fluorescence intensity (MFI) than Teffs (Figures 1B and S1B). Furthermore, Th2-like Tregs exhibited the lowest FoxP3 MFI, whereas Th1/17-like Tregs expressed the highest (Figure S1C). Of note, the expression of CD25 and CCR4 did not follow the same pattern of expression as FoxP3 (Figures S1D and S1E). Following targeted RNA-sequencing (RNA-seq) on activated Th-like Treg subsets (in the absence of exogenous IL-2), principal-component analysis indicated Th2-like Tregs cluster separate from the other three Treg subpopulations, independent of donor variability (Figure 1C). Thus, for subsequent analysis, Th2-like Tregs were used as the comparator group. Differential gene expression analysis (Figure 1D) revealed an enrichment of corresponding Th-like genes in each subset and a combination of Th1 and Th17-related genes in Th1/17 Tregs (Figure 1E; Table S1). Gata-3, ROR$\gamma\tau$, and T-bet expression was then confirmed by protein expression (Figures 1F and S2A). Lastly, cytokine production was measured in supernatants of activated Th-like Tregs (Figure 1G). In line with the gene expression analysis, Th2-like Tregs produced significantly higher levels of IL-4, IL-5, and IL-13 than other Th-like subsets (Figure 1G). In addition to classical Th2-cytokines, Th2-like Tregs also produced more IL-2 than other subsets (Figure 1G). Higher production of IL-17A and IL-17F was observed in Th17-like Tregs, and higher production of interferon-γ (IFN-γ) was observed in Th1-like Tregs. Intermediate production of IL-17A and IFN-γ was observed in Th1/17 Tregs (Figure 1G). The production of cytokines was consistently and significantly lower in Th-like Tregs than in their Teff counterparts (Figure S2B). Thus, expression of CXCR3, CCR4, and CCR6 allowed us to define four Th-like Tregs in peripheral blood, which matched defining cytokines and transcription factors of their respective lineages.

Th2-like Tregs Exhibit the Highest Viability and Cytokine-Mediated Activation

FoxP3 has been shown to be a pro-apoptotic factor in developing Tregs in the absence of common gamma chain (γc)-dependent cytokine signals (Tai et al., 2013). Since Th2-like Tregs secreted higher levels of γc-dependent cytokine and exhibited the lowest FoxP3 MFI, we evaluated viability and cell activation after TCR engagement. After 3 days, viable, apoptotic, dead, and blasting cells were identified (Figure S3A). Th2-like Tregs showed the highest survival and blasting capacity (Figure 2A) as well as the lowest percentages of combined apoptotic and dead cells (Figure 2B). We next evaluated the effect of cytokines on the viability and blasting of Th-like Treg subsets, observing that IL-2 neutralization significantly reduced the blasting of Th2-like Tregs (Figure 2C), without affecting

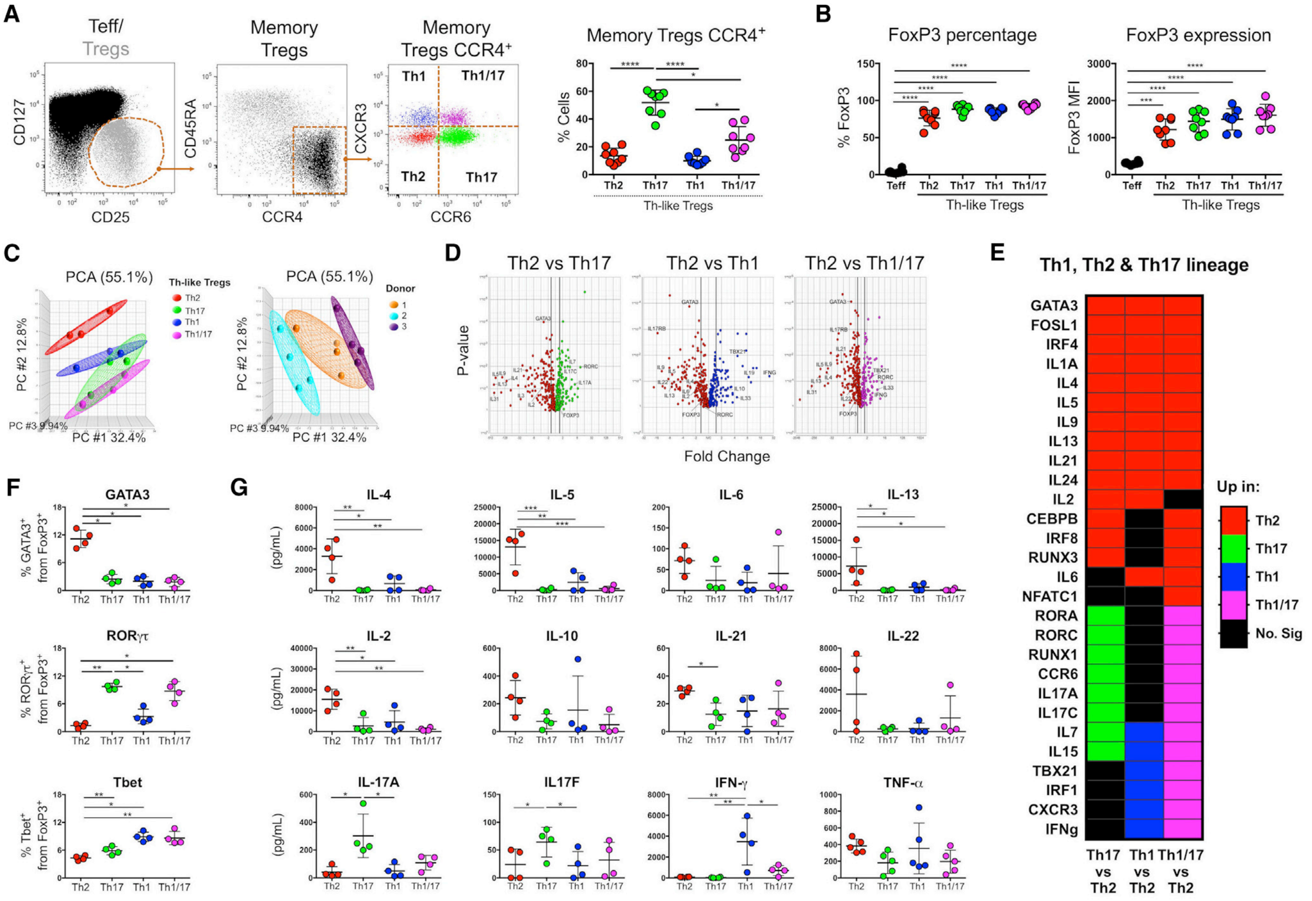

Figure 1. Identification of Four Th-like Tregs Based on CXCR3, CCR4, and CCR6 Expression

(A) CCR4, CXCR3, and CCR6 expression was analyzed in memory CD4^{+}CD25^{hi}CD127^{low}CD45RA^{-} Tregs. Four Th-like lineages were identified in the circulation: Th2, Th17, Th1, and Th1/17-like Tregs.

(B) FoxP3 expression between Teff and Th-like Treg subsets.

For (A) and (B), data are presented as mean ± SEM (n = 8) using independent values (RM one-way ANOVA with Tukey's test).

(C and D) Principal-component analysis (C) and volcano plots (D) showing ANOVA of RNA-seq data obtained from activated Th-like Treg subsets. Thick vertical lines indicate 1.5-fold change threshold (n = 3, using independent values clustered with ellipsoids).

(E) Heatmap showing upregulation of Th-lineage genes between Th-like Treg subsets using Partek software.

(F and G) Protein expression of GATA3, RORγτ, and T-bet in FoxP3^{+} Treg subsets (F) and absolute values of cytokine production by activated Th-like Treg subsets (G) (n = 4, mean ± SEM using independent values, RM one-way ANOVA with Tukey's test).

For all statistical tests, ****p < 0.0001, ***p < 0.001, **p < 0.01, and *p < 0.05 were considered significant.

viability, suggesting that autocrine IL-2 production contributes to higher activation of Th2-like Tregs. Addition of exogenous IL-2 rescued the blasting capacity of the other Treg subsets but did not increase cell survival (Figures 2D and S3B), which was mirrored in total memory Tregs (Figure S3C). IL-2 neutralization also reduced the blasting capacity of total memory Tregs, with no effects on viability (Figure S3D). To confirm this observation, we evaluated p53 expression and STAT5 signaling 16 hr post-activation in the presence or absence of exogenous IL-2. p53 expression was highest in Th1-like Tregs, and its expression was not affected by the addition of exogenous IL-2 in any subset (Figure 2E). Conversely, STAT5 phosphorylation was significantly increased in Th2-like Tregs compared to other subsets in the absence of IL-2, and addition of exogenous IL-2 rescued STAT5 phosphorylation in all Th-like Treg subsets (Figure 2E). These data were backed up by pathway analysis, as genes within JAK-STAT signaling pathways were significantly higher in Th2-like Tregs than in all other Th-like subsets (Figure 2F; Table S1). The TCR-signaling pathway was also evaluated, but no significant difference between Th-like Treg subsets was observed (Figure S4A), suggesting that higher viability was not due to differential TCR activation. Finally, we observed that Th2-like Tregs expressed an anti-apoptotic gene profile, whereas Th17, Th1, and Th1/17 Tregs expressed a more pro-apoptotic gene profile (Figure 2F; Table S1); thus, it is possible that other genes are regulating the higher viability in Th2-like Tregs. Overall, our data suggest that Th2-like Tregs have a survival advantage over other Treg subsets and a higher blasting capacity due to the autocrine IL-2/STAT5 signaling pathway.

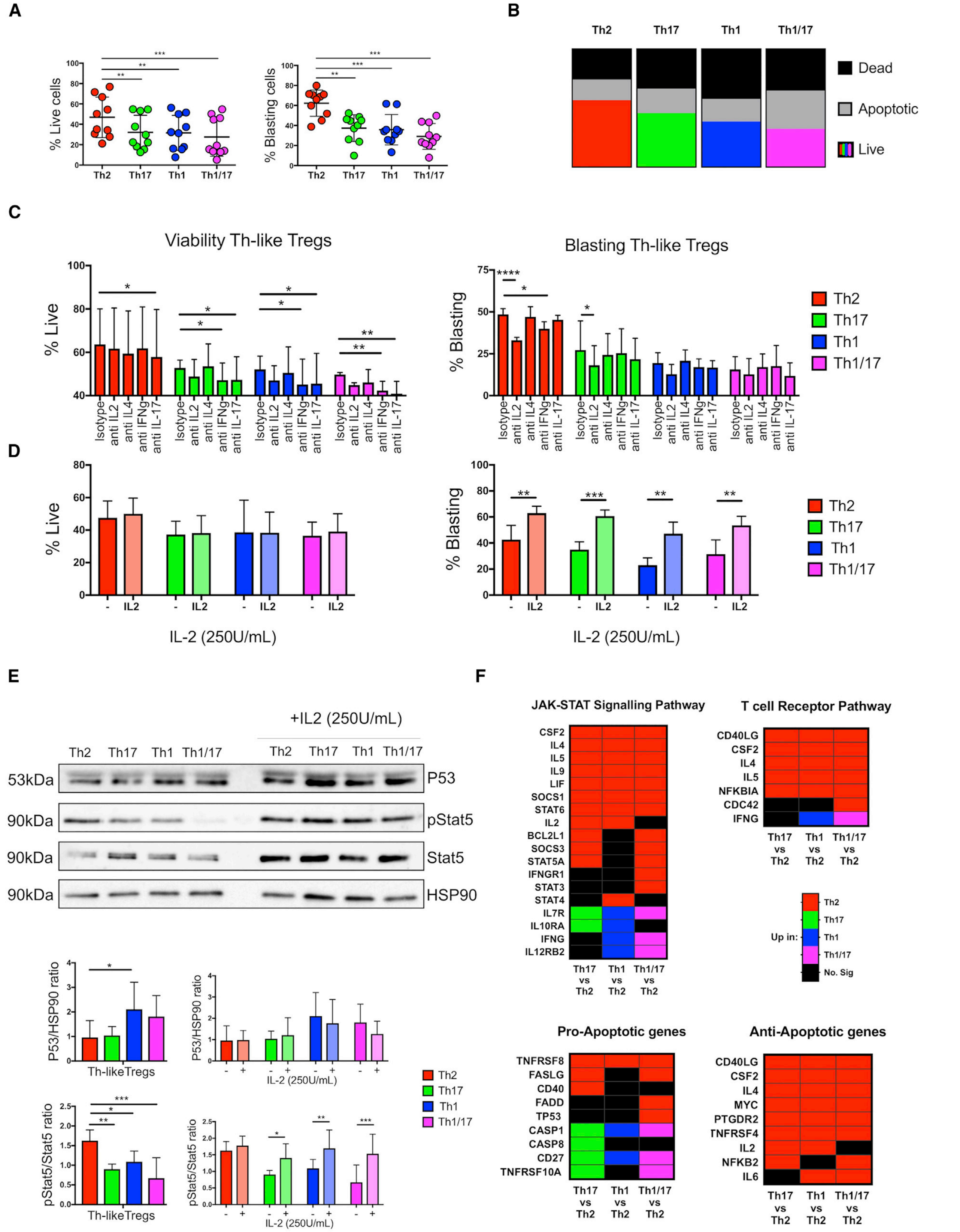

(legend on next page)

Th-like Treg Subsets Suppress Th-like Teffs, without Preferential Targeting of Their Teff Counterparts

Next, we investigated the capacity of Th-like Treg subsets to suppress their corresponding effector counterparts. Characterization of effector cells revealed that memory Teffs were mainly CD25^{int}, and unlike Tregs, they exhibited a substantial percentage of CCR4^{-} cells (Figure 3A). Proliferative ability and cytokine profile of Th-like Teff populations equivalent to the Th-like Treg subsets were analyzed (Figure 3A). After TCR activation, we observed higher proliferation of Th1/17 and Th17 compared to Th2 and Th1-like Teffs (Figure 3B). Cytokine production by Th-like Teff was, as expected, related to Th lineage and similar to the cytokine profiles obtained from Treg subsets (Figure S2B). Next, the ability of Th-like Tregs subsets to inhibit the proliferation of total effector or subpopulations was measured (Figure 3C). Results showed that Th-like Treg subsets reduced cell division of memory Teffs and Th-like Teffs, without preference for inhibition of their Teff counterparts (Figure 3D). Similarly, no inhibition by Treg subsets of lineage-specific cytokines produced by Teffs was observed (Figure 3E). Th-like Treg subsets suppressed pro-inflammatory cytokines, but they did not suppress IL-10, which was produced mainly by Th2-like Teffs (Figure 3E). Interestingly, Th2-like Tregs did not suppress proliferation of Th2-like Teffs as much as other Th-like Treg subsets, possibly due to higher expression of TIGIT, the only protein related to Treg function that is differentially expressed in Th2-like Tregs compared to other subsets after activation (Figure S4B). TIGIT is a co-inhibitory molecule that selectively inhibits pro-inflammatory responses of Th1 and Th17 cells, but not Th2 cells (Joller et al., 2014). Differences in the susceptibility to be suppressed between Teff subsets suggest that their distribution in the site of inflammation is also pertinent in understanding the regulation of the inflammatory response. Since our data showed that all Th-like Tregs suppress memory Teff, we then study the chemotaxis of Th-like Tregs to evaluate whether differences in their regulatory function in vivo may be mediated by differences in their migratory capacity.

Th2-like Tregs Exhibit Higher Chemotaxis to CCL17/22 Than Other Tregs and Their Counterpart Teffs

To characterize the migratory ability of the Th-like Tregs, the expression of chemokine receptors by each subtype and the genes associated to migration were evaluated (Figure 4A). Pathway analysis between Th-like Treg subsets revealed higher expression of genes associated with leukocyte trans-endothelial migration in Th2-like Tregs compared to other subsets (Figure 4B; Table S2), suggesting a higher migratory potential in this subset. In addition, we observed a differential expression of chemokines and chemokine receptors between Th-like Tregs (Figure 4B; Table S2). Having characterized the expression of chemokines and their receptors in the different Th-like Treg subsets, cell migration was then assessed using a trans-well system. We observed low migration of cells in the absence of chemokines and a preferential migration of CCR4^{+} cells to chemokines CCL17/22 (Figure 4C). We then evaluated the Th-like phenotype of migrated cells and observed that Th2-like Tregs migrated more than any of the other subsets to CCL17/22 and to a mixture of all the chemokines (Figure 4D). Th2-like Tregs also migrated even more than their Th2-like Teff counterpart in response to the same chemokines (Figure 4E). On the contrary, Th17 and Th1-like Tregs migrate less than their Teff counterparts in response to CCL20 and CXCL10, respectively (Figure 4E). Furthermore, Th2-like Tregs expressed more CCL17 than other Th-like subsets (Figure 4B; Table S2), suggesting that this subset not only migrates more but also has enhanced ability to recruit CCR4^{+} Tregs.

Th2-like Tregs Are More Prevalent in Tissues and Are the Main Infiltrating Subset Present in Melanoma and Colorectal Cancer

Th-like Treg subsets expressed distinctive chemokine signatures and exhibited different functional responses. Thus, we evaluated their distribution in primary and secondary lymphoid organs as well as peripheral tissues from healthy volunteers and patients with melanoma or colorectal cancer. We compared the expression of CCR4 between Th-like Tregs and Teff and their distribution in different tissues (Figure 5A) and peripheral blood (Figure 1A). We observed higher percentages of CCR4^{+} cells in Tregs than Teffs in all tissues; conversely, low expression of CCR4 was observed in the thymus (Figure S5A). Next, we dissected the distribution of Th-like Teffs/Tregs in different tissues (Figure 5B). High percentages of Th2-like cells were observed in the spleen, liver perfusate, and thymus, but thymic memory CD4^{+} T cells expressed very low levels of CCR4; therefore, the overall presence of Th-like cells in the thymus was low compared to other tissues. Th17-like Tregs were the main population in the skin, whereas the colon was enriched for Th1/17-like Tregs. In general, Th2-like Tregs were found preferentially in tissues compared to the circulation, even in the skin and colon, supporting the transmigration pathway previously observed (Figure 4B). When samples from patients with cancer (Tables 1 and S3) were analyzed, a higher Treg/Teff ratio was observed

Figure 2. Th2-like Tregs Exhibit Higher Viability, Activation, and JAK-STAT Signaling Pathway Than Other Treg Subsets

(A) Total percentages of live and blasting cells between Th-like Tregs 72 hr post-TCR activation in the absence of IL-2 (n = 10, mean ± SEM using independent values, RM one-way ANOVA with Tukey's test).

(B) Distribution of dead, apoptotic, and live cells between Th-like Tregs after TCR activation (n = 5).

(C and D) The percentage of live and blasting cells was analyzed in Th-like Treg subsets activated in the presence of neutralizing antibodies for IL-2, IL-4, IFN-γ, and IL-17 (all at 10 μg/mL) (C) or 250 U/ml exogenous IL-2 (D) (n = 4, mean ± SEM using bar charts, RM two-way ANOVA with Tukey's test).

(E) STAT5 signaling and p53 expression was measured in Th-like Tregs 16 hr post-TCR activation in the presence or absence of IL-2 (250 U/mL) (n = 4, mean ± SEM using bar charts, RM two-way ANOVA with Sidak's test).

(F) Heatmap showing upregulation of JAK-STAT, TCR signaling pathway and pro and anti-apoptotic genes between Th-like Treg subsets using Partek software and the KEGG database.

For all statistical tests, ****p < 0.0001, ***p < 0.001, **p < 0.01, and *p < 0.05 were considered significant.

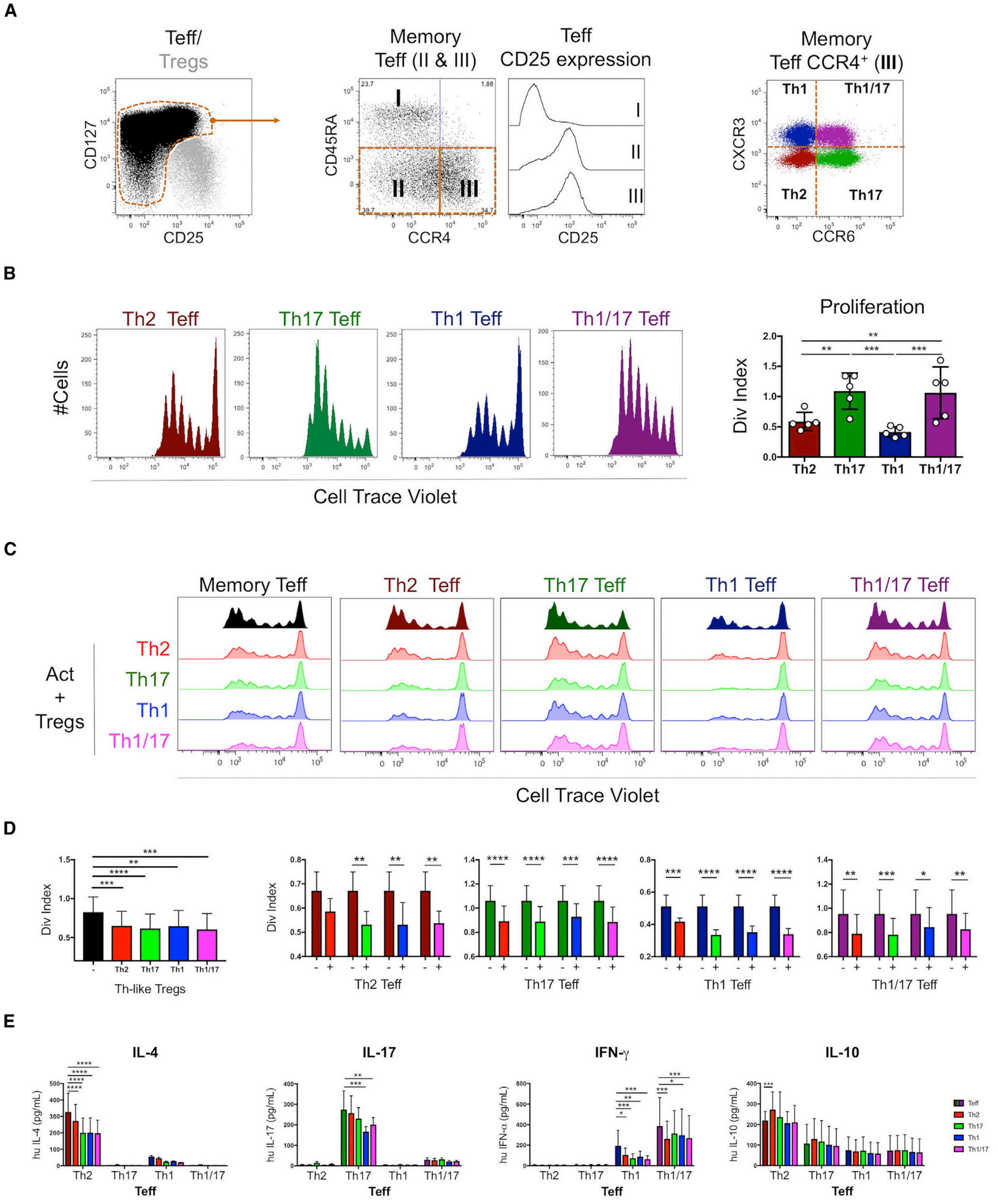

(legend on next page)

in malignant tissue than in healthy tissue (Figure 5C). Furthermore, we observed an increase of Th2-like Tregs and Teffs, concomitant with a reduction in Th1/17 lineages in tissues (Figure 5D) and peripheral blood (Figure S5B) from cancer patients. The production of Th2 cytokines was confirmed by intracellular staining in total CD4$^+$ T cells from malignant colon (Figure S5C). Interestingly, the increment of Th2-like cells was more prominent in Teffs than Tregs in patients with colorectal cancer, suggesting that an imbalance in favor of Th2 effector cells may contribute to cancer maintenance. The Th2 phenotype of colon samples from patients with colorectal cancer distant from the cancer area was similar to that obtained from healthy volunteers and significantly different from samples obtained from the cancer area (Figure 5E). Furthermore, our data were supported by the analysis of disease pathways (Figure 6A; Table S4) and previously published signatures from tumors infiltrating Tregs (Figure 6B) (De Simone et al., 2016; Plitas et al., 2016), revealing that Th2-like Treg genes were predominant in pathways associated with cancer. Furthermore, we observed high expression of CCR8 on the surface of resting Th2-like Tregs (Figure 6C), the main chemokine receptor found in Tregs isolated from tumor sites (De Simone et al., 2016; Plitas et al., 2016). Altogether, our phenotypic, genetic, and functional characterization of Th2-like Tregs suggests that this is the main Treg subset involved in cancer immunology.

DISCUSSION

Here, we provide a comprehensive transcriptomic analysis of circulating Th-like Tregs based on the expression of chemokine receptors, which allows cells to migrate into particular tissues in health and disease (Annunziato et al., 2007; Groom and Luster, 2011; Sugiyama et al., 2013; Yamazaki et al., 2008). Chemokine receptor CCR4 was expressed in all Th-like Treg subsets; however, Th2-like Tregs exhibited higher chemotaxis to CCL17/22 than to other Treg populations. Interestingly, the superior migratory capacity of Th2-like Tregs did not correlate with their CCR4 expression (MFI). However, transcriptome analysis revealed that Th2-like Tregs have higher expression of other genes involved in migration that may imbue them with a better migratory capacity. In addition, Th2-like Tregs express CCR8, which mediates migration to its ligand, CCL17, enhancing their migratory capacity (D'Ambrosio et al., 1998). Whole-genome microarray analysis revealed a selective upregulation of Th2 signature genes, including GATA3, IL4, IL5, and IL13, but a downregulation of IL2RA (CD25) and CCR4 upon downregulation of FoxP3 (Hansmann et al., 2012). This provides a possible explanation as to why Th2-like Tregs exhibited lower CCR4 MFI than other Tregs.

Cytokine production by Th1 and Th17-like Tregs was in line with previous reports (Duhen et al., 2012). Conversely, Th2-like Tregs produced the highest levels of IL-4, IL-5, and IL-13. Duhen et al. (2012), in their Th-like Treg characterization study, could not identify IL-4 or IL-22 by intracellular staining. We circumvented this technical problem by measuring secreted cytokines. Ectopic expression of Foxp3 in conventional T cells has been shown to repress cytokine production (Hori et al., 2003). However, instability of FoxP3 expression in Tregs allows for inflammatory Th cell phenotypes with the ability to secrete IFN-γ and IL-17 (Zhou et al., 2009), IL-4, IL-5, and IL-13 (Hansmann et al., 2012). This is in accordance with our results showing that the Treg population with the highest FoxP3 MFI was also the population with the lowest overall cytokine production. A key question regarding Treg biology is their stability and whether Tregs that express pro-inflammatory cytokine still maintain suppressive capacity. The co-expression of pro-inflammatory and anti-inflammatory cytokines by Th-like Tregs does not appear to have an impact on their suppressive capacity in our system and as previously reported (Duhen et al., 2012; Groom and Luster, 2011; Sugiyama et al., 2013; Yamazaki et al., 2008). It has been shown that Tregs expressing CCR6 are highly suppressive while still producing IL-17 (Duhen et al., 2012; Voo et al., 2009). Moreover, IL-17$^+$ Tregs from patients with inflamed intestinal mucosa were also shown to be functionally suppressive (Hovhannisyan et al., 2011; Valmori et al., 2010). All Th-like Tregs suppressed total memory Teffs; however, differences in their suppressive ability were revealed when Th-like Teffs were evaluated. For example, Th2-like Tregs did not reduce the proliferation of Th2 Teffs as significantly as they did with other Teffs. In addition, Th2-like Tregs exhibited higher expression of TIGIT compared to the other Treg subsets. This co-inhibitory molecule that has been shown to selectively inhibit pro-inflammatory Th1 and Th17 cell responses (Joller et al., 2014), supporting the presence of Th2 Teffs in cancer samples. We also observed lower proliferation rates in effector Th2 and Th1-like cells compared to Th17 and Th1/17 Teffs, suggesting that a cellular response meditated by Th17 and Th1/17 lineages could be more potent than a response meditate by Th1-like or Th2 Teffs. In fact, low susceptibility of Th17 and Th1/17 clones to the suppressive ability of total Tregs compare to Th1 and Th2 clones has been reported (Annunziato et al., 2007). This suggests that the presence of Th17 and Th1/17 lineages are favorable in tissues that require higher immune surveillance, whereas a Th2 lineage is favorable in malignant tissues, as they produce IL-10 and IL-4. The secretion of IL-4 is known to inhibit IFN-γ production, Th1 cell differentiation, and Th17 and Th1 responses (Wurtz et al., 2004). Besides Th2-type

Figure 3. Th-like Tregs Suppress Cell Division of Th-like Teffs without Preference for Lineage Counterparts

(A) Representative dot plots of Th-like Teffs. Th2, Th17, Th1, and Th1/17 were identified from memory Teff CCR4$^+$ cells.

(B) Representative histograms and total percentages of divided Cell Trace Violet$^+$ Th-like Teff subsets (1×10^5) stimulated with anti-CD3/CD28 beads at a 40:1 (cell/bead) ratio for 4 days (n = 5, mean ± SEM using bar chart and independent values, RM one-way ANOVA with Tukey's multiple comparison test).

(C and D) Representative histograms (C) and division (Div.) index (D) were obtained from suppression assays between memory Teff or Th-like Teff and Th-like Treg subsets. Teffs (1×10^5) alone or with autologous Tregs (0.5×10^5) were activated with anti-CD3/CD28 beads at a 40:1 (cell/bead) ratio for 4 days. The data are presented as division index obtained from FlowJo analysis (n = 6, mean ± SEM using bar charts, RM two-way ANOVA with Tukey's test).

(E) Absolute values of IL-4, IFN-γ, IL-17, and IL-10 obtained from supernatants after 4 days of suppression assays (n = 6, mean ± SD using bars, RM Two-way ANOVA with Tukey's test).

For all statistical tests, ****$p < 0.0001$, ***$p < 0.001$, **$p < 0.01$, and *$p < 0.05$ were considered significant.

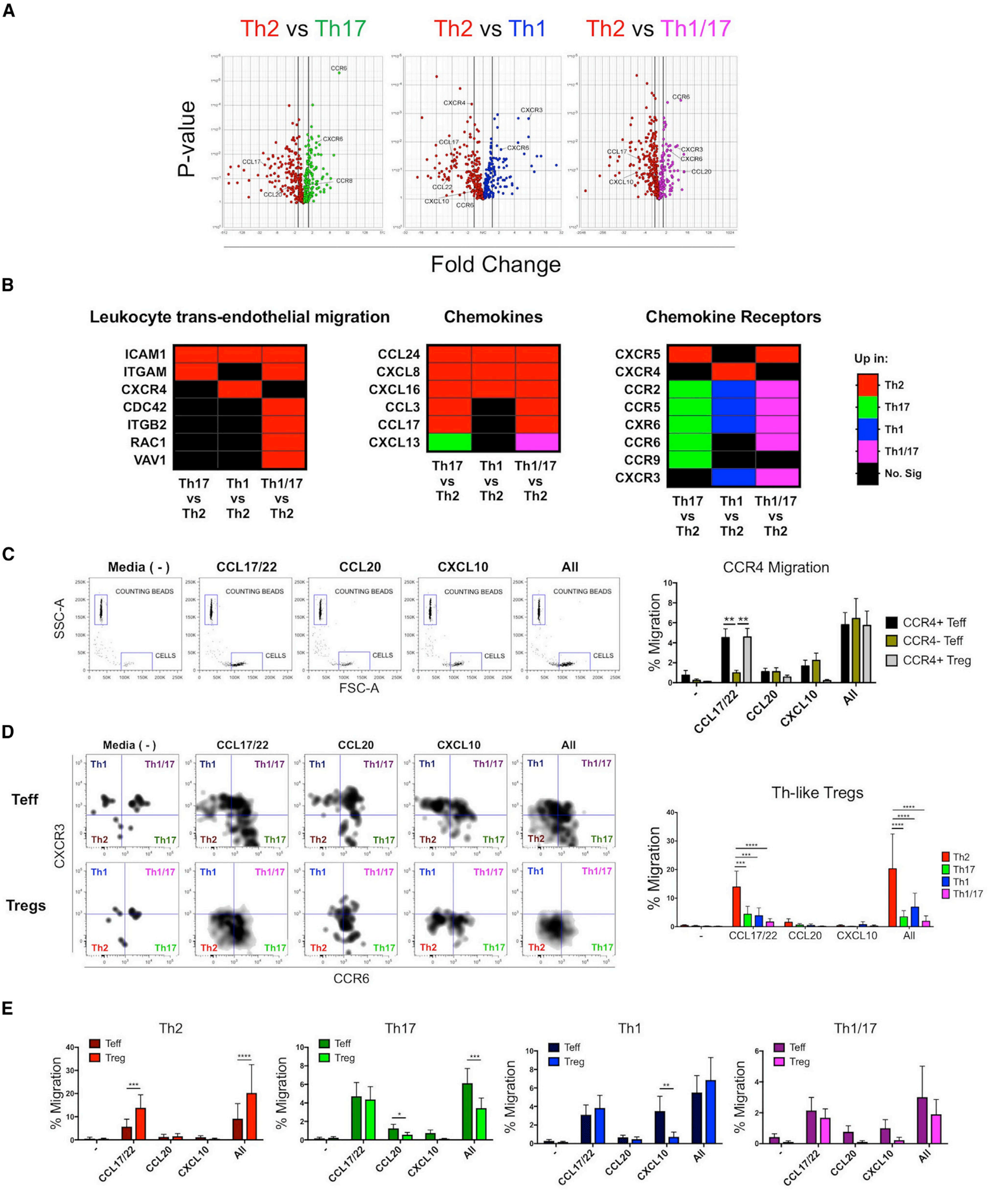

Figure 4. Th2-like Tregs Exhibit Higher Chemotaxis toward CCL17/22 Than Other Th-like Tregs and Th2-like Teffs

(A and B) Volcano plots showing RNA-seq data obtained from activated Th-like Treg subsets (A), and heatmaps showing upregulation of leukocyte trans-endothelial migration, chemokines, and chemokine receptors genes between Th-like Treg subsets using Partek software and the KEGG database (B).

(legend continued on next page)

cytokines, Th2-like Tregs also produced higher levels of IL-2 than other Th-like Treg subsets, but at much lower levels than Teffs. FoxP3 expression has been shown to induce cellular apoptosis and promote cell death in thymic Tregs in the absence of common γc-dependent cytokine signals, especially IL-2 (Tai et al., 2013). IL-4 can also improve proliferation due to a degree of redundancy in the ability of γc-cytokines to maintain functional Tregs (Maerten et al., 2005; Thornton et al., 2010; Yates et al., 2007). Our results showed that despite the fact Th2-like Tregs secreted higher levels of IL-4 than IL-2, the latter was more important for cell activation in vitro, but not for survival, as addition of exogenous IL-2 or neutralization of this cytokine did not affect the percentage of live cells in Th2-like Tregs. Further studies are required to identify the mechanism driving the higher survival; however, one of the highest upregulated genes in Th2-like Tregs when compared with all other Th-like Treg subsets was PTGDR2 (CRTh2), which has been shown to prevent apoptosis under cytokine deprivation (Xue et al., 2009). Altogether, our results suggest that Th2-like Tregs could be more resistant in environments with low levels of IL-2, such as malignant tissues (Giuntoli et al., 2009; Mocellin et al., 2001). In addition to higher viability, Th2-like Tregs also exhibited a higher chemotactic ability than other Treg subsets in response to CCL17/22. A positive correlation between the levels of CCL17 or CCL22 produced by tumor-associated monocytes and the frequency of FoxP3 Tregs in gastric cancer has previously been reported (Mizukami et al., 2008). The migration induced by CCL17 or CCL22 was significantly higher in $CD4^{+}CD25^{+}$ cells than in $CD4^{+}CD25^{-}$ cells (Mizukami et al., 2008), similar to our migration results. In addition, CCL22 has been shown to divert Tregs and control the growth of melanoma (Klarquist et al., 2016). More recently, poor prognosis in patients with metastatic melanoma due to Th2 polarization has been reported (Enninga et al., 2016). Together, these findings suggest that CCL22 contributes to tumor immunity by recruiting Tregs and Th2 cells. In colorectal cancer, intestinal epithelial cells have the capacity to regulate mucosal T cell trafficking through the release of CCL22 under inflammatory conditions. This allows them to modify the local mucosal cytokine milieu through recruitment of $CCR4^{+}$ T cells that counterbalance the inflammation with the specific production of Th2 cytokines (Berin et al., 2001). In addition, GATA3 is not essential for Treg survival under homeostatic conditions in mice, but GATA3-deficient Tregs do not accumulate at inflamed sites, especially in the gastrointestinal tract compared to other compartments (Wohlfert et al., 2011). Moreover, GATA3-deficient Tregs were not able to prevent colitis in a model of T cell transfer colitis (Wohlfert et al., 2011). Thus, migration of Th2 cells, both Teffs and Tregs, seems to be a mechanism by which the colon maintains gut homeostasis and controls inflammation. This anti-inflammatory response seems to be exacerbated by the tumor to maintain an anti-inflammatory environment, preventing anti-tumor responses and supporting tumor growth. In support of this, two independent studies recently demonstrated higher expression of CCR8 and OX40 in tumor-infiltrating Tregs from breast and lung cancer, colorectal adenocarcinoma, and melanoma (De Simone et al., 2016; Plitas et al., 2016). Previously, CCR8-expressing $CD4^{+}$ T cells have been shown to produce more Th2-type cytokines, such as IL-4, IL-5, IL-9, and IL-13, and less IFN-γ and IL-17 than $CCR8^{-}CD4^{+}$T cells (Soler et al., 2006). In addition, the OX40-OX40L pathway is required for Th2 responses (van Wanrooij et al., 2007). Furthermore, most genes upregulated in the Treg signature in cancer were also upregulated in Th2-like Tregs.

Overall, our data suggest that in malignant tissues with increased CCL17/22 secretion, Th2-like Tregs are preferentially attracted to tumor sites, where they display a survival advantage and the ability to inhibit Th1-Th17-Th1/17 effector lineages. Effector Th2 cells also migrate and play a suppressive role, as they secrete IL-10 and IL-4. The data presented here provide further support for studying tumor microenvironments to identify key cellular players maintaining the tumorigenic milieu and possible novel drug targets for tumor immunotherapy.

EXPERIMENTAL PROCEDURES

Phenotypic Analysis of Cell Subsets from Peripheral Blood and Tissues

Peripheral blood was obtained from healthy volunteers (age range, 22–36 years; male to female ratio, 3:5) after informed consent was approved. Patients with colorectal cancer (London-Dulwich Research Ethics Committee, reference number 15/LO/1998) and melanoma (King's College London and St Thomas' NHS Trust Ethics Committee, reference numbers 08/H0804/139 and 16/LO/0366) were consented in accordance with the Declaration of Helsinki. PBMCs were isolated by density-gradient centrifugation. Isolation protocols of mononuclear cells and research ethics for each tissue are described in Supplemental Experimental Procedures. Patient data are described briefly in Table 1, and more in details can be found in Table S3. The list of all reagents used in this study can be found in Supplemental Experimental Procedures.

Teff and Treg Isolation from Leukapheresis Blood Cones

RosetteSep Human $CD4^{+}$T Cell Enrichment Cocktail was used to isolate $CD4^{+}$ T cells from leukapheresis cone blood (NHS Blood and Transplant, Colindale, London, UK) obtained from anonymous healthy donors. After negative isolation of $CD4^{+}$ T cells, CD25 MicroBeads II were used to separate Tregs from Teffs. Tregs and Teffs were then sorted on a BD FACSAria I.

Flow Cytometry

PBMCs and mononuclear cells obtained from tissues were stained with anti-CD4, CD25, CD127, CXCR3, CCR4, CCR6, CD45RA, CD3, CD8, and CCR8 for 30 min at 4°C in the dark. Transcription factor staining was then performed with the Foxp3/Transcription Factor Staining Buffer Set using anti-FoxP3, GATA3, T-bet, and ROR$\gamma\tau$ for 30 min at 4°C in the dark. Samples were acquired on LSR Fortessa and files analyzed using FlowJo

(C) Representative dot plots and percentage of migrated memory Teffs and Tregs. Memory Teffs (5×10^{4}) and memory Tregs (5×10^{4}) were placed in the top chamber of a 5-μm-pore Transwell filter system with ICAM (1 μg/mL). Bottom chambers were filled with media only; CCL17/22 (0.5 μg/mL), CCL20 (0.5 μg/mL), or CXCL10 (0.5 μg/mL); or a combination of all of them. The percentage of migration for each subset was calculated as (number of cells in the bottom chamber after 1 hr × 100)/initial number of cells in the top chamber.

(D and E) Representative dot plots and percentage of migration between Th-like Treg subsets (D) and between $CCR4^{+}$ Th-like Teff and Th-like Tregs (E) (n = 6, mean ± SEM using bar charts, RM two-way ANOVA with Sidak's test).

For all statistical tests, ****$p < 0.0001$, ***$p < 0.001$, **$p < 0.01$, and *$p < 0.05$ were considered significant.

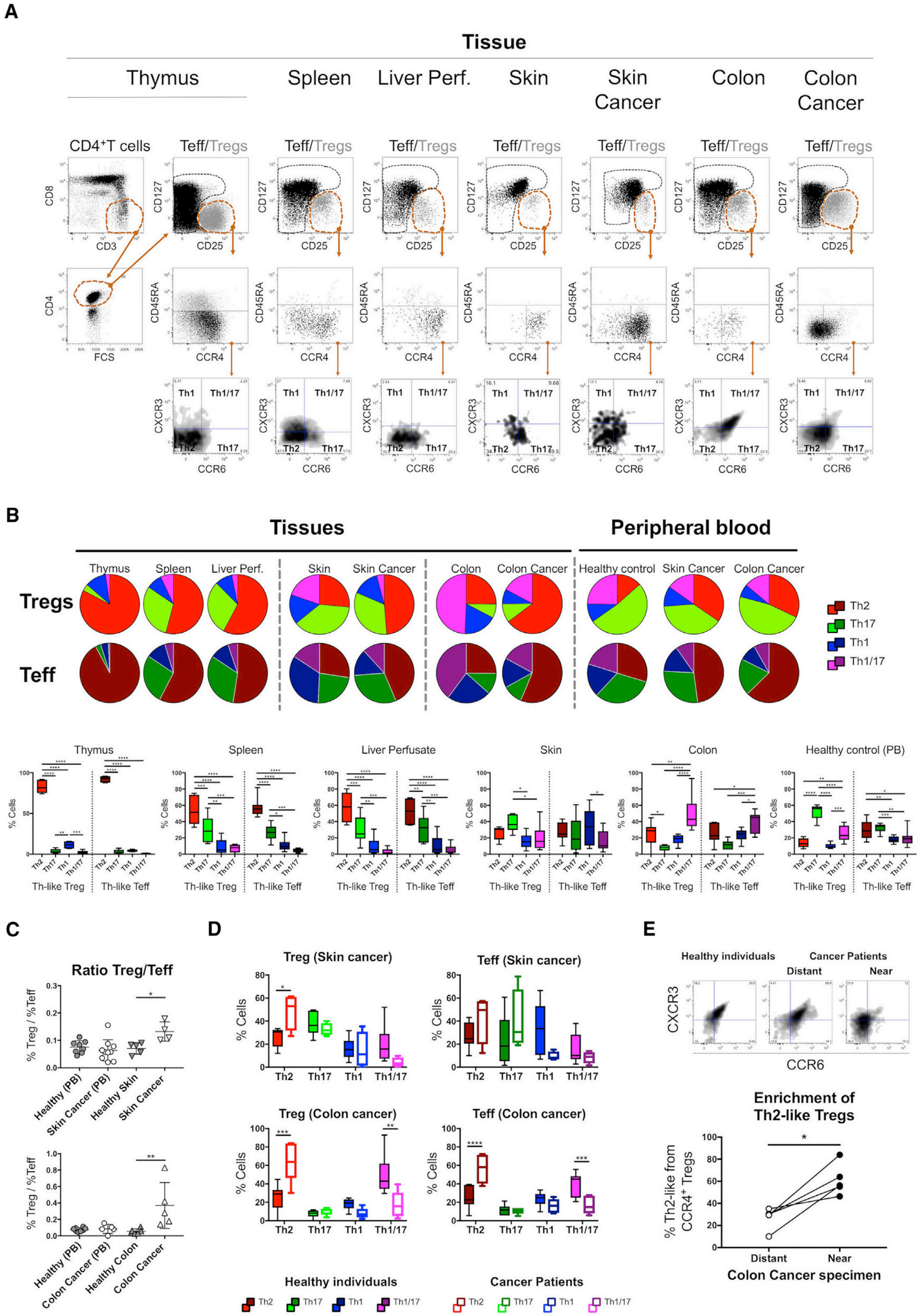

(legend on next page)

Table 1. Description of Cancer Patients

Patient Data	Melanoma	Colorectal Cancer
Number of patients	12	6
Male	6	4
Female	6	2
Age (y), mean (range)	61.8 (28–89)	62.3 (18–72)
Cancer stage		
I	0	2
IIIA	0	1
IIIC	1	2
IV	11	0
Not applicable	0	1

(Tree Star). Gates were set based on biological controls and fluorescence minus one controls.

RNA-Seq Targeted Panel

Fluorescence-activated cell sorting (FACS)-sorted Th-like Tregs (2×10^5) were activated with CD3/CD28 beads (ratio 1:4) for 72 hr. Cells were lysed in TRIzol, and RNA was isolated with Direct-Zol RNA MicroPrep w/Zymo-Spin columns. RNA-seq was performed using the QIAGEN Human Inflammation and Immunity Transcriptome RNA targeted panel. Samples were quantified with the Agilent High Sensitivity DNA Kit and sequenced with the Illumina MiSeq using MiSeq Reagent Kit v3 (150-cycle) (Illumina). Principal-component analysis, volcano plots, and pathway analysis were performed using QIAseq targeted RNA data analysis tools (QIAGEN) and Partek Genomics Suite software, version 6.6.

Viability, Blasting, and Cytokine Analysis

FACS-sorted Th-like Tregs ($0.5–1 \times 10^5$), total memory Tregs (0.5×10^5) and Teffs (1×10^5) were stimulated with anti-CD3/CD28 beads at a 4:1 (cell/bead) ratio in the absence or presence of the neutralizing antibodies anti-IL-2 (10 μg/mL, BioSource International), anti-IL-4, anti-IFN-γ, or anti-IL-17 (all 10 μg/mL, R&D Systems) or in the presence of different concentrations of exogenous IL-2 (Novartis). After 16 hr, STAT5 and p53 were evaluated using western blot. After 72 hr, viability and apoptosis were evaluated using the LIVE/DEAD Fixable Near-IR Dead Cell Stain Kit and Annexin V, and supernatants were used to detect human T cell cytokine production.

Suppression Assay

FACS-sorted Th-like Teff subpopulations were labeled with 5 μM Cell Trace Violet for 37°C for 15 min. 1×10^5 Teff subpopulations were plated alone and in co-culture with autologous Tregs at 1:2 (Treg/Teff) ratio. Cells were activated with anti-CD3/CD28 beads at a 40:1 (cell/bead) ratio. Cellular proliferation was assessed after 4 days by flow cytometry, and files were analyzed using FlowJo. The data are presented as division index (the total number of divisions divided by the number of cells that went into division) obtained from FlowJo analysis. Supernatant was used to detect human T cell cytokine production using BD Cytometric Bead Array.

Chemotaxis Assays

T cell migration was assessed using a 5-μm-pore Transwell filter system. The top chambers were incubated with ICAM (1 μg/mL) overnight at 37°C. Cell Trace Violet$^+$ memory Teffs and unstained memory Tregs were sorted and rested prior experiment. After resting, 5×10^4 Teffs + 5×10^4 Tregs in 50 μl X-VIVO15 serum-free medium were placed in the top chamber. The bottom chambers were filled with 100 μl X-VIVO15 serum-free only; with CCL17 + CCL22, CCL20, or CXCL10; or with a combination of all of them (all at 0.5 μg/mL, BioLegend). After 1 hr at 37°C, cells were harvested from bottom compartments, stained with anti-CXCR3, anti-CCR4 and anti-CCR6, counted using CountBright Absolute Counting Beads and analyzed by flow cytometry. The percentage of migration for each subset was calculated as (number of Th cells in the bottom chamber after 60 min × 100)/initial number of Th cells in the top chamber.

Statistical Analysis

Statistical tests were performed using Prism 7 software (GraphPad). Data are expressed as mean ± SD or SEM where applicable using individual values, bar charts, or boxplots. A repeated-measures (RM) two-way ANOVA was used to compare two related variables between Th-like subsets. An RM one-way ANOVA was used to compare one related variable between Th-like Tregs. An ordinary one-way ANOVA was used to compare CCR8 expression between Th-like Tregs. A two-tailed t test was used to compare tumor specimens. Post hoc tests were used as indicated in the figure legends. p values are reported as follows: *$p < 0.05$, **$p < 0.01$, ***$p < 0.001$, and ****$p < 0.0001$.

ACCESSION NUMBERS

The accession numbers for the Th-like Treg RNA-seq and flow cytometry data reported in this paper are GEO: GSE99733 and Flow Repository: FR-FCM-ZY75, respectively.

SUPPLEMENTAL INFORMATION

Supplemental Information includes Supplemental Experimental Procedures, five figures, and four tables and can be found with this article online at http://dx.doi.org/10.1016/j.celrep.2017.06.079.

AUTHOR CONTRIBUTIONS

Conceptualization, E.N.-L., L.H., and G.L.; Methodology, E.N.-L. and M.R.; Formal Analysis, E.N.-L. and R.M.; Investigation, E.N.-L., L.H., M.R., R.M., I.C., P.P., N.G., S.-J.H., M.Y., M.H., and O.H.; Resources, G.L., R.F.H., W.J., B.H., G.E.D., S.N.K., and N.P.; Writing – Original Draft, E.N.-L. and L.H.; Writing – Review & Editing, E.N.-L., L.H., M.R., R.M., I.C., P.P., N.P., R.I.L., and G.L.; Visualization, Supervision and Project Administration, E.N.-L.; Funding Acquisition, G.L.

ACKNOWLEDGMENTS

The authors thank all our donors and relatives for their participation in this study. The authors also thank Melanie Hussong and Gemma Gambril for their support with RNA-seq and analysis, Daniel Abu for his valuable help with colon

Figure 5. Distribution of Th-like Teff and Treg Subsets in Health and Malignancy

(A) Representative plots of chemokine receptor expression in Th-like Tregs obtained from tissues.

(B) Pie charts and total percentages of Th-like Tregs and Th-like Tregs Teff in tissues and peripheral blood (mean ± SEM using boxplots, RM two-way ANOVA with Sidak's test). Thymus = 6, spleen = 8, liver perfusates = 6, healthy skin = 5, skin with cancer = 4, healthy colon = 6, colon with cancer = 5, peripheral blood from healthy donors = 8, peripheral blood from patients with skin cancer = 10, and peripheral blood from patients with colon cancer = 5.

(C and D) Treg/Teff ratio (C) and tissue distribution of Th-like cells between healthy individuals and patients with skin or colon cancer (D). In (C), data are presented as mean ± SEM using individual values (one-way ANOVA with Dunnett's test). In (D), data are presented as mean ± SEM using boxplots (RM two-way ANOVA with Sidak's multiple comparison test).

(E) Representative plots and total percentages of Th2-like Tregs obtained from patients with colorectal cancer. Specimens obtained from tumor sites were compared with samples obtained from distant areas to the tumor (n = 4, independent values, two-tailed t test). *$p < 0.05$ was considered significant.

For all statistical tests, ****$p < 0.0001$, ***$p < 0.001$, **$p < 0.01$, and *$p < 0.05$ were considered significant.

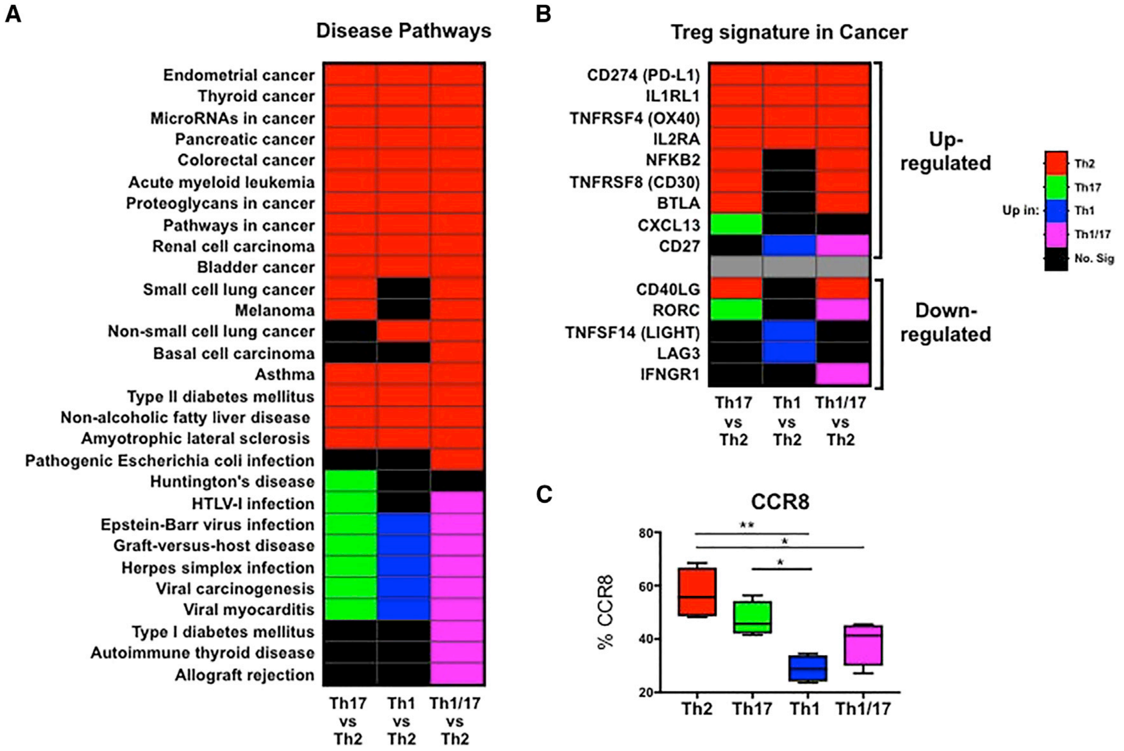

Figure 6. Disease Pathway Analysis of RNA-Seq Data Obtained from Th-like Treg Subsets

(A and B) Heatmap showing upregulation of Th-like Treg genes in disease pathways (A) and previously reported upregulated and downregulated genes by tumor infiltrating Tregs (De Simone et al., 2016; Plitas et al., 2016) using Partek software and KEGG database (B).

(C) CCR8 expression in Th-like Treg subsets (n = 5, boxplot using minimum to maximum, ordinary one-way ANOVA, with Holm-Sidak's test). $^{**}p < 0.01$ and $^{*}p < 0.05$ were considered significant.

resections, Dr. Graham Davies for providing thymuses, Dr. Sara Lombardi for recruitment of melanoma patient volunteers, Dr. Giovanni Povoleri for his suggestions for GSEA analysis, and Francisco Gonzalez for all his valuable support during this research project. We also thank the Flow Core and the Genomic Core of Guy's Hospital. E.N.-L. was funded by a scholarship from CONICYT Becas-Chile. This work was supported by the Department of Health via the National Institute for Health Research Comprehensive Biomedical Research Centre award to Guy's and St Thomas' NHS Foundation Trust in partnership with King's College London and King's College Hospital NHS Foundation Trust. This work was also supported by the British Heart Foundation (BHF; grant RG/13/12/30395), the European Union 7th Framework Programme (EU FP7), Cancer Research UK/NIHR in England and DoH in Scotland, the Wales and Northern Ireland Experimental Cancer Medicine Centre (C10355/A15587), and the Medical Research Council (MR/L023091/1).

Received: March 6, 2017
Revised: May 16, 2017
Accepted: June 26, 2017
Published: July 18, 2017

REFERENCES

Annunziato, F., Cosmi, L., Santarlasci, V., Maggi, L., Liotta, F., Mazzinghi, B., Parente, E., Filì, L., Ferri, S., Frosali, F., et al. (2007). Phenotypic and functional features of human Th17 cells. J. Exp. Med. *204*, 1849–1861.

Bailey, S.R., Nelson, M.H., Himes, R.A., Li, Z., Mehrotra, S., and Paulos, C.M. (2014). Th17 cells in cancer: the ultimate identity crisis. Front. Immunol. *5*, 276.

Berin, M.C., Dwinell, M.B., Eckmann, L., and Kagnoff, M.F. (2001). Production of MDC/CCL22 by human intestinal epithelial cells. Am. J. Physiol. Gastrointest. Liver Physiol. *280*, G1217–G1226.

D'Ambrosio, D., Iellem, A., Bonecchi, R., Mazzeo, D., Sozzani, S., Mantovani, A., and Sinigaglia, F. (1998). Selective up-regulation of chemokine receptors CCR4 and CCR8 upon activation of polarized human type 2 Th cells. J. Immunol. *161*, 5111–5115.

De Monte, L., Reni, M., Tassi, E., Clavenna, D., Papa, I., Recalde, H., Braga, M., Di Carlo, V., Doglioni, C., and Protti, M.P. (2011). Intratumor T helper type 2 cell infiltrate correlates with cancer-associated fibroblast thymic stromal lymphopoietin production and reduced survival in pancreatic cancer. J. Exp. Med. *208*, 469–478.

De Simone, M., Arrigoni, A., Rossetti, G., Gruarin, P., Ranzani, V., Politano, C., Bonnal, R.J.P., Provasi, E., Sarnicola, M.L., Panzeri, I., et al. (2016). Transcriptional landscape of human tissue lymphocytes unveils uniqueness of tumor-infiltrating T regulatory cells. Immunity *45*, 1135–1147.

Duhen, T., Duhen, R., Lanzavecchia, A., Sallusto, F., and Campbell, D.J. (2012). Functionally distinct subsets of human FOXP3+ Treg cells that phenotypically mirror effector Th cells. Blood *119*, 4430–4440.

Enninga, E.A.L., Nevala, W.K., Holtan, S.G., Leontovich, A.A., and Markovic, S.N. (2016). Galectin-9 modulates immunity by promoting Th2/M2 differentiation and impacts survival in patients with metastatic melanoma. Melanoma Res. *26*, 429–441.

Erhardt, A., Wegscheid, C., Claass, B., Carambia, A., Herkel, J., Mittrücker, H.-W., Panzer, U., and Tiegs, G. (2011). CXCR3 deficiency exacerbates liver disease and abrogates tolerance in a mouse model of immune-mediated hepatitis. J. Immunol. *186*, 5284–5293.

Gabitass, R.F., Annels, N.E., Stocken, D.D., Pandha, H.A., and Middleton, G.W. (2011). Elevated myeloid-derived suppressor cells in pancreatic, esophageal and gastric cancer are an independent prognostic factor and are associated with significant elevation of the Th2 cytokine interleukin-13. Cancer Immunol. Immunother. *60*, 1419–1430.

Giuntoli, R.L., 2nd, Webb, T.J., Zoso, A., Rogers, O., Diaz-Montes, T.P., Bristow, R.E., and Oelke, M. (2009). Ovarian cancer-associated ascites demonstrates altered immune environment: implications for antitumor immunity. Anticancer Res. *29*, 2875–2884.

Gobert, M., Treilleux, I., Bendriss-Vermare, N., Bachelot, T., Goddard-Leon, S., Arfi, V., Biota, C., Doffin, A.C., Durand, I., Olive, D., et al. (2009). Regulatory T cells recruited through CCL22/CCR4 are selectively activated in lymphoid infiltrates surrounding primary breast tumors and lead to an adverse clinical outcome. Cancer Res. *69*, 2000–2009.

Groom, J.R., and Luster, A.D. (2011). CXCR3 in T cell function. Exp. Cell Res. *317*, 620–631.

Guenova, E., Watanabe, R., Teague, J.E., Desimone, J.A., Jiang, Y., Dowlatshahi, M., Schlapbach, C., Schaekel, K., Rook, A.H., Tawa, M., et al. (2013). TH2 cytokines from malignant cells suppress TH1 responses and enforce a global TH2 bias in leukemic cutaneous T-cell lymphoma. Clin. Cancer Res. *19*, 3755–3763.

Hansmann, L., Schmidl, C., Kett, J., Steger, L., Andreesen, R., Hoffmann, P., Rehli, M., and Edinger, M. (2012). Dominant Th2 differentiation of human regulatory T cells upon loss of FOXP3 expression. J. Immunol. *188*, 1275–1282.

Hartigan-O'Connor, D.J., Poon, C., Sinclair, E., and McCune, J.M. (2007). Human CD4+ regulatory T cells express lower levels of the IL-7 receptor alpha chain (CD127), allowing consistent identification and sorting of live cells. J. Immunol. Methods *319*, 41–52.

Hori, S., Nomura, T., and Sakaguchi, S. (2003). Control of regulatory T cell development by the transcription factor Foxp3. Science *299*, 1057–1061.

Hou, N., Zhang, X., Zhao, L., Zhao, X., Li, Z., Song, T., and Huang, C. (2013). A novel chronic stress-induced shift in the Th1 to Th2 response promotes colon cancer growth. Biochem. Biophys. Res. Commun. *439*, 471–476.

Hovhannisyan, Z., Treatman, J., Littman, D.R., and Mayer, L. (2011). Characterization of interleukin-17-producing regulatory T cells in inflamed intestinal mucosa from patients with inflammatory bowel diseases. Gastroenterology *140*, 957–965.

Joller, N., Lozano, E., Burkett, P.R., Patel, B., Xiao, S., Zhu, C., Xia, J., Tan, T.G., Sefik, E., Yajnik, V., et al. (2014). Treg cells expressing the coinhibitory molecule TIGIT selectively inhibit proinflammatory Th1 and Th17 cell responses. Immunity *40*, 569–581.

Klarquist, J., Tobin, K., Farhangi Oskuei, P., Henning, S.W., Fernandez, M.F., Dellacecca, E.R., Navarro, F.C., Eby, J.M., Chatterjee, S., Mehrotra, S., et al. (2016). Ccl22 diverts T regulatory cells and controls the growth of melanoma. Cancer Res. *76*, 6230–6240.

Liu, W., Putnam, A.L., Xu-Yu, Z., Szot, G.L., Lee, M.R., Zhu, S., Gottlieb, P.A., Kapranov, P., Gingeras, T.R., Fazekas de St Groth, B., et al. (2006). CD127 expression inversely correlates with FoxP3 and suppressive function of human CD4+ T reg cells. J. Exp. Med. *203*, 1701–1711.

Lutgendorf, S.K., Lamkin, D.M., DeGeest, K., Anderson, B., Dao, M., McGinn, S., Zimmerman, B., Maiseri, H., Sood, A.K., and Lubaroff, D.M. (2008). Depressed and anxious mood and T-cell cytokine expressing populations in ovarian cancer patients. Brain Behav. Immun. *22*, 890–900.

Ma, C., and Dong, X. (2011). Colorectal cancer-derived Foxp3(+) IL-17(+) T cells suppress tumour-specific CD8+ T cells. Scand. J. Immunol. *74*, 47–51.

Maerten, P., Shen, C., Bullens, D.M.A., Van Assche, G., Van Gool, S., Geboes, K., Rutgeerts, P., and Ceuppens, J.L. (2005). Effects of interleukin 4 on CD25+CD4+ regulatory T cell function. J. Autoimmun. *25*, 112–120.

Miyara, M., Yoshioka, Y., Kitoh, A., Shima, T., Wing, K., Niwa, A., Parizot, C., Taflin, C., Heike, T., Valeyre, D., et al. (2009). Functional delineation and differentiation dynamics of human CD4+ T cells expressing the FoxP3 transcription factor. Immunity *30*, 899–911.

Mizukami, Y., Kono, K., Kawaguchi, Y., Akaike, H., Kamimura, K., Sugai, H., and Fujii, H. (2008). CCL17 and CCL22 chemokines within tumor microenvironment are related to accumulation of Foxp3+ regulatory T cells in gastric cancer. Int. J. Cancer *122*, 2286–2293.

Mocellin, S., Wang, E., and Marincola, F.M. (2001). Cytokines and immune response in the tumor microenvironment. J. Immunother. *24*, 392–407.

Ochi, A., Nguyen, A.H., Bedrosian, A.S., Mushlin, H.M., Zarbakhsh, S., Barilla, R., Zambirinis, C.P., Fallon, N.C., Rehman, A., Pylayeva-Gupta, Y., et al. (2012). MyD88 inhibition amplifies dendritic cell capacity to promote pancreatic carcinogenesis via Th2 cells. J. Exp. Med. *209*, 1671–1687.

Oo, Y.H., Weston, C.J., Lalor, P.F., Curbishley, S.M., Withers, D.R., Reynolds, G.M., Shetty, S., Harki, J., Shaw, J.C., Eksteen, B., et al. (2010). Distinct roles for CCR4 and CXCR3 in the recruitment and positioning of regulatory T cells in the inflamed human liver. J. Immunol. *184*, 2886–2898.

Pagès, F., Berger, A., Camus, M., Sanchez-Cabo, F., Costes, A., Molidor, R., Mlecnik, B., Kirilovsky, A., Nilsson, M., Damotte, D., et al. (2005). Effector memory T cells, early metastasis, and survival in colorectal cancer. N. Engl. J. Med. *353*, 2654–2666.

Pernot, S., Terme, M., Voron, T., Colussi, O., Marcheteau, E., Tartour, E., and Taieb, J. (2014). Colorectal cancer and immunity: what we know and perspectives. World J. Gastroenterol. *20*, 3738–3750.

Perros, F., Hoogsteden, H.C., Coyle, A.J., Lambrecht, B.N., and Hammad, H. (2009). Blockade of CCR4 in a humanized model of asthma reveals a critical role for DC-derived CCL17 and CCL22 in attracting Th2 cells and inducing airway inflammation. Allergy *64*, 995–1002.

Plitas, G., Konopacki, C., Wu, K., Bos, P.D., Morrow, M., Putintseva, E.V., Chudakov, D.M., and Rudensky, A.Y. (2016). Regulatory T cells exhibit distinct features in human breast cancer. Immunity *45*, 1122–1134.

Povoleri, G.A.M., Scottà, C., Nova-Lamperti, E.A., John, S., Lombardi, G., and Afzali, B. (2013). Thymic versus induced regulatory T cells - who regulates the regulators? Front. Immunol. *4*, 169.

Rosenblum, M.D., Way, S.S., and Abbas, A.K. (2016). Regulatory T cell memory. Nat. Rev. Immunol. *16*, 90–101.

Sakaguchi, S., Yamaguchi, T., Nomura, T., and Ono, M. (2008). Regulatory T cells and immune tolerance. Cell *133*, 775–787.

Soler, D., Chapman, T.R., Poisson, L.R., Wang, L., Cote-Sierra, J., Ryan, M., McDonald, A., Badola, S., Fedyk, E., Coyle, A.J., et al. (2006). CCR8 expression identifies CD4 memory T cells enriched for FOXP3+ regulatory and Th2 effector lymphocytes. J. Immunol. *177*, 6940–6951.

Sugiyama, D., Nishikawa, H., Maeda, Y., Nishioka, M., Tanemura, A., Katayama, I., Ezoe, S., Kanakura, Y., Sato, E., Fukumori, Y., et al. (2013). Anti-CCR4 mAb selectively depletes effector-type FoxP3+CD4+ regulatory T cells, evoking antitumor immune responses in humans. Proc. Natl. Acad. Sci. USA *110*, 17945–17950.

Tai, X., Erman, B., Alag, A., Mu, J., Kimura, M., Katz, G., Guinter, T., McCaughtry, T., Etzensperger, R., Feigenbaum, L., et al. (2013). Foxp3 transcription factor is proapoptotic and lethal to developing regulatory T cells unless counterbalanced by cytokine survival signals. Immunity *38*, 1116–1128.

Thornton, A.M., Korty, P.E., Tran, D.Q., Wohlfert, E.A., Murray, P.E., Belkaid, Y., and Shevach, E.M. (2010). Expression of Helios, an Ikaros transcription factor family member, differentiates thymic-derived from peripherally induced Foxp3+ T regulatory cells. J. Immunol. *184*, 3433–3441.

Valmori, D., Raffin, C., Raimbaud, I., and Ayyoub, M. (2010). Human RORγt+ TH17 cells preferentially differentiate from naive FOXP3+Treg in the presence of lineage-specific polarizing factors. Proc. Natl. Acad. Sci. USA *107*, 19402–19407.

van Wanrooij, E.J.A., van Puijvelde, G.H.M., de Vos, P., Yagita, H., van Berkel, T.J.C., and Kuiper, J. (2007). Interruption of the Tnfrsf4/Tnfsf4 (OX40/OX40L) pathway attenuates atherogenesis in low-density lipoprotein receptor-deficient mice. Arterioscler. Thromb. Vasc. Biol. *27*, 204–210.

Voo, K.S., Wang, Y.-H., Santori, F.R., Boggiano, C., Wang, Y.-H., Arima, K., Bover, L., Hanabuchi, S., Khalili, J., Marinova, E., et al. (2009). Identification

of IL-17-producing FOXP3+ regulatory T cells in humans. Proc. Natl. Acad. Sci. USA *106*, 4793–4798.

Wohlfert, E.A., Grainger, J.R., Bouladoux, N., Konkel, J.E., Oldenhove, G., Ribeiro, C.H., Hall, J.A., Yagi, R., Naik, S., Bhairavabhotla, R., et al. (2011). GATA3 controls Foxp3^{+} regulatory T cell fate during inflammation in mice. J. Clin. Invest. *121*, 4503–4515.

Wong, M.T., Ong, D.E.H., Lim, F.S.H., Teng, K.W.W., McGovern, N., Narayanan, S., Ho, W.Q., Cerny, D., Tan, H.K.K., Anicete, R., et al. (2016). A high-dimensional atlas of human T cell diversity reveals tissue-specific trafficking and cytokine signatures. Immunity *45*, 442–456.

Wurtz, O., Bajénoff, M., and Guerder, S. (2004). IL-4-mediated inhibition of IFN-gamma production by CD4+ T cells proceeds by several developmentally regulated mechanisms. Int. Immunol. *16*, 501–508.

Xue, L., Barrow, A., and Pettipher, R. (2009). Novel function of CRTH2 in preventing apoptosis of human Th2 cells through activation of the phosphatidylinositol 3-kinase pathway. J. Immunol. *182*, 7580–7586.

Yamazaki, T., Yang, X.O., Chung, Y., Fukunaga, A., Nurieva, R., Pappu, B., Martin-Orozco, N., Kang, H.S., Ma, L., Panopoulos, A.D., et al. (2008). CCR6 regulates the migration of inflammatory and regulatory T cells. J. Immunol. *181*, 8391–8401.

Yang, S., Wang, B., Guan, C., Wu, B., Cai, C., Wang, M., Zhang, B., Liu, T., and Yang, P. (2011). Foxp3+IL-17+ T cells promote development of cancer-initiating cells in colorectal cancer. J. Leukoc. Biol. *89*, 85–91.

Yates, J., Rovis, F., Mitchell, P., Afzali, B., Tsang, J., Garin, M., Lechler, R.I., Lombardi, G., and Garden, O.A. (2007). The maintenance of human CD4+ CD25+ regulatory T cell function: IL-2, IL-4, IL-7 and IL-15 preserve optimal suppressive potency in vitro. Int. Immunol. *19*, 785–799.

Zhou, X., Bailey-Bucktrout, S.L., Jeker, L.T., Penaranda, C., Martínez-Llordella, M., Ashby, M., Nakayama, M., Rosenthal, W., and Bluestone, J.A. (2009). Instability of the transcription factor Foxp3 leads to the generation of pathogenic memory T cells in vivo. Nat. Immunol. *10*, 1000–1007.

Zhu, X.-W., Zhu, H.-Z., Zhu, Y.-Q., Feng, M.-H., Qi, J., and Chen, Z.-F. (2016). Foxp3 expression in CD4(+)CD25(+)Foxp3(+) regulatory T cells promotes development of colorectal cancer by inhibiting tumor immunity. J. Huazhong Univ. Sci. Technolog. Med. Sci. *36*, 677–682.

Ziegler, S.F. (2007). FOXP3: not just for regulatory T cells anymore. Eur. J. Immunol. *37*, 21–23.

Cell Reports, Volume *20*

Supplemental Information

An Atlas of Human Regulatory T Helper-like Cells Reveals Features of Th2-like Tregs that Support a Tumorigenic Environment

Leena Halim, Marco Romano, Reuben McGregor, Isabel Correa, Polychronis Pavlidis, Nathali Grageda, Sec-Julie Hoong, Muhammed Yuksel, Wayel Jassem, Rosalind F. Hannen, Mark Ong, Olivia Mckinney, Bu'Hussain Hayee, Sophia N. Karagiannis, Nicholas Powell, Robert I. Lechler, Estefania Nova-Lamperti, and Giovanna Lombardi

Supplemental Material

Supplemental Figures

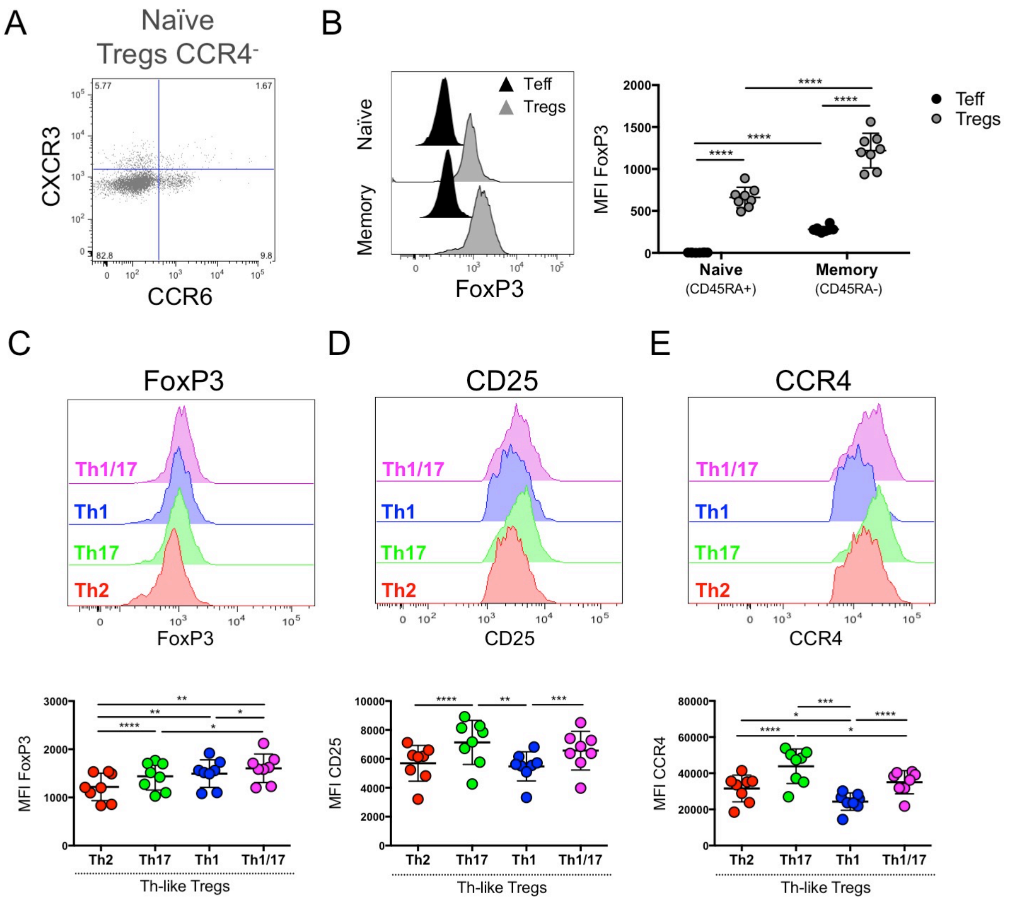

Figure S1: Comparison of FoxP3, CD25 and CCR4 expression between Th-like Teff and Treg subsets from healthy volunteers, related to Figure 1.

(A) Representative histograms of CCR4, CXCR3 and CCR6 expression analysed in naïve $CD45RA^{+}$ $CD4^{+}CD25^{hi}CD127^{low}$ Tregs. **(B)** Representative histograms and median fluorescence intensity (MFI) of FoxP3 expression in naïve and memory Teff and Tregs [n=8, mean ± SEM using independent values, RM Two-way ANOVA with Sidak's multiple comparison test]. **(C)** Representative histograms and MFI of FoxP3, **(C)** CD25 and **(D)** CCR4 between Th-like Tregs [n=8, mean ± SEM using independent values, RM One-way ANOVA with Tukey's multiple comparison test]. For all statistical tests **** *P*<0.0001, *** *P*<0.001, ** *P*<0.01 and * *P*<0.05 were considered significant.

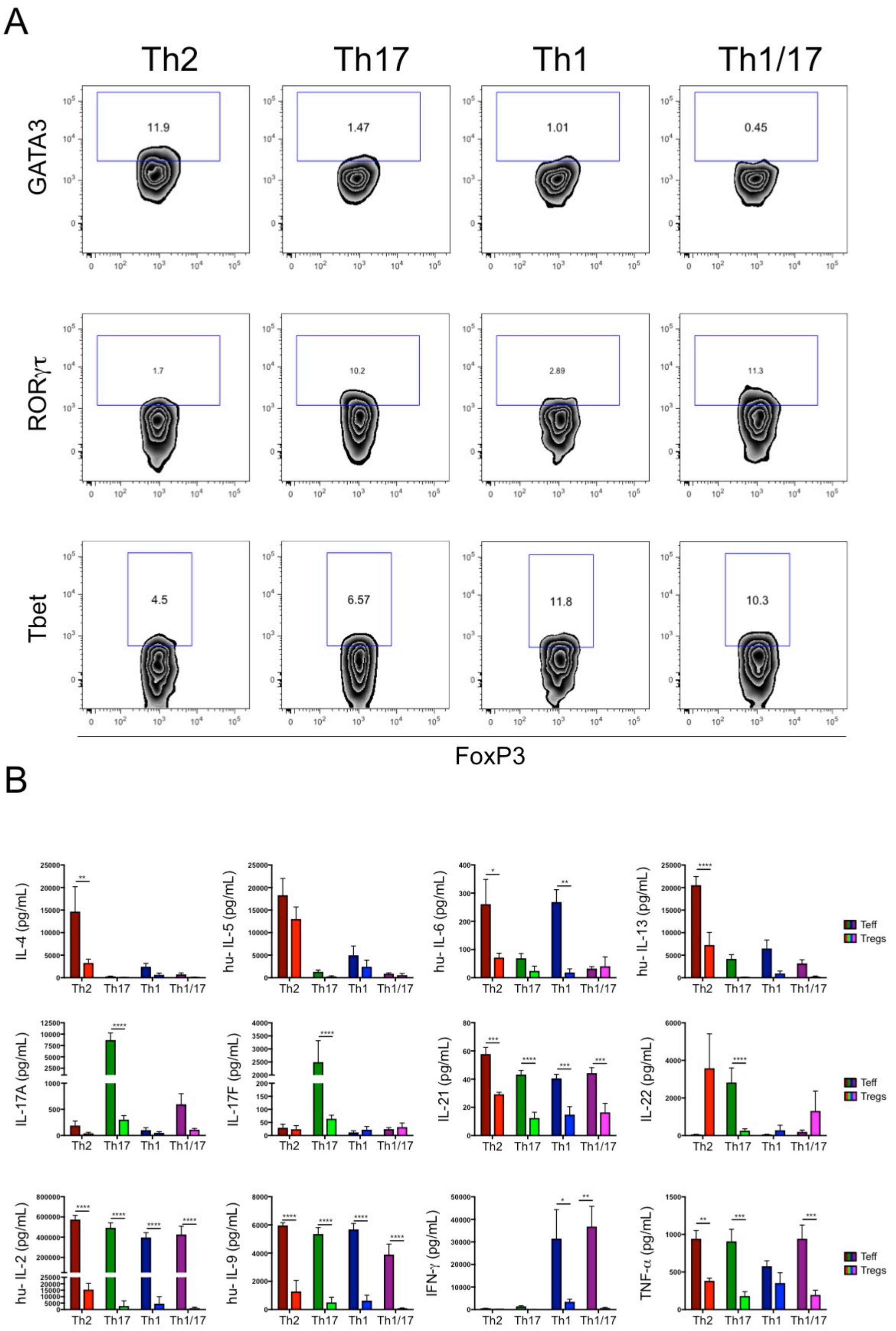

Figure S2: Transcription Factors expression by Th-like Treg subsets, related to Figure 1. (**A**) Representative contour plots of GATA-3, Tbet and RORγτ expression in resting FoxP3$^+$ Th-like Tregs. (**B**) Absolute values of cytokine production by activated Th-like Teff [■Th2, ■Th17, ■Th1 & ■ Th1/17] and Th-like Treg subsets [■Th2, ■Th17, ■Th1 & ■Th1/17] [n=5, mean ± SEM using bar charts, RM Two-way ANOVA with Sidak's test]. For all statistical tests **** *P*<0.0001, *** *P*<0.001, ** *P*<0.01 and * *P*<0.05 were considered significant.

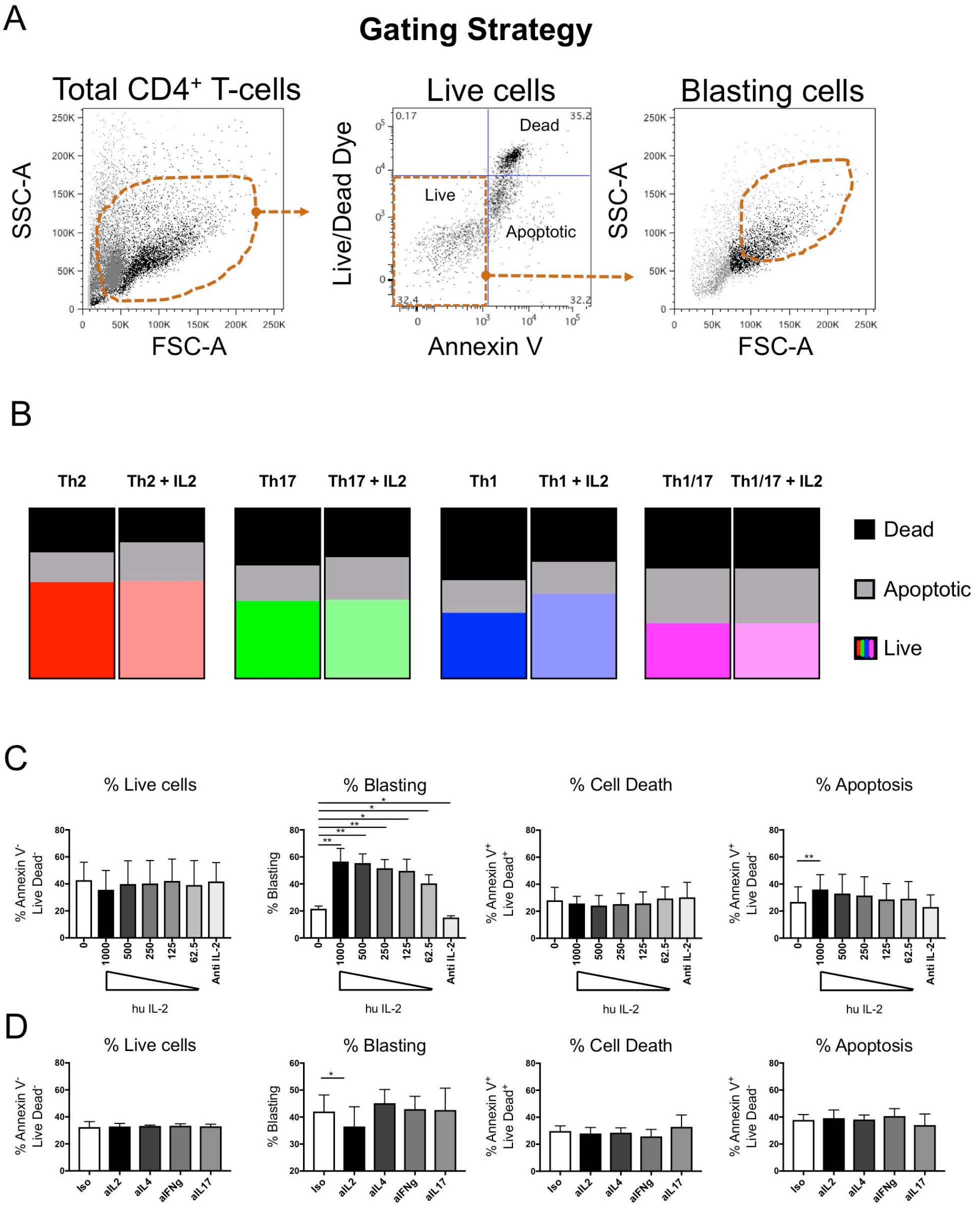

Figure S3: Cytokine effect in Th-like Treg viability and blasting, related to Figure 2.
(**A**) Gating strategy to identify live cells as Live/Dead dye$^-$ Annexin V$^-$, apoptotic cells as Live/Dead dye$^-$ Annexin V$^+$ and dead cells as Live/Dead dye$^+$ Annexin V$^+$ from total CD4$^+$ cells, and then blasting cells among live cells. (**B**) Distribution of dead, apoptotic and live cells between Th-like Tregs in the presence of exogenous IL-2 (250U/mL) [n=5]. (**C**) Percentages of dead, apoptotic, live and blasting cells in total memory Tregs in the presence of exogenous IL-2 (concentration curve) or (**D**) neutralizing antibodies for IL-2, IL-4, IFN-γ and IL-17 (all at 10μg/mL) [n=4, mean ± SD using bars, RM One-way ANOVA with Dunnet's test]. For all statistical tests **** *P*<0.0001, *** *P*<0.001, ** *P*<0.01 and * *P*<0.05 were considered significant.

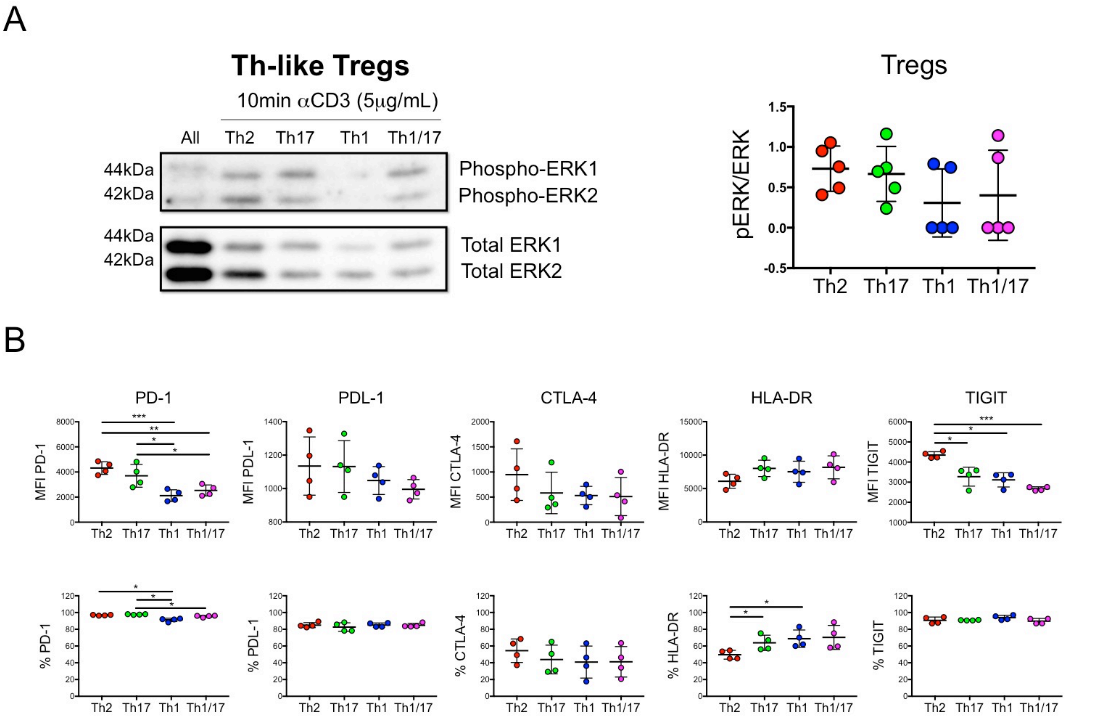

Figure S4: ERK phosphorylation in Th-like Treg and Teff subsets after 10min-post TCR activation, related to Figure 2 and 3.

(A) Representative western blot and absolute number of pERK/ERK ratio between Th-like Tregs. Sorted Th-like Treg subsets were activated with plate bound CD3/CD28 (2µg/mL) on a 96 U-bottom plate for 10min/37°(C). pERK1/2 and ERK1/2 was detected by western blot [n=5, mean ± SEM using independent values, RM One-way ANOVA with Tukey's multiple comparison test]. **(B)** MFI and percentages of PD-1, PDL-1, CTLA-4, HLA-DR and TIGIT in sorted TCR-activated Th-like Treg subsets [n=4, individual values, RM One-way ANOVA with Tukey's multiple comparison test]. For all statistical tests **** *P*<0.0001, *** *P*<0.001, ** *P*<0.01 and * *P*<0.05 were considered significant.

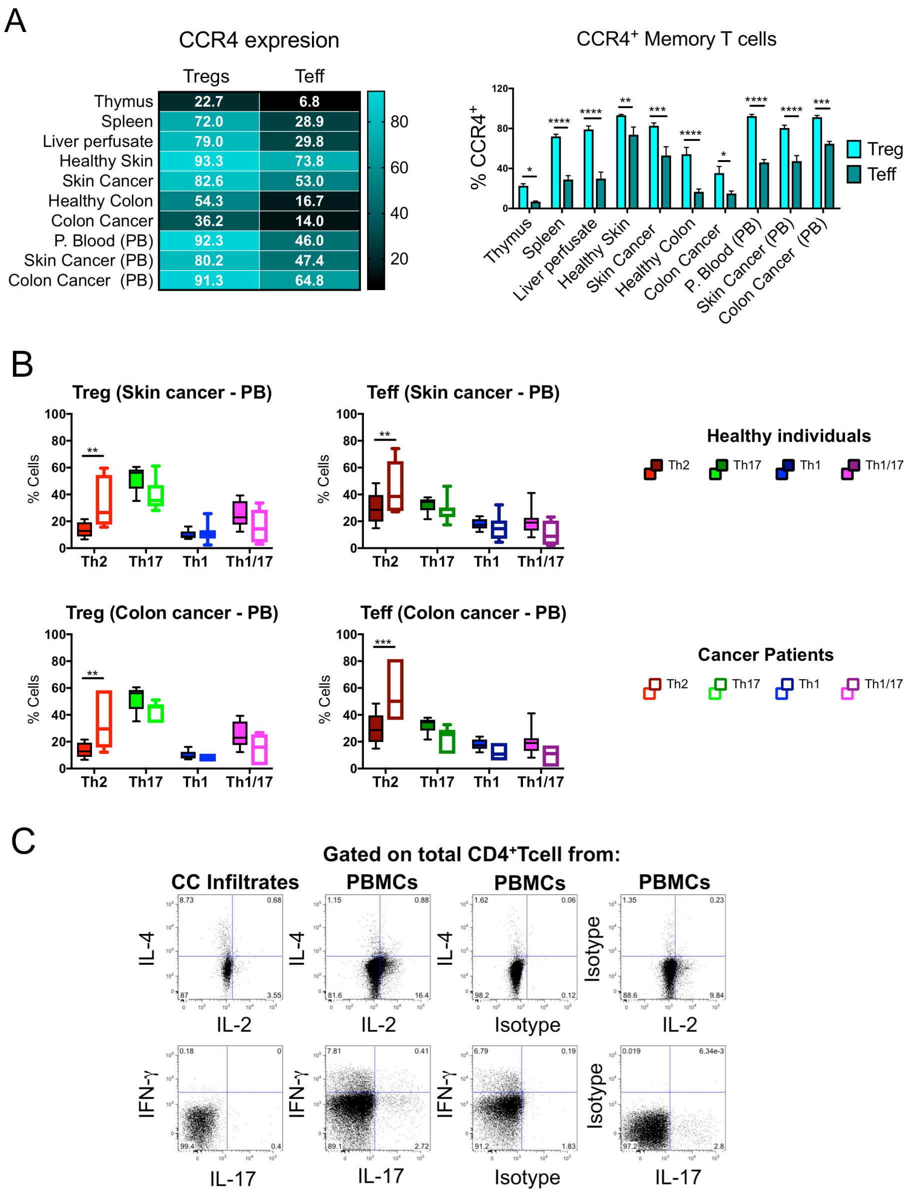

Figure S5: CCR4 expression in Teff and Treg subsets, related to Figure 5.

(**A**) Heat map with average values and total percentages of $CCR4^{+}$ cells within memory Treg or Teff from tissues and peripheral blood (PB) [mean ± SEM using bar charts, Two-way ANOVA with Sidak's test]. (**B**) Th-like distribution between samples obtained from peripheral blood (PB) from healthy individuals and patients with skin or colon cancer [mean ± SEM using box plots, RM Two-way ANOVA with Sidak's multiple comparison test]. **(C)** Intracellular staining of IL-2, IL-4, IFN-γ and IL-17 in total $CD4^{+}$T-cells from colon cancer (CC) area using $CD4^{+}$T-cells from PBMCs and isotypes as positive and negative controls.

Supplemental Tables

Th1, Th2 and Th17 lineage									
	P-Value	Fold Reg.	Th17 vs Th2	P-Value	Fold Reg.	Th1 vs Th2	P-Value	Fold Reg.	Th1/17 vs Th2
GATA3	1.02E-04	-3.06	Th17 down	1.33E-04	-2.79	Th1 down	8.75E-05	-3.24	Th1/17 down
RORA	9.59E-05	2.07	Th17 up				2.08E-03	1.60	Th1/17 up
RORC	1.08E-02	10.95	Th17 up				1.01E-01	6.29	Th1/17 up
TBX21				1.05E-02	4.71	Th1 up	6.77E-02	3.25	Th1/17 up
CCR6	4.65E-06	16.74	Th17 up				3.45E-04	8.41	Th1/17 up
CXCR3				1.51E-03	7.51	Th1 up	1.48E-02	5.00	Th1/17 up
IL4	4.52E-02	-107.28	Th17 down	1.62E-01	-2.67	Th1 down	4.65E-02	-57.32	Th1/17 down
IL5	3.31E-02	-305.20	Th17 down	5.58E-02	-6.97	Th1 down	3.38E-02	-119.08	Th1/17 down
IL13	6.61E-02	-359.98	Th17 down	1.07E-01	-6.29	Th1 down	6.71E-02	-129.86	Th1/17 down
IL17C	1.00E-02	3.24	Th17 up				5.95E-02	2.40	Th1/17 up
IL17A	6.46E-02	6.43	Th17 up				2.88E-01	3.80	Th1/17 up
IFNG				6.49E-02	25.85	Th1 up	4.71E-01	9.48	Th1/17 up

JAK-STAT and T-cell Receptor Signalling Pathways									
	P-Value	Fold Reg.	Th17 vs Th2	P-Value	Fold Reg.	Th1 vs Th2	P-Value	Fold Reg.	Th1/17 vs Th2
BCL2L1	7.44E-02	-1.71	Th17 down				8.62E-02	-1.65	Th1/17 down
CSF2	8.01E-02	-17.48	Th17 down	3.51E-01	-1.83	Th1 down	1.30E-01	-4.69	Th1/17 down
IFNG				6.49E-02	25.85	Th1 up	4.71E-01	9.48	Th1/17 up
IFNGR1							1.43E-03	-1.50	Th1/17 down
IL10RA	1.43E-01	1.50	Th17 up	8.83E-02	1.60	Th1 up			
IL12RB2				3.92E-02	2.51	Th1 up	4.35E-02	2.47	Th1/17 up
IL2	9.32E-02	-3.57	Th17 down	2.31E-01	-1.93	Th1 down			
IL4	4.52E-02	-107.28	Th17 down	1.62E-01	-2.67	Th1 down	4.65E-02	-57.32	Th1/17 down
IL5	3.31E-02	-305.20	Th17 down	5.58E-02	-6.97	Th1 down	3.38E-02	-119.08	Th1/17 down
IL7R	1.28E-01	3.03	Th17 up	6.53E-01	1.54	Th1 up	5.08E-02	3.80	Th1/17 up
IL9	3.09E-02	-162.69	Th17 down	4.12E-02	-12.13	Th1 down	3.26E-02	-49.39	Th1/17 down
LIF	2.65E-02	-5.34	Th17 down	4.11E-02	-3.57	Th1 down	3.42E-02	-4.14	Th1/17 down
SOCS1	6.25E-03	-3.30	Th17 down	3.05E-02	-1.91	Th1 down	7.10E-03	-3.10	Th1/17 down
SOCS3	2.11E-02	-6.09	Th17 down				3.14E-02	-4.05	Th1/17 down
STAT3							5.61E-03	-1.81	Th1/17 down
STAT4				1.35E-03	-1.76	Th1 down			
STAT5A	3.31E-02	-1.52	Th17 down				1.64E-02	-1.69	Th1/17 down
STAT6	1.07E-01	-1.53	Th17 down	9.96E-02	-1.56	Th1 down	4.96E-02	-1.82	Th1/17 down
CDC42							1.13E-02	-1.64	Th1/17 down
NFKBIA	6.41E-02	-2.78	Th17 down	2.67E-01	-1.53	Th1 down	2.04E-01	-1.68	Th1/17 down

Cytokines									
	P-Value	Fold Reg.	Th17 vs Th2	P-Value	Fold Reg.	Th1 vs Th2	P-Value	Fold Reg.	Th1/17 vs Th2
IL1A	5.14E-02	-3.05	Th17 down	1.13E-01	-2.06	Th1 down	1.87E-01	-1.70	Th1/17 down
IL2	9.32E-02	-3.57	Th17 down	2.31E-01	-1.93	Th1 down			
IL6				1.79E-01	-2.62	Th1 down	6.09E-02	-15.80	Th1/17 down
IL7	5.40E-03	2.72	Th17 up	1.40E-01	1.69	Th1 up	9.61E-02	1.80	Th1/17 up
IL9	3.09E-02	-162.69	Th17 down	4.12E-02	-12.13	Th1 down	3.26E-02	-49.39	Th1/17 down

**IL15**	3.86E-01	1.64	Th17 up	3.93E-02	2.80	Th1 up	1.98E-02	3.16	Th1/17 up
**IL21**	1.56E-02	-15.16	Th17 down	1.90E-01	-1.71	Th1 down	1.82E-02	-10.01	Th1/17 down
**IL24**	2.48E-02	-9.36	Th17 down	4.66E-02	-4.00	Th1 down	2.04E-02	-16.29	Th1/17 down
**IFNG**				6.49E-02	25.85	Th1 up	4.71E-01	9.48	Th1/17 up
**Pro-apoptotic genes**									
	**P-Value**	**Fold Reg.**	**Th17 vs Th2**	**P-Value**	**Fold Reg.**	**Th1 vs Th2**	**P-Value**	**Fold Reg.**	**Th1/17 vs Th2**
**CASP1**	2.22E-02	1.88	Th17 up	7.50E-02	1.62	Th1 up	1.19E-02	2.02	Th1/17 up
**CASP8**	1.85E-02	1.54	Th17 up						
**CD27**	1.04E-01	2.12	Th17 up	5.05E-02	2.43	Th1 up	4.34E-02	2.49	Th1/17 up
**CD40**	9.52E-02	-1.55	Th17 down						
**FADD**							1.48E-02	-1.98	Th1/17 down
**FASLG**	2.35E-02	-2.62	Th17 down				4.35E-02	-2.09	Th1/17 down
**TNFRSF10A**	1.42E-02	1.69	Th17 up				2.89E-02	1.57	Th1/17 up
**TNFRSF8**	6.10E-02	-2.29	Th17 down	2.16E-01	-1.51	Th1 down	4.79E-02	-2.54	Th1/17 down
**TP53**							1.23E-02	-2.11	Th1/17 down
**Anti-apoptotic genes**									
	**P-Value**	**Fold Reg.**	**Th17 vs Th2**	**P-Value**	**Fold Reg.**	**Th1 vs Th2**	**P-Value**	**Fold Reg.**	**Th1/17 vs Th2**
**CD40LG**	8.44E-02	-1.94	Th17 down	1.13E-01	-1.77	Th1 down	6.13E-02	-2.17	Th1/17 down
**CSF2**	8.01E-02	-17.48	Th17 down	3.51E-01	-1.83	Th1 down	1.30E-01	-4.69	Th1/17 down
**IL2**	9.32E-02	-3.57	Th17 down	2.31E-01	-1.93	Th1 down			
**IL4**	4.52E-02	-107.28	Th17 down	1.62E-01	-2.67	Th1 down	4.65E-02	-57.32	Th1/17 down
**IL6**				1.79E-01	-2.62	Th1 down	6.09E-02	-15.80	Th1/17 down
**MYC**	7.61E-03	-2.44	Th17 down	1.39E-02	-2.06	Th1 down	5.27E-03	-2.77	Th1/17 down
**PTGDR2**	1.11E-02	-10.94	Th17 down	1.29E-02	-8.15	Th1 down	8.28E-03	-35.36	Th1/17 down
**NFKB2**	5.19E-02	-2.17	Th17 down	1.31E-01	-1.64	Th1 down	6.48E-02	-2.01	Th1/17 down
**TNFRSF4**	1.67E-02	-2.87	Th17 down	3.39E-02	-2.18	Th1 down	2.51E-02	-2.43	Th1/17 down
**Transcription Factors**									
	**P-Value**	**Fold Reg.**	**Th17 vs Th2**	**P-Value**	**Fold Reg.**	**Th1 vs Th2**	**P-Value**	**Fold Reg.**	**Th1/17 vs Th2**
**CEBPB**	3.50E-02	-2.86	Th17 down				4.06E-02	-2.65	Th1/17 down
**FOSL1**	5.95E-02	-2.35	Th17 down	1.41E-01	-1.72	Th1 down	9.21E-02	-1.98	Th1/17 down
**GATA3**	1.02E-04	-3.06	Th17 down	1.33E-04	-2.79	Th1 down	8.75E-05	-3.24	Th1/17 down
**IRF1**				1.10E-03	1.89	Th1 up	1.99E-03	1.80	Th1/17 up
**IRF4**	2.60E-03	-2.42	Th17 down	2.62E-02	-1.53	Th1 down	2.16E-03	-2.55	Th1/17 down
**IRF8**	8.15E-02	-7.11	Th17 down				8.39E-02	-6.71	Th1/17 down
**NFATC1**							2.31E-04	-1.68	Th1/17 down
**RORA**	9.59E-05	2.07	Th17 up				2.08E-03	1.60	Th1/17 up
**RORC**	1.08E-02	10.95	Th17 up				1.01E-01	6.29	Th1/17 up
**RUNX1**	6.53E-02	2.05	Th17 up				2.26E-01	1.63	Th1/17 up
**RUNX3**	1.57E-02	-1.64	Th17 down				4.06E-02	-2.65	Th1/17 down
**TBX21**				1.05E-02	4.71	Th1 up	6.77E-02	3.25	Th1/17 up
**Cytokines Receptors**									
	**P-Value**	**Fold Reg.**	**Th17 vs Th2**	**P-Value**	**Fold Reg.**	**Th1 vs Th2**	**P-Value**	**Fold Reg.**	**Th1/17 vs Th2**
**IL1R1**	3.11E-02	2.02	Th17 up						
**IL1RAP**	1.77E-02	-2.27	Th17 down	7.07E-02	-1.61	Th1 down	1.38E-02	-2.46	Th1/17 down
**IL1RL1**	7.20E-02	-12.93	Th17 down	8.20E-02	-8.53	Th1 down	6.76E-02	-17.31	Th1/17 down

**IL1RN**	7.93E-02	-2.50	Th17 down	1.15E-01	-2.09	Th1 down	2.29E-01	-1.61	Th1/17 down
**IL2RA**	3.71E-02	-1.74	Th17 down	5.08E-02	-1.64	Th1 down	1.93E-02	-2.03	Th1/17 down
**IL3RA**	7.96E-02	-7.15	Th17 down	1.06E-01	-4.46	Th1 down	6.55E-02	-12.14	Th1/17 down
**IL4R**	3.81E-03	-1.78	Th17 down	1.93E-03	-2.01	Th1 down	1.70E-03	-2.06	Th1/17 down
**IL6R**	2.63E-02	1.72	Th17 up	1.35E-02	1.84	Th1 up			
**IL7R**	1.28E-01	3.03	Th17 up	6.53E-01	1.54	Th1 up	5.08E-02	3.80	Th1/17 up
**IL9R**	1.25E-02	-10.82	Th17 down	2.60E-02	-4.13	Th1 down	1.07E-02	-16.97	Th1/17 down
**IL10RA**	1.43E-01	1.50	Th17 up	8.83E-02	1.60	Th1 up			
**IL12RB1**	4.79E-02	1.60	Th17 up						
**IL17RA**	1.18E-01	1.63	Th17 up	1.61E-01	1.55	Th1 up	7.44E-02	1.75	Th1/17 up
**IL17RB**	4.94E-03	-3.61	Th17 down	1.39E-03	-15.17	Th1 down	1.44E-03	-13.99	Th1/17 down
**IL17RE**	3.39E-02	2.78	Th17 up	5.58E-01	-1.68	Th1 down			
**IFNGR2**	5.41E-04	-2.33	Th17 down	5.02E-05	-7.90	Th1 down	4.58E-05	-8.89	Th1/17 down
**LEPR**	7.44E-04	1.89	Th17 up				7.91E-03	1.55	Th1/17 up
**LTA**	5.63E-02	-3.91	Th17 down	1.85E-01	-1.90	Th1 down	1.35E-01	-2.19	Th1/17 down
**TNFSF4**	6.54E-03	1.91	Th17 up	5.91E-02	1.52	Th1 up			
**TNFSF11**	1.22E-02	-5.20	Th17 down	2.36E-02	-3.19	Th1 down	9.27E-03	-7.11	Th1/17 down
**TNFSF13**							6.79E-02	-1.73	Th1/17 down
**TNFSF13B**	3.92E-02	4.80	Th17 up	1.71E-01	3.25	Th1 up	1.34E-02	6.02	Th1/17 up
**TNFSF14**	2.33E-02	-3.05	Th17 down	1.80E-02	-3.53	Th1 down	4.04E-02	-2.38	Th1/17 down
**TNFRSF14**				1.53E-03	1.65	Th1 up			

Table S1: Inflammatory & immunity transcriptome data set of activated Th-like Treg subsets: Lineage and activation pathways, related to Figure 1, 2 and 6.
Heat map table showing p values and fold-regulation of RNA-Seq data set obtained from Th-like Treg subsets 3 days post-TCR activation using Partek® Software. Th17, Th1 and Th1/17 Th-like Treg subsets were Test Groups, whereas Th2-like Tregs was the Control Group. *Exclusion criteria:* Genes with fold change <1.5 or with average molecular tag count <10, in both the Control and Test Groups or with p value higher than 0.1 in the three Test Groups vs Control are not shown in this table. *Colours:* **RED** represents higher expression in Th2-like Tregs, **GREEN** higher expression in Th17-like Tregs, **BLUE** higher expression in Th1-like Tregs and **PINK** higher expression in Th1/17-like Tregs. Genes were clustered according to the Human Inflammation & Immunity Transcriptome gene list and Partek® Pathway.

Leukocyte trans-endothelial migration									
	P-Value	Fold Reg.	Th17 vs Th2	P-Value	Fold Reg.	Th1 vs Th2	P-Value	Fold Reg.	Th1/17 vs Th2
CDC42							1.13E-02	-1.64	Th1/17 down
CXCR4				4.66E-04	-1.66	Th1 down			
ICAM1	3.99E-02	-3.65	Th17 down	2.35E-01	-1.58	Th1 down	7.78E-02	-2.44	Th1/17 down
ITGAM	5.51E-02	-3.34	Th17 down				1.00E-01	-2.34	Th1/17 down
ITGB2							3.01E-02	-1.69	Th1/17 down
RAC1							7.71E-03	-1.54	Th1/17 down
VAV1							1.66E-02	-1.90	Th1/17 down

Chemokines Receptors									
	P-Value	Fold Reg.	Th17 vs Th2	P-Value	Fold Reg.	Th1 vs Th2	P-Value	Fold Reg.	Th1/17 vs Th2
CCR2	1.81E-01	6.36	Th17 up	3.15E-02	10.88	Th1 up	2.71E-02	11.29	Th1/17 up
CCR5	8.39E-02	4.67	Th17 up	2.61E-02	6.20	Th1 up	1.02E-01	4.41	Th1/17 up
CCR6	4.65E-06	16.74	Th17 up				3.45E-04	8.41	Th1/17 up
CCR9	6.25E-02	1.82	Th17 up						
CXCR3				1.51E-03	7.51	Th1 up	1.48E-02	5.00	Th1/17 up
CXCR4				4.66E-04	-1.66	Th1 down			
CXCR5	7.21E-02	-2.46	Th17 down				2.73E-02	-4.76	Th1/17 down
CXCR6	3.38E-03	2.76	Th17 up	1.84E-02	2.20	Th1 up	2.00E-02	2.18	Th1/17 up

Chemokines									
	P-Value	Fold Reg.	Th17 vs Th2	P-Value	Fold Reg.	Th1 vs Th2	P-Value	Fold Reg.	Th1/17 vs Th2
CCL3	6.14E-02	-9.73	Th17 down				1.36E-01	-3.05	Th1/17 down
CCL17	2.73E-02	-20.63	Th17 down	6.94E-02	-3.62	Th1 down	4.16E-02	-6.53	Th1/17 down
CCL24	2.42E-02	-3.19	Th17 down	1.31E-01	-1.67	Th1 down	1.10E-02	-5.92	Th1/17 down
CXCL8	1.25E-01	-2.24	Th17 down	3.04E-02	-8.05	Th1 down	6.42E-02	-3.37	Th1/17 down
CXCL13	9.79E-02	8.59	Th17 up				8.18E-01	1.93	Th1/17 up
CXCL16	2.82E-02	-2.65	Th17 down	6.08E-02	-1.99	Th1 down	3.51E-02	-2.42	Th1/17 down

Innate & Adaptive Immune Responses									
	P-Value	Fold Reg.	Th17 vs Th2	P-Value	Fold Reg.	Th1 vs Th2	P-Value	Fold Reg.	Th1/17 vs Th2
C3	3.31E-03	-8.63	Th17 down	6.06E-03	-4.52	Th1 down	2.48E-03	-15.98	Th1/17 down
C3AR1	1.05E-02	4.03	Th17 up	6.84E-02	2.83	Th1 up	7.85E-02	2.75	Th1/17 up
CD14	3.16E-02	-5.67	Th17 down	5.73E-02	-3.26	Th1 down	3.93E-02	-4.45	Th1/17 down
CD8A	3.70E-02	-13.79	Th17 down	7.11E-02	-4.17	Th1 down	4.94E-02	-6.79	Th1/17 down
DDX58	8.82E-03	1.85	Th17 up	3.65E-02	1.60	Th1 up	2.67E-02	1.65	Th1/17 up
ELK1	6.23E-04	-1.68	Th17 down				1.92E-04	-2.01	Th1/17 down
GZMA				1.47E-03	4.76	Th1 up			
HLA-C							2.38E-02	1.52	Th1/17 up
HLA-E	6.34E-03	1.79	Th17 up				2.47E-03	1.96	Th1/17 up
MX1	1.08E-02	2.48	Th17 up	3.82E-02	2.07	Th1 up	1.88E-02	2.29	Th1/17 up
NOD2	2.11E-02	-2.57	Th17 down						
TLR9							5.02E-02	-1.63	Th1/17 down
CHUK							1.33E-02	-1.51	Th1/17 down
HMGB1							8.68E-02	-1.76	Th1/17 down
HSPD1	2.76E-03	-2.02	Th17 down	5.33E-03	-1.78	Th1 down	1.32E-03	-2.40	Th1/17 down

MAP3K1	3.79E-02	2.71	Th17 up				3.02E-02	2.82	Th1/17 up
MAP2K3							9.10E-02	-1.66	Th1/17 down
RELB	6.29E-02	-1.80	Th17 down				8.59E-02	-1.67	Th1/17 down
UBE2N							4.69E-02	-1.58	Th1/17 down
PRKCZ	7.54E-03	1.87	Th17 up						
PRKRA							4.95E-03	-1.75	Th1/17 down
**Other Genes**									
	**P-Value**	**Fold Reg.**	**Th17 vs Th2**	**P-Value**	**Fold Reg.**	**Th1 vs Th2**	**P-Value**	**Fold Reg.**	**Th1/17 vs Th2**
ACKR3				5.29E-02	-1.65	Th1 down			
BST2							3.96E-02	-1.61	Th1/17 down
BTLA	8.80E-03	-1.76	Th17 down				6.55E-03	-1.85	Th1/17 down
CBLB							4.52E-02	1.55	Th1/17 up
CD2	1.38E-02	1.53	Th17 up						
CD274	8.80E-03	-1.95	Th17 down	4.46E-03	-2.29	Th1 down	7.43E-03	-2.02	Th1/17 down
CD276	2.74E-02	-2.22	Th17 down						
CD44	2.00E-03	1.88	Th17 up				2.93E-03	1.82	Th1/17 up
CD81							5.45E-02	-1.61	Th1/17 down
CDK2	2.36E-03	-2.22	Th17 down	6.74E-03	-1.79	Th1 down	6.22E-04	-3.44	Th1/17 down
CDKN1B	6.91E-03	1.54	Th17 up						
CIITA							6.79E-02	-1.74	Th1/17 down
CSF1R	2.36E-01	-1.64	Th17 down				7.86E-02	-2.68	Th1/17 down
EOMES				3.12E-02	13.07	Th1 up			
FOXP1	4.56E-03	1.54	Th17 up				3.65E-03	1.57	Th1/17 up
GBP1				3.07E-03	1.88	Th1 up	4.08E-04	2.31	Th1/17 up
GPI	9.90E-03	-1.82	Th17 down	2.21E-02	-1.59	Th1 down	3.97E-03	-2.22	Th1/17 down
HDAC9							2.50E-02	-1.56	Th1/17 down
HIF1A							2.33E-02	-1.51	Th1/17 down
IFI30	7.54E-03	-2.94	Th17 down				1.34E-02	-2.38	Th1/17 down
IFI44	4.09E-03	3.56	Th17 up	3.37E-01	1.59	Th1 up	1.33E-02	2.97	Th1/17 up
IFI6	3.31E-02	1.81	Th17 up	3.77E-02	1.78	Th1 up	9.87E-02	1.57	Th1/17 up
IFITM2	2.08E-03	-2.19	Th17 down	6.76E-03	-1.74	Th1 down	2.26E-03	-2.14	Th1/17 down
IFITM3	2.44E-02	-4.02	Th17 down	1.09E-01	-1.90	Th1 down	1.80E-02	-5.29	Th1/17 down
IGF1	1.11E-01	-2.56	Th17 down				6.37E-02	-3.88	Th1/17 down
IRGM	7.74E-02	2.01	Th17 up	7.71E-02	2.01	Th1 up	2.82E-01	1.56	Th1/17 up
LAG3	1.50E-01	2.06	Th17 up	4.33E-02	2.64	Th1 up	4.04E-01	1.58	Th1/17 up
LGALS3	1.82E-03	3.33	Th17 up	2.23E-01	1.60	Th1 up	1.41E-02	2.50	Th1/17 up
LRP1	1.46E-02	-7.23	Th17 down	2.84E-02	-3.69	Th1 down			
LYN	3.58E-02	-6.90	Th17 down				3.42E-02	-7.48	Th1/17 down
MET	9.03E-03	-23.37	Th17 down	3.40E-02	-3.23	Th1 down	1.01E-02	-14.91	Th1/17 down
MICA							1.43E-03	1.61	Th1/17 up
MIF	3.59E-02	-1.64	Th17 down	3.02E-02	-1.69	Th1 down	9.03E-03	-2.22	Th1/17 down
MX1	1.08E-02	2.48	Th17 up						
OAS1	2.36E-03	1.98	Th17 up						
OSM	1.22E-02	-3.12	Th17 down	1.53E-02	-2.81	Th1 down	9.71E-03	-3.53	Th1/17 down
PSME2	1.20E-02	1.62	Th17 up						
PTPRC	1.70E-01	2.88	Th17 up	1.84E-01	2.81	Th1 up	5.91E-02	3.80	Th1/17 up
S1PR1	4.97E-02	-2.22	Th17 down	1.47E-01	-1.59	Th1 down	2.20E-02	-3.20	Th1/17 down
SELL	5.73E-02	1.79	Th17 up	1.07E-01	1.63	Th1 up			

**SH2D1A**	5.84E-03	-3.99	Th17 down	4.08E-02	-1.87	Th1 down	7.02E-03	-3.57	Th1/17 down
**TAP2**							2.34E-02	-1.53	Th1/17 down
**TIMP1**	6.36E-03	-2.99	Th17 down	1.26E-02	-2.33	Th1 down	1.31E-02	-2.30	Th1/17 down
**TP53INP1**	3.12E-02	2.01	Th17 up	4.23E-02	1.93	Th1 up	2.08E-02	2.12	Th1/17 up
**TXLNA**							1.41E-02	-1.87	Th1/17 down
**TYK2**							9.31E-02	-1.52	Th1/17 down
**UTS2**	4.24E-02	2.33	Th17 up	3.45E-01	1.53	Th1 up	2.99E-02	2.47	Th1/17 down
**VEGFA**	8.98E-02	-1.86	Th17 down	5.92E-02	-2.13	Th1 down	3.73E-02	-2.56	Th1/17 down
**XCR1**				8.06E-02	2.50	Th1 up			
**ZBTB7B**							5.85E-02	-1.65	Th1/17 down

Table S2: Inflammatory & immunity transcriptome data set of activated Th-like Treg subsets: Migration pathways, related to Figure 4 and 6.

Heat map table showing p values and fold-regulation of RNA-Seq data set obtained from Th-like Treg subsets 3 days post-TCR activation using Partek® Software. Th17, Th1 and Th1/17 Th-like Treg subsets were Test Groups, whereas Th2-like Tregs was the Control Group. *Exclusion criteria:* Genes with fold change <1.5 or with average molecular tag count <10, in both the Control and Test Groups or with p value higher than 0.1 in the three Test Groups vs Control are not shown in this table. *Colours:* **RED** represents higher expression in Th2-like Tregs, **GREEN** higher expression in Th17-like Tregs, **BLUE** higher expression in Th1-like Tregs and **PINK** higher expression in Th1/17-like Tregs. Genes were clustered according to the Human Inflammation & Immunity Transcriptome gene list and Partek® Pathway.

Patient	Tissue of Cance	Type of Cancer	Cancer location	Stage AJCC	Age	Gender	Tissue	PBMC	Therapy
1	Skin	Melanoma	Lower leg	IV	84	Male	Yes	No	None
2	Skin	Melanoma	Upper arm	IV	59	Male	Yes	Yes	None
3	Skin	Melanoma	Elbow	IV	77	Female	Yes	Yes	None
4	Skin	Melanoma	Chest Wall	IV	65	Male	No	Yes	None
5	Skin	Melanoma	Left Temple	IV	89	Male	No	Yes	None
6	Skin	Melanoma	Upper arm	IV	28	Female	No	Yes	Dabrafenib
7	Skin	Melanoma	Post Auricular	IV	28	Female	No	Yes	Dabrafenib Trametinib
8	Skin	Melanoma	Leg	IIIC	61	Female	Yes	No	None
9	Skin	Melanoma	Abdomen	IV	70	Female	No	Yes	None
10	Skin	Melanoma	Chest wall	IV	56	Male	No	Yes	None
11	Skin	Melanoma	Unknown	IV	60	Male	No	Yes	None
12	Skin	Melanoma	Leg	IV	70	Female	No	Yes	None
13	Colon	Adenocarcinoma	Sigmoid	I (T1N0M0)	78	Male	Yes	Yes	None
14	Colon	PTLD (B-cell lymphoma)	Sigmoid	N/A	58	Male	Yes	Yes	Tacrolimus, Vedolizumab
15	Colon	Signet ring adenocarcinoma	Rectum	IIIC (T3N2M0)	33	Male	No	Yes	Vedolizumab
16	Colon	Adenocarcinoma	Ascending	IIIC (T4N2M0)	78	Female	Yes	No	None
17	Colon	Adenocarcinoma	Transverse	I (T2N0M0)	69	Male	Yes	Yes	None
18	Colon	Adenocarcinoma	Descending	IIIA (T4N1M0)	55	Female	Yes	Yes	None

Table S3: Description of patient samples, related to Figure 5 and Table 1.
Blood and tissue samples (from and distant from cancer area) were collected from consecutive patients requiring surgery for melanoma colorectal cancer. Pharmacological treatment was stopped at least two weeks before surgery. **AJCC:** American Joint Committee on Cancer. **PTLD:** Post-transplant lymphoproliferative disease. **N/A:** non applicable. Classification of Malignant Tumours (TNM) in colorectal cancer: **T** describes the size of tumor as T0: no signs of tumor, T1: Tumor in lamina propria or submusoca or $\leq$ 2cm, T2: Muscularis propria or > 2cm, T3: Tumor in subserosa, or pericolorectal tissues and T4: Tumor perforates serosa; adjacent structures. **N** describes nearby (regional) lymph

nodes involved: N0: no lymph nodes, N1: ≤ 3 regional lymph nodes and N2: > 3 regional lymph nodes.
M describes distant metastasis: M0: no metastasis.

	#	P-Value	P-Value	Fold Change	Descrip-tion	P-Value	Fold Change	Descrip-tion	P-Value	Fold Change	Descrip-tion
Pathway Name	Probe Sets	Th Type	Th17 vs. Th2	Th17 vs. Th2	Th17 vs. Th2	Th1 vs. Th2	Th1 vs. Th2	Th1 vs. Th2	Th1/17 vs. TH2	Th1/17 vs. TH2	Th1/17 vs. TH2
Endometrial cancer	6	2.2E-03	2.4E-03	-1.55	TH17 down vs TH2	3.2E-03	-1.5	TH1 down vs TH2	4.2E-04	-1.99	TH1-TH17 down vs TH2
Huntington's disease	3	1.1E-02	1.0E-02	1.18	TH17 up vs TH2	6.6E-01	1.0	TH1 up vs TH2	2.0E-01	-1.08	TH1-TH17 down vs TH2
Thyroid cancer	4	2.0E-02	3.1E-02	-1.43	TH17 down vs TH2	1.3E-02	-1.6	TH1 down vs TH2	4.4E-03	-1.91	TH1-TH17 down vs TH2
MicroRNAs in cancer	24	2.3E-02	1.7E-02	-1.31	TH17 down vs TH2	2.2E-02	-1.3	TH1 down vs TH2	5.1E-03	-1.45	TH1-TH17 down vs TH2
Pancreatic cancer	19	2.4E-02	1.1E-02	-1.26	TH17 down vs TH2	4.4E-02	-1.2	TH1 down vs TH2	6.4E-03	-1.30	TH1-TH17 down vs TH2
Colorectal cancer	10	3.6E-02	4.1E-02	-1.28	TH17 down vs TH2	2.3E-02	-1.3	TH1 down vs TH2	8.2E-03	-1.48	TH1-TH17 down vs TH2
Viral carcinogenesis	39	4.0E-02	3.5E-02	1.25	TH17 up vs TH2	9.2E-02	1.2	TH1 up vs TH2	8.1E-03	1.36	TH1-TH17 up vs TH2
Acute myeloid leukemia	12	4.2E-02	4.3E-02	-1.30	TH17 down vs TH2	3.6E-02	-1.3	TH1 down vs TH2	9.0E-03	-1.53	TH1-TH17 down vs TH2
Type II diabetes mellitus	7	4.6E-02	1.3E-02	-2.46	TH17 down vs TH2	6.6E-02	-1.6	TH1 down vs TH2	1.9E-02	-2.20	TH1-TH17 down vs TH2
Epstein-Barr virus infection	45	4.6E-02	5.4E-02	1.24	TH17 up vs TH2	7.9E-02	1.2	TH1 up vs TH2	8.8E-03	1.39	TH1-TH17 up vs TH2
Herpes simplex infection	74	4.6E-02	7.9E-02	1.18	TH17 up vs TH2	7.3E-02	1.2	TH1 up vs TH2	8.6E-03	1.34	TH1-TH17 up vs TH2
Non-alcoholic fatty liver disease	24	4.8E-02	2.9E-02	-1.32	TH17 down vs TH2	5.4E-02	-1.3	TH1 down vs TH2	1.1E-02	-1.44	TH1-TH17 down vs TH2
Pathogenic Escherichia coli infection	7	5.4E-02	1.6E-01	-1.17	TH17 down vs TH2	6.0E-01	-1.1	TH1 down vs TH2	1.4E-02	-1.47	TH1-TH17 down vs TH2
Renal cell carcinoma	10	5.5E-02	6.2E-02	-1.28	TH17 down vs TH2	4.0E-02	-1.3	TH1 down vs TH2	1.2E-02	-1.51	TH1-TH17 down vs TH2
Proteoglycans in cancer	29	5.6E-02	4.4E-02	-1.30	TH17 down vs TH2	4.1E-02	-1.3	TH1 down vs TH2	1.3E-02	-1.46	TH1-TH17 down vs TH2
Viral myocarditis	19	5.8E-02	5.5E-02	1.29	TH17 up vs TH2	9.2E-02	1.2	TH1 up vs TH2	1.2E-02	1.44	TH1-TH17 up vs TH2
HTLV-I infection	56	5.9E-02	8.2E-02	1.13	TH17 up vs TH2	1.1E-01	1.1	TH1 up vs TH2	1.1E-02	1.23	TH1-TH17 up vs TH2
Basal cell carcinoma	2	6.4E-02	1.0E-01	-1.40	TH17 down vs TH2	1.1E-01	-1.4	TH1 down vs TH2	1.2E-02	-2.11	TH1-TH17 down vs TH2
Non-small cell lung cancer	6	6.6E-02	1.4E-01	-1.26	TH17 down vs TH2	7.5E-02	-1.4	TH1 down vs TH2	1.3E-02	-1.73	TH1-TH17 down vs TH2
Graft-versus-host disease	20	7.2E-02	9.3E-02	1.22	TH17 up vs TH2	8.3E-02	1.2	TH1 up vs TH2	1.4E-02	1.37	TH1-TH17 up vs TH2
Asthma	11	8.0E-02	2.9E-02	-5.23	TH17 down vs TH2	4.8E-02	-3.4	TH1 down vs TH2	3.1E-02	-4.93	TH1-TH17 down vs TH2
Pathways in cancer	57	8.0E-02	2.7E-02	-1.36	TH17 down vs TH2	8.4E-02	-1.2	TH1 down vs TH2	2.9E-02	-1.36	TH1-TH17 down vs TH2
Type I diabetes mellitus	23	8.9E-02	1.4E-01	1.16	TH17 up vs TH2	1.1E-01	1.2	TH1 up vs TH2	1.8E-02	1.30	TH1-TH17 up vs TH2
Bladder cancer	11	1.0E-01	6.6E-02	-1.44	TH17 down vs TH2	5.6E-02	-1.5	TH1 down vs TH2	2.8E-02	-1.65	TH1-TH17 down vs TH2
Autoimmune thyroid disease	26	1.1E-01	1.3E-01	1.19	TH17 up vs TH2	1.3E-01	1.2	TH1 up vs TH2	2.4E-02	1.33	TH1-TH17 up vs TH2
Allograft rejection	24	1.1E-01	1.5E-01	1.19	TH17 up vs TH2	1.2E-01	1.2	TH1 up vs TH2	2.4E-02	1.33	TH1-TH17 up vs TH2
Amyotrophic lateral sclerosis	9	1.5E-01	8.1E-02	-1.29	TH17 down vs TH2	8.6E-02	-1.3	TH1 down vs TH2	4.4E-02	-1.38	TH1-TH17 down vs TH2
Melanoma	6	1.5E-01	7.7E-02	-1.41	TH17 down vs TH2	1.2E-01	-1.3	TH1 down vs TH2	4.1E-02	-1.55	TH1-TH17 down vs TH2
Small cell lung cancer	15	1.5E-01	4.4E-02	-1.73	TH17 down vs TH2	1.5E-01	-1.4	TH1 down vs TH2	7.4E-02	-1.56	TH1-TH17 down vs TH2

Table S4: Pathways ANOVA between Th-like Treg subsets, related to Figure 6.
Results of pathway ANOVA performed using Partek® Genomics Suite® software, version 6.6. Pathways shown are KEGG pathways (number of genes considered as part of pathway in ANOVA

indicated in second column) significantly enriched within at least one comparison of pathway ANOVA (Th17 vs Th2, Th1 vs Th2 or Th1/Th17 vs Th2). Pathways are displayed in ascending order based on overall pathway significance (p-value <0.05 are indicated in orange). *Colours:* **RED** represents higher expression in Th2-like Tregs, **GREEN** higher expression in Th17-like Tregs, **BLUE** higher expression in Th1-like Tregs and **PINK** higher expression in Th1/17-like Tregs (p-value <0.1 are coloured).

Supplemental Experimental Procedures

Culture conditions and sorting strategy

For *in vitro* assays, cells were cultured in X-VIVO15 (Lonza) supplemented with L-Glutamine 2mM, penicillin/streptomycin 100U/mL (both Thermo Fisher) and 10% of Human Serum AB Male (BioWest). Pre-enriched Th-like Treg and Teff were analysed before and after sorting. Th-like Treg and Teff subsets were of >98 purity.

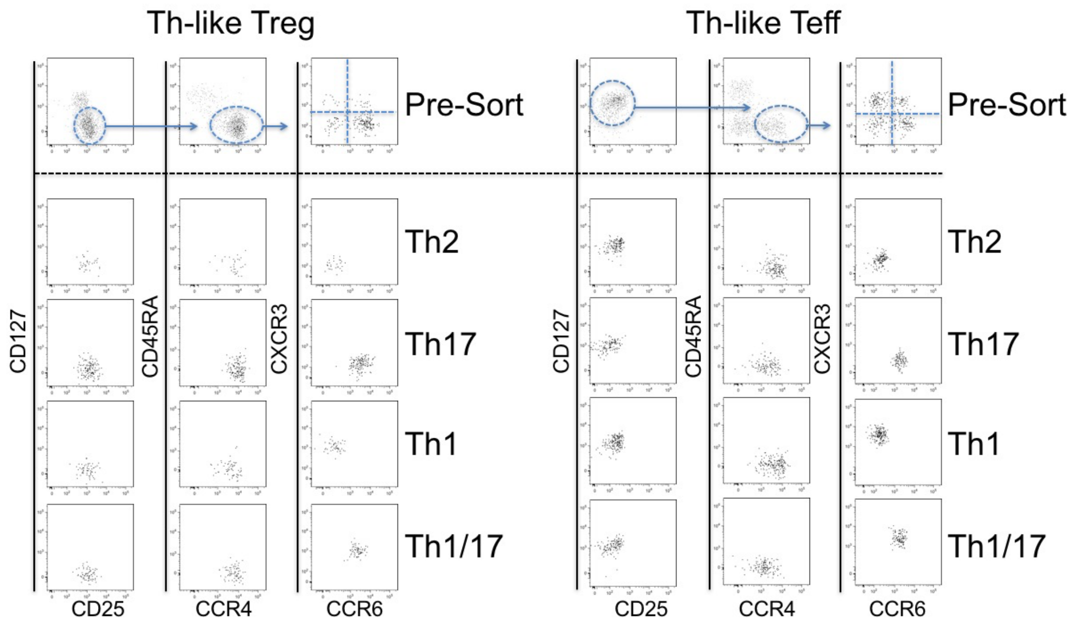

Sorting strategy.

TIGIT, PDL-1, PD-1, HLA-DR, CTLA-4 expression and cytokine analysis

FACS-sorted Th-like Tregs (0.5-1x10^5), total memory Tregs (0.5x10^5) and Teff (1x10^5) were stimulated with anti-CD3/CD28 beads at a 4:1 (cell: bead) ratio. After 72h, TIGIT, PDL-1, PD-1, HLA-DR, CTLA-4 were evaluated using surface staining. Supernatant were used to detect human T-cell cytokine production using LEGENDplex Human Th-Cytokine Assay (BioLegend) and BD Cytometric Bead Array following manufacturer's instructions. Cytokines were acquired on a FACSCanto II (BD Biosciences). Data analysis was carried out on LEGENDplex™ Data Analysis Software or FCAP Array™ Software (BD Biosciences).

ERK Activation and western blot

FACS-sorted subpopulations (1x10^5) were activated with plate bound CD3/CD28 (R&D Systems) (2µg/mL) for 10min/37°C after spin 1800rpm/3min/20°C. Cell lysates were prepared using RIPA buffer (Thermo Fisher) supplemented with protease inhibitors (Calbiochem). Samples were electrophoresed on 10% SDS polyacrylamide gels and transferred to nitrocellulose membranes. After blocking, membranes were incubated with phospho-p44/42 MAPK (Erk1/2) (Thr202/Tyr204) (Cell Signalling) overnight at 4°C. The following day, proteins were detected with chemiluminescence detection reagents (BIORAD) after HRP conjugated secondary antibody incubation using ImageQuant LASS4000 mini (GE Healthcare Life Science), quantified with ImageQuant TL software. Blots were stripped and incubated with p44/42 MAPK (Erk1/2) (Thr202/Tyr204) (Cell Signalling). Same protocol was used for p53 and Stat5 detection.

Intracellular staining

Cells were activated with PMA and Ionomycin for 3h at 37°C. Then, cells were stained with anti-CD4 APC/Cy7, anti-CD3 PerCP/Cy5.5, anti-CD8 Brilliant Violet-711 and anti-CD20 Brilliant Violet-605. Intracellular staining was performed with the Foxp3/Transcription Factor Staining Buffer Set (eBioscience) using anti-FoxP3, anti-IL-2, anti-IL-4, anti-IL-10, anti-IL-17 and anti-IFN-γ 30min/4°C/dark. Samples were then acquired on LSR-Fortessa flow cytometer and files analysed using FlowJo (Tree Star Inc.). Gates were set based on biological, Fluorescent minus one and isotype

controls.

Tissue Collection and Research Ethics Committee

Spleen samples were obtained from deceased human liver or kidney donors and perfusates were obtained from liver grafts at King's College Hospital (both approved by St Thomas' Ethics Committee, reference number: 09/H0802/100). Healthy colon biopsies and colon resections from cancer patients were obtained at King's College Hospital (approved by the London-Dulwich Research Ethics Committee, reference number: 15/LO/1998). Skin was obtained from surgical procedures at Springfield Hospital (approved by the East London and City Health Authority Research Ethics Committee, reference number: 09/HO704/69). Thymuses were collected during infant cardiac surgery at Great Ormond Street Hospital (approved by the Institute of Child Health/Great Ormond Street Hospital Research Ethics Committee, reference number: 07/Q0508/43). Melanoma samples were obtained from surgical procedures at St Thomas (approved by the King's College London and St Thomas' NHS Trust Ethics Committee, reference number: 08/H0804/139 and 16/LO/0366). Informed consent was obtained from all study participants or their representatives in accordance with the Declaration of Helsinki.

-Thymus

Thymuses were washed 3-4 times with PBS, cut into pieces (<0.5cm) and transferred into a gentleMACS tube (Miltenyi Biotec) with X-VIVO15 containing dissociation media (0.2mg/mL of collagenase type XI-S, 0.1mg/mL of DNase1 and 0.5µg/mL Fungizone). Samples were then dissociated using Gentle MACS dissociator (Miltenyi Biotec). Samples were incubated for 15min at 37°C and dissociated again using Gentle MACS dissociator. After dissociation, samples were washed and filtered 3 times using 70µm cell strainers. Cells were counted and $2x10^6$ cells were phenotyped.

-Spleen

Spleens were washed with PBS, cut into pieces <0.5cm and dissociated mechanically with a 2mL syringe in a 50mL Falcon tube containing a 70µm cell strainer. Cells were then washed (2000rpm/10min/4°C), cell pellet was resuspended in PBS and viable splenocytes were isolated using density-gradient centrifugation. Cells were then counted and $2x10^6$ cells were phenotyped.

-Liver Perfusates

Hepatic perfusates were obtained by collecting the second perfusion of the grafts through the portal vein with 1L of saline solution. The collected fluids from the vena cava was stored at 5°C and processed within 6h. Viable hepatic mononuclear cells were isolated using density-gradient centrifugation. Cells were then counted and $2x10^6$ cells were phenotyped.

-Healthy Skin

Skin was washed 3-4 times with PBS, cut into small pieces <0.1cm and transferred into a 20mL pot with X-VIVO15 containing collagenase (1mg/mL) and DNAse (10U/mL). Samples were incubated for 45min at 37°C in agitation. After dissociation, samples were washed, filtered and viable mononuclear cells were isolated using density-gradient centrifugation. Cells were then counted and phenotyped.

-Melanoma (Skin Cancer)

Melanoma specimens were cut in small pieces and mechanically disaggregated using a GentleMACS disociator (Miltenyi Biotec). Single cell suspensions were left overnight at 37°C 5%CO_2 in RPMI media containing 10% Fetal Calf Serum and penicillin/streptomycin 100U/mL. Next day, supernatant samples were collected for cytokine analysis and DNaseI (10U/ml) was added to the cultures for 20min at 37°C. Cells suspensions were then filtered through a 100µm mesh and viable cells were isolated using density-gradient centrifugation. Cells were then counted and phenotyped.

-Colorectal cancer resections (Colon Cancer) and healthy colon biopsies

Colon resections from patients with colorectal cancer and post-transplant lymphoproliferative disease (PLTD in the colon) were obtained after surgery at King's College Hospital. A mucosal sample from the cancer area and a distant sample from the cancer area were obtained and processed similarly to healthy colon biopsies. Briefly, colon mucosa was cut into 3-4mm pieces and incubated with 1mM-EDTA/HBSS 37°C 5%CO_2 for 15min with agitation. Colon samples were then cut into small pieces <1mm and transferred into a 20mL pot with X-VIVO15 containing collagenase (1mg/mL) and DNAse (10U/mL). Samples were incubated for 2h at 37°C in agitation. After dissociation, samples were washed, filtered and viable colonic mononuclear cells were isolated using density-gradient centrifugation. Cells were then counted and $2x10^6$ cells were phenotyped.

Reagents	Catalogue	Company
Agilent High Sensitivity DNA Kit	5067-4626	Agilent Technologies
IL-2 (5344.111) FITC	340448	BD Biosciences
CD4 (SK3) Brilliant Ultra Violet 395	563552	BD Biosciences
Tbet (O4-46) Brilliant Violet 786	564141	BD Biosciences
RORγτ (Q21-559) Alexa Fluor 647	563620	BD Biosciences
CXCR3 (1C6) Alexa Fluor 700	561320	BD Biosciences
CCR8 (433H) [CD198] Brilliant Violet 421	566380	BD Biosciences
CD152 [CTLA-4] (BNI3) APC	555855	BD Biosciences
Stat5 pY694 (47/Stat5(pY694))	611819	BD Biosciences
Stat5 (89/Stat5)	610192	BD Biosciences
BD™ Cytometric Bead Array (CBA) Human Th1/Th2/Th17 cytokine kit	560484	BD Biosciences
Annexin V Alexa Fluor 647	640943	BioLegend
CCR4 (L291H4) PECy7<br>CCR4 (L291H4) PerCP/Cy5.5	359410<br>359406	BioLegend
CCR6 (G034E3) Brilliant Violet 605	353420	BioLegend
CD3 (OKT3) PerCP/Cy5.5	317336	BioLegend
CD4 (OKT4) PerCPCy5.5<br>CD4 (OKT4) Brilliant Violet 421	317428<br>317434	BioLegend
CD8 (SK1) Brilliant Violet 711	344734	BioLegend
CD25 (M-A251) PE	356104	BioLegend
CD45 (HI30) Brilliant Violet 711	304050	BioLegend
CD45RA (H110) APC/Cy7<br>CD45RA (H110) PECy7	304128<br>304126	BioLegend
CD127 (A019D5) APC<br>CD127 (A019D5) Brilliant Violet 711	351318<br>351328	BioLegend
CXCR3 (G025H7) FITC	353704	BioLegend
FoxP3 (259D) Pacific Blue™	320216	BioLegend
GATA3 (16E10A23) Alexa Fluor 647	653810	BioLegend
HLA-DR (L243) Alexa Fluor 647	307621	BioLegend
IL-2 (MQ1-17H12) FITC	500304	BioLegend
LEGENDplex Human Th Cytokine Assay	740001	BioLegend
PD-1 (EH12.2H7) [CD279] Brilliant Violet 421	329919	BioLegend
PDL-1 (29E.2A3) [CD274] [B7-H1] PECy7	329717	BioLegend
Recombinant Human ICAM-1-Fc Chimera	552904	BioLegend
Recombinant Human CCL17 [TARC]	573802	BioLegend
Recombinant Human CCL20 [MIP-3α]	583802	BioLegend
Recombinant Human CXCL10 [IP-10]	573502	BioLegend
Recombinant Human CCL22 [MDC]	584902	BioLegend
TIGIT (A15153G) [VSTM3] APC	372706	BioLegend
Mouse MAb Anti-Human IL-2 (B-G5)	AHC0022	BioSource International
Direct-zol™ RNA MicroPrep Zymo-Spin IC Col.	R2060	Cambridge Bioscience
phospho-p44/42 MAPK [Erk1/2] Thr202/Tyr204	#9101	Cell Signalling
p44/42 MAPK [Erk1/2]	#9102	Cell Signalling
HSP90 (C45G5)	#4877	Cell Signalling
P53 (1C12)	#2524	Cell Signalling
IL-4 (8D4-8) PE	12-7049	eBioscience
IL-17 (eBio64DEC17) PE-Cy7	25-7179	eBioscience
FoxP3/Transcription Factor Staining Buffer	00-5523-00	eBioscience
FoxP3 (PCH10) eFluor 450	48-4776-42	eBioscience
MiSeq Reagent Kit v3	MS-102-3001	Illumina
CD25 MicroBeads II	130-092-983	Milltenyi Biotec
Recombinant IL-2	Proleukin<br>PL 00101/0936	Novartis Pharmaceuticals UK Ltd
The Human Inflammation & Immunity Transcriptome RNA targeted panel 12-Index	RHS-005Z<br>333114	QIAgen
CD3 (UCHT1)	MAB100	R&D

CD28 (37407)	MAB342	R&D
Human IL-4 MAb (34019)	MAB204	R&D
Human IFN-gamma MAb (25718)	MAB285	R&D
Human IL-17 MAb (C41809)	MAB317	R&D
CORNING HTS TRANSWELL-96W	CLS3388-2EA	Sigma-Aldrich
RosetteSep® Human $CD4^+$T Cell Enrich. Cocktail	767 CAD	STEMCELL
CountBright™ Absolute Counting Beads	C36950	Thermo Fisher Scientific
CellTrace™ Violet Cell Proliferation Kit	C34557	Thermo Fisher Scientific
IFN-γ (B27) APC	MHCIFG05	Thermo Fisher Scientific
LIVE/DEAD® Fixable Near-IR Dead Cell Stain Kit	L10119	Thermo Fisher Scientific

Reagents: List of reagents used in this study including clones ( ), alternative names [ ], fluorochromes, catalogue numbers and suppliers.
